# Supplementary figures and images for: Detecting epistasis with the marginal epistasis test in genetic mapping studies of quantitative traits
Source: PLoS Genet. 2017 Jul 26;13(7):e1006869. doi: 10.1371/journal.pgen.1006869 (PMC5550000; doi:10.1371/journal.pgen.1006869)

**A**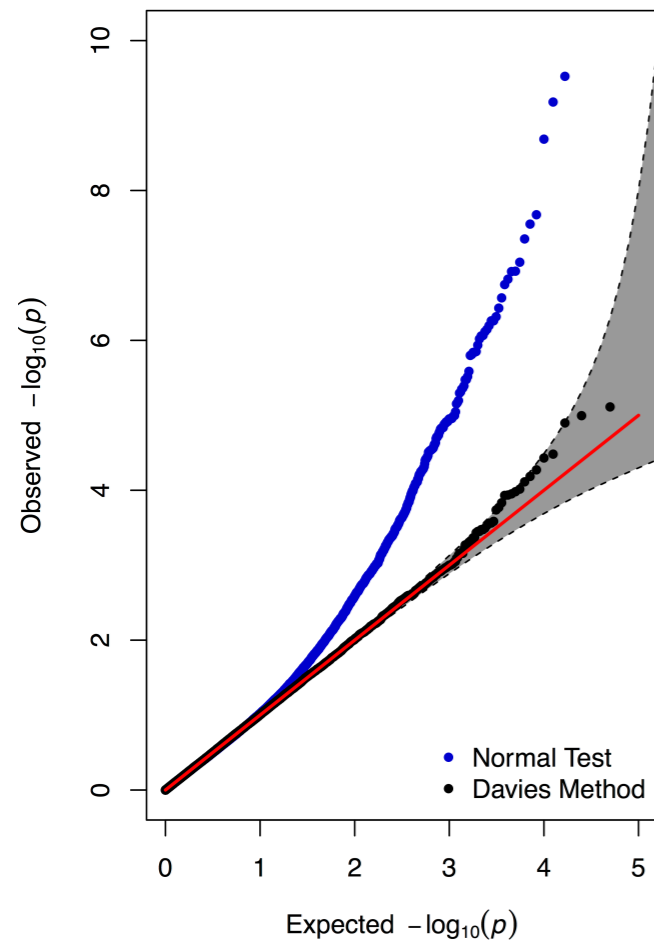**B**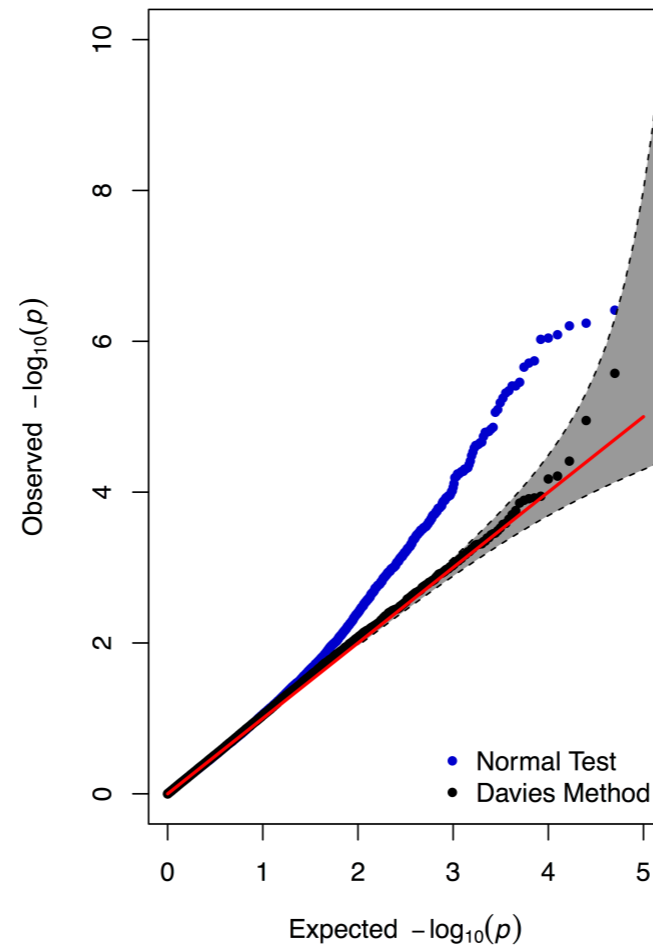**C**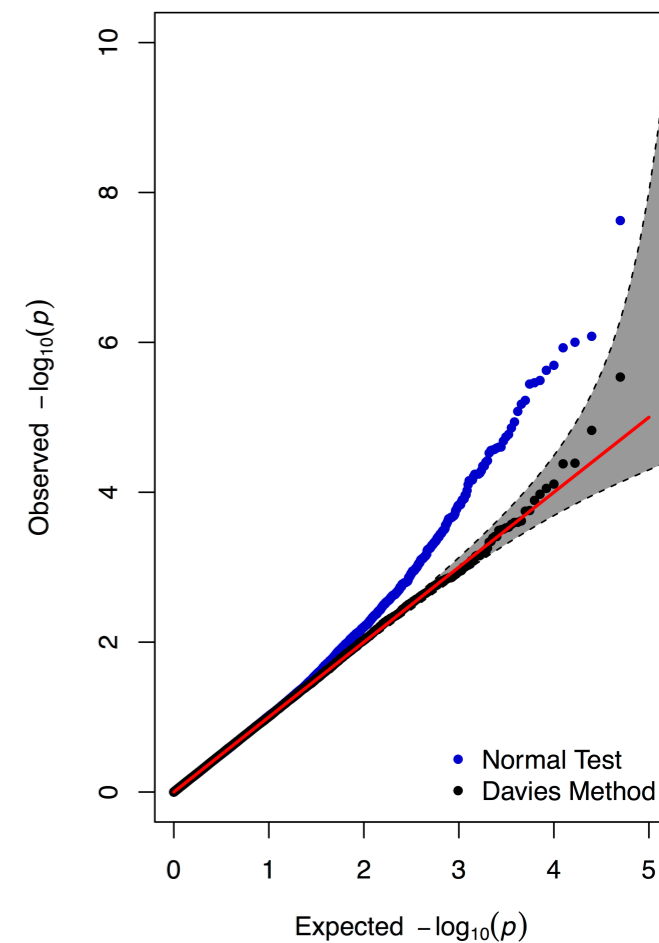**D**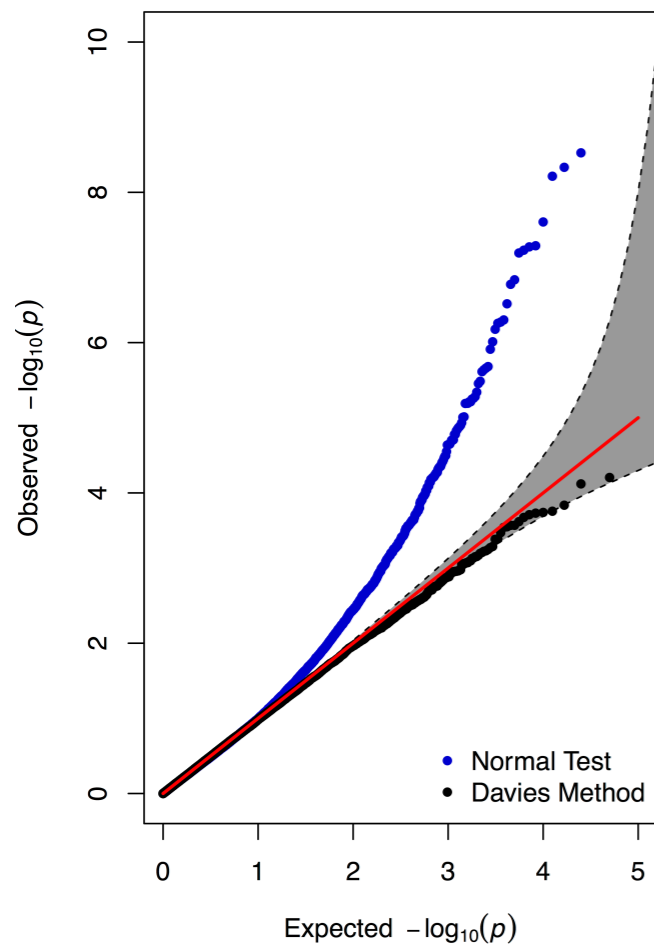**E**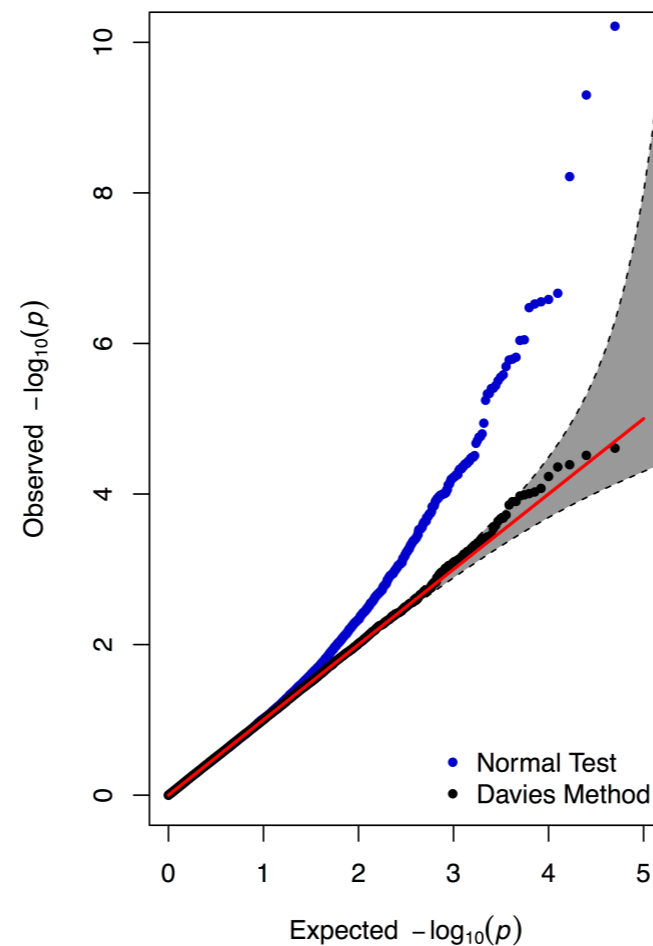**F**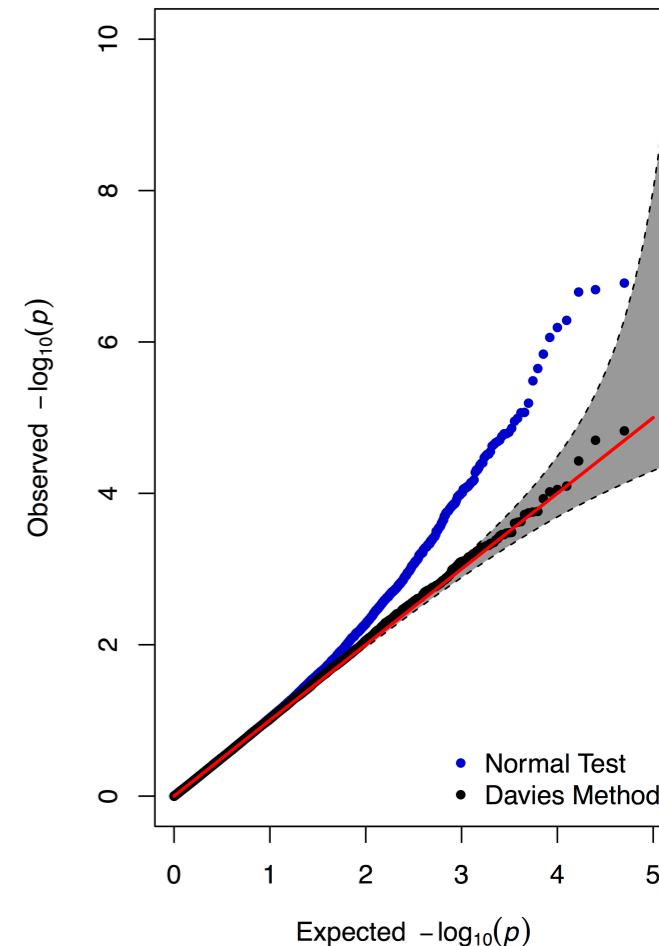

Supplement: S1 Fig — The QQ plots applying MAPIT to 100 simulated null datasets assuming sample sizes: 1,000 (A, D), 1,750 (B, E), and 2,500 (C, F). These results are based on using simulation model (ii). (A)-(C) use the top 5 genotype PCs, while (D)-(F) use the top 10 genotypes PCs. Blue dots are p-values produced by under the normal test (or z-test), while the black dots represent p-values tested using the Davies method via a mixture of chi-square distributions. The 95% confidence intervals for the null hypothesis of no association are shown in grey. (PDF) [file pgen.1006869.s001.pdf]

**A**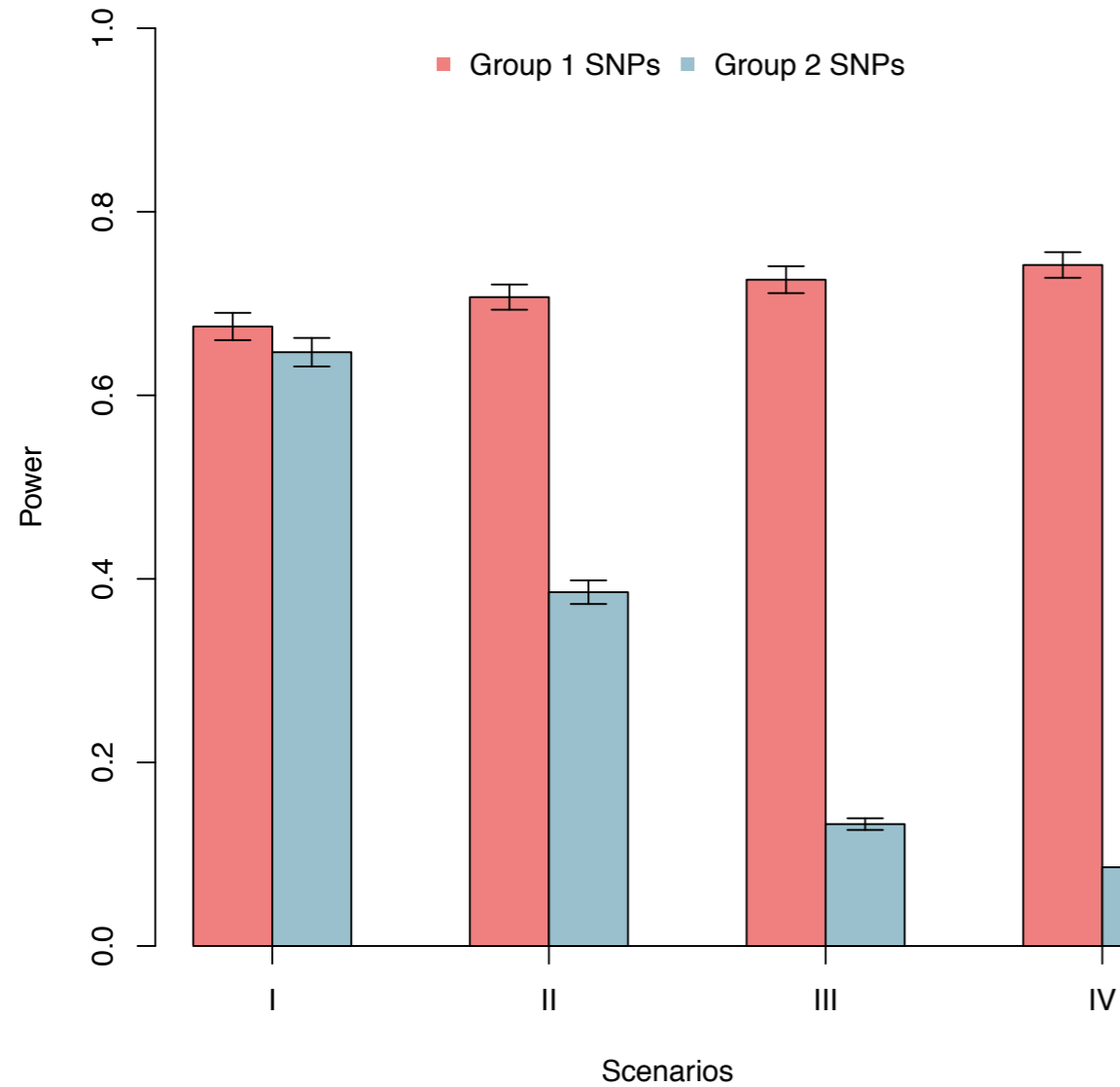**B**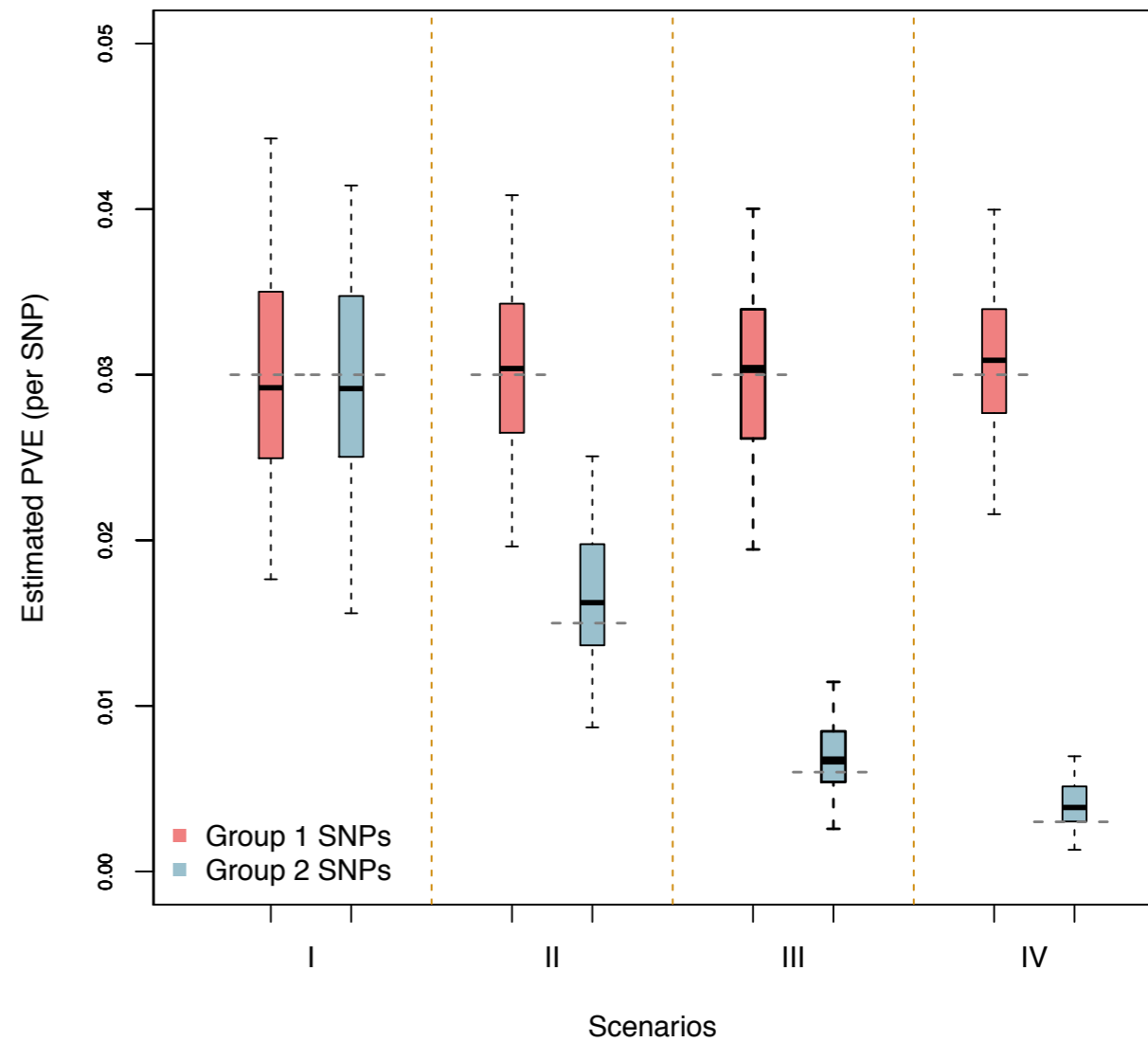

Supplement: S2 Fig — Groups 1 and 2 causal markers are colored in light red and light blue, respectively. These figures are based on a broad-sense heritability level of H2 = 0.6 and parameter ρ = 0.5, estimated with 100 replicates. Here, ρ = 0.5 was used to determine the portion of broad-sense heritability contributed by interaction effects. (A) shows the power of MAPIT to identify SNPs in each causal group under significance level α = 0.05. The lines represent 95% variability due to resampling error. (B) shows boxplots of the marginal PVE estimates for the group 1 and 2 causal SNPs from MAPIT for the four simulation scenarios. The true PVEs per causal SNP (0.03 for the group 1 SNPs; 0.03, 0.015, 0.006, and 0.003 for the group 2 SNPs) are shown as dashed grey horizontal lines. (PDF) [file pgen.1006869.s002.pdf]

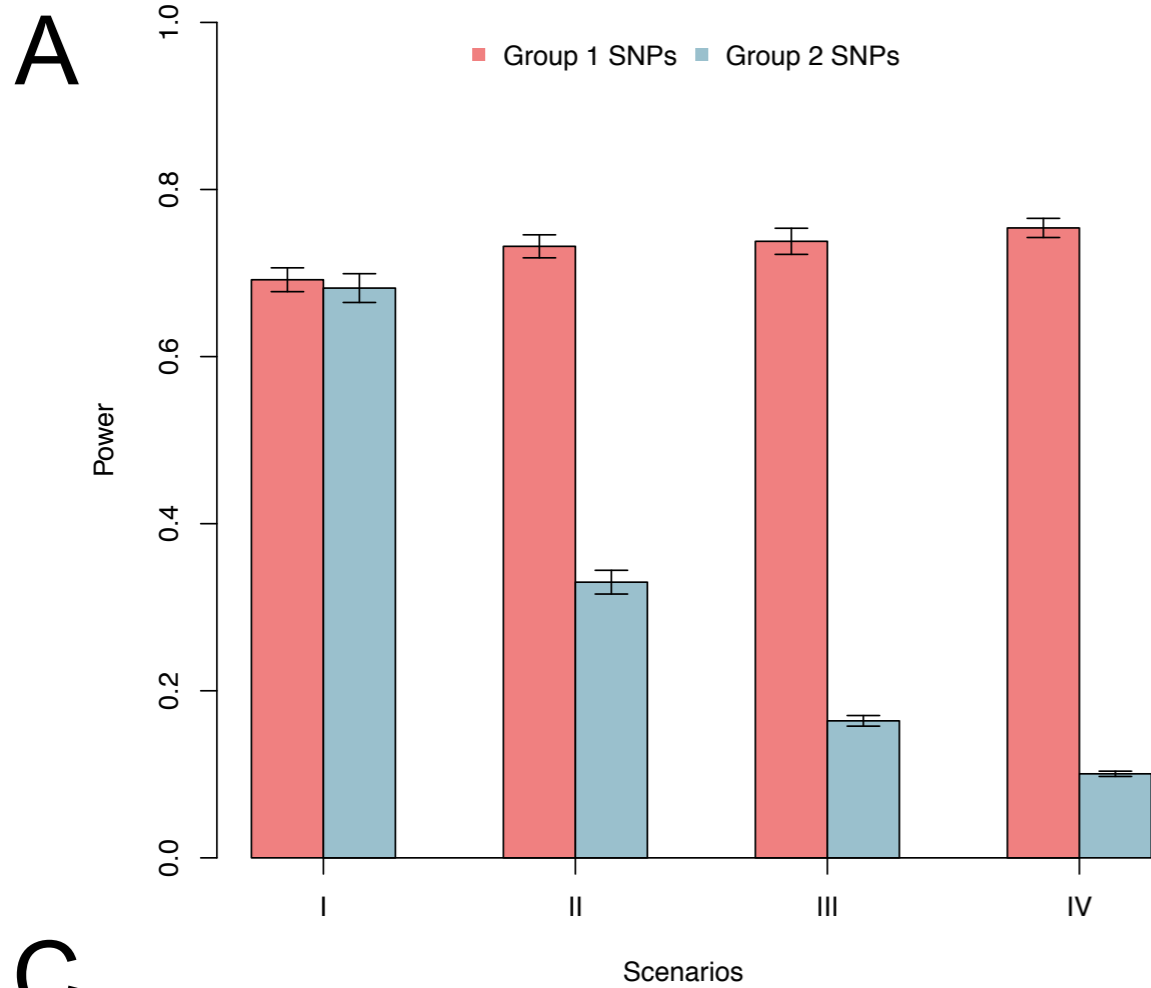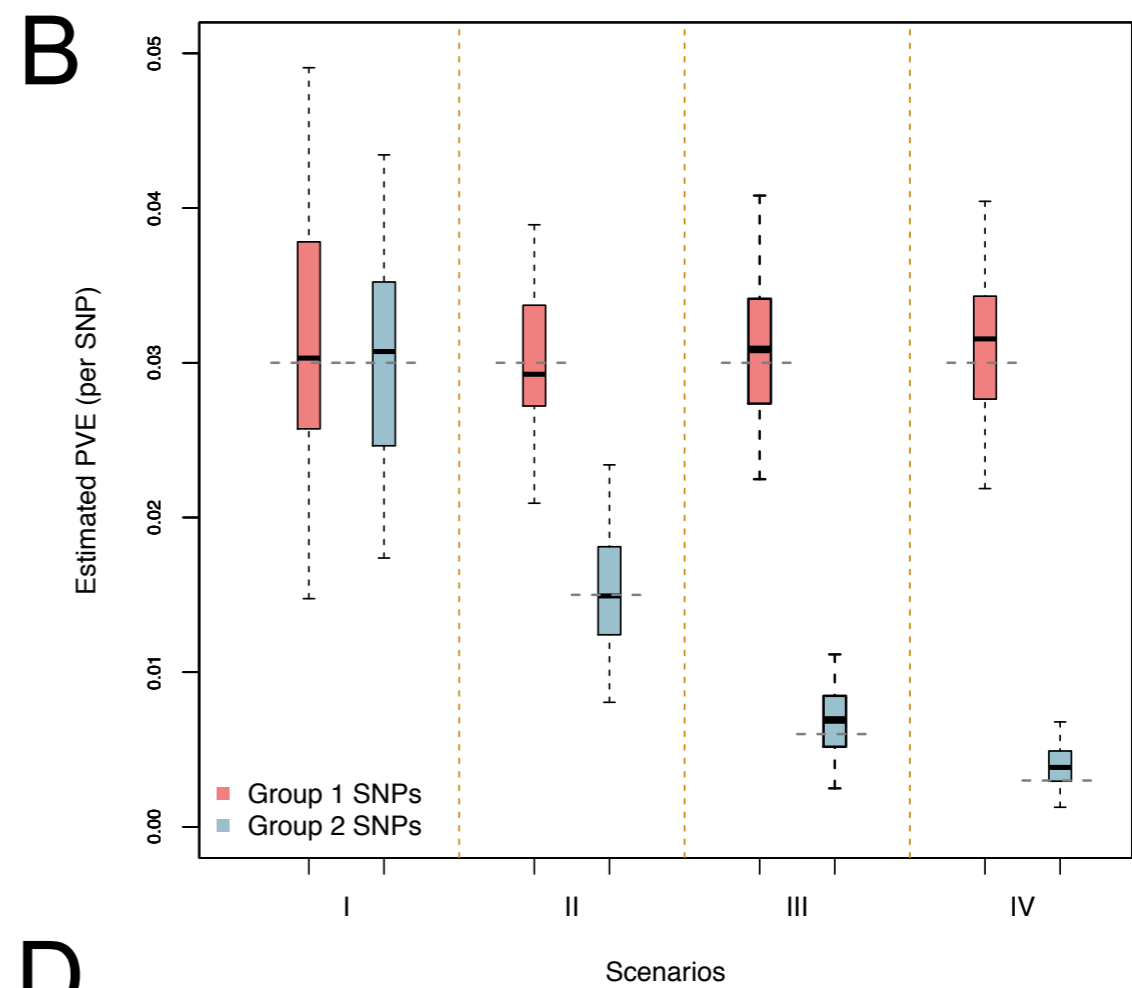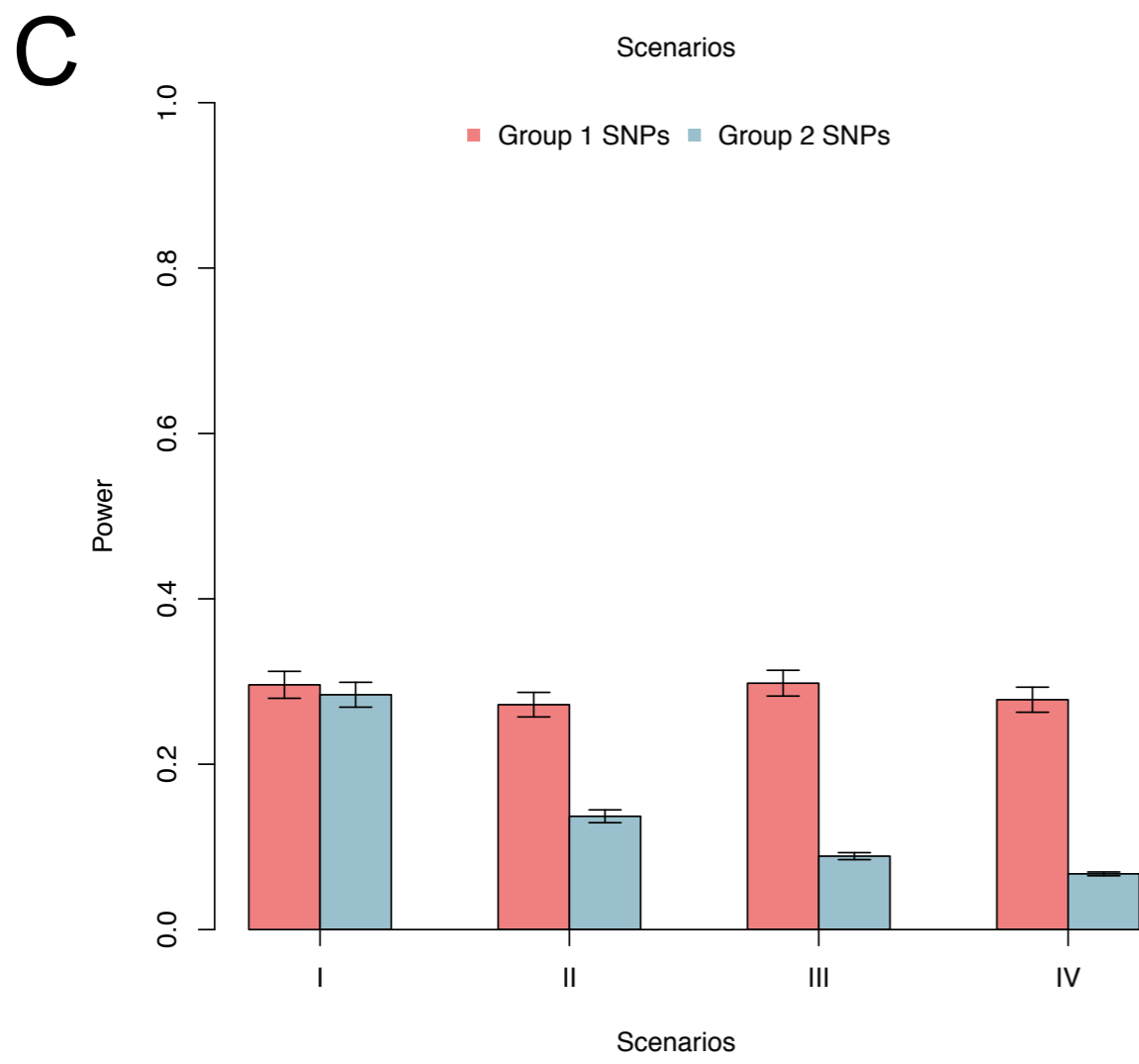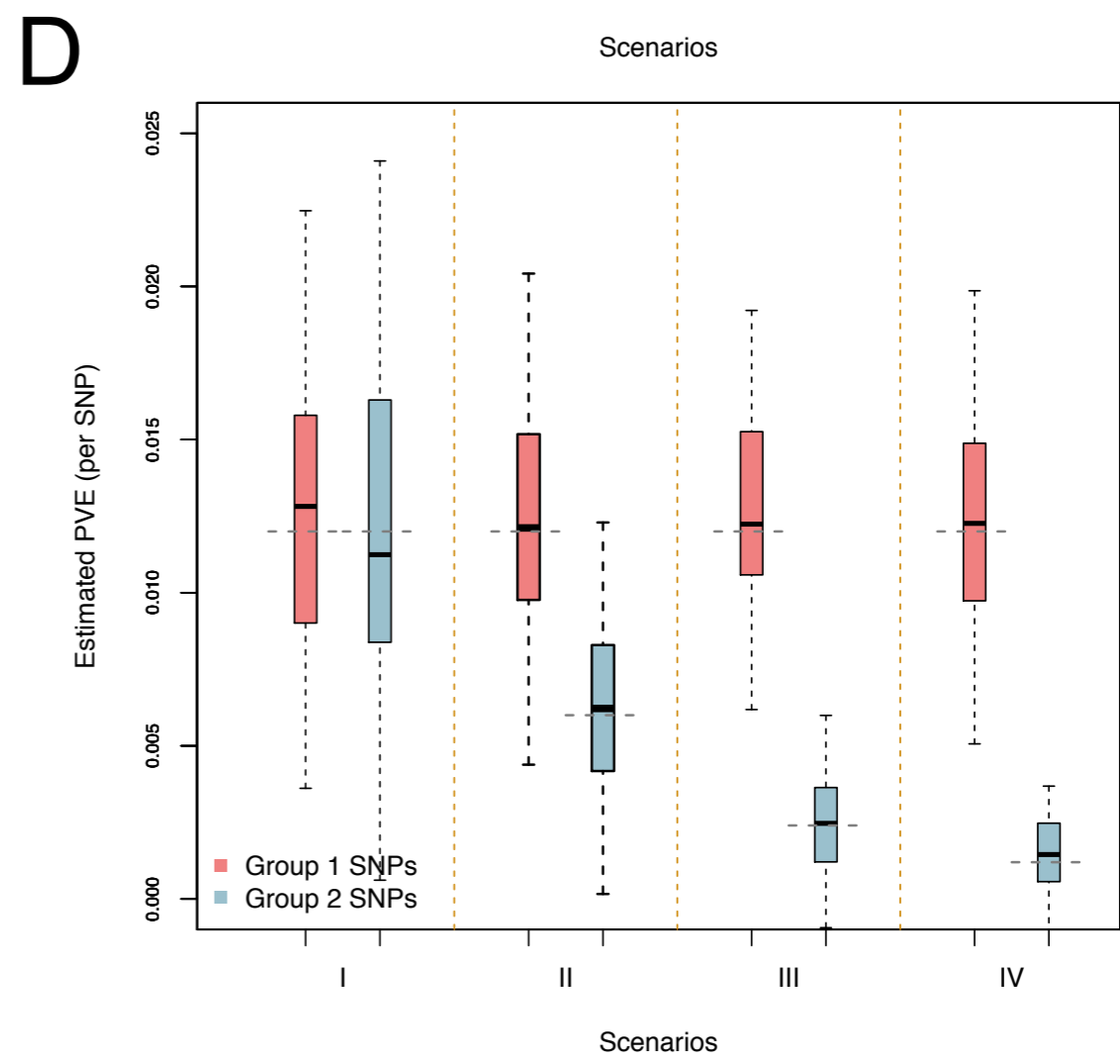

Supplement: S3 Fig — Groups 1 and 2 causal markers are colored in light red and light blue, respectively. These figures are based on a broad-sense heritability level of H2 = 0.6 and parameters ρ = 0.5 (A, B) and ρ = 0.8 (C, D), respectively. These results are estimated with 100 data replicates under simulation model (ii) with the top 5 genotype PCs. Here, ρ = {0.5, 0.8} was used to determine the portion of broad-sense heritability contributed by interaction effects. (A) and (C) show the power of MAPIT to identify SNPs in each causal group under significance level α = 0.05. The lines represent 95% variability due to resampling error. (B) and (D) show boxplots of the marginal PVE estimates for the group 1 and 2 causal SNPs from MAPIT for the four simulation scenarios. The true PVEs per causal SNP are shown as dashed grey horizontal lines. When ρ = 0.05, the true PVEs per causal SNP are: 0.03 for the group 1 SNPs; and 0.03, 0.015, 0.006, and 0.003 for the group 2 SNPs. When ρ = 0.08, the true PVEs per causal SNP are: 0.012 for the group 1 SNPs; and 0.012, 0.006, 0.0024, and 0.0012 for the Group 2 SNPs. (PDF) [file pgen.1006869.s003.pdf]

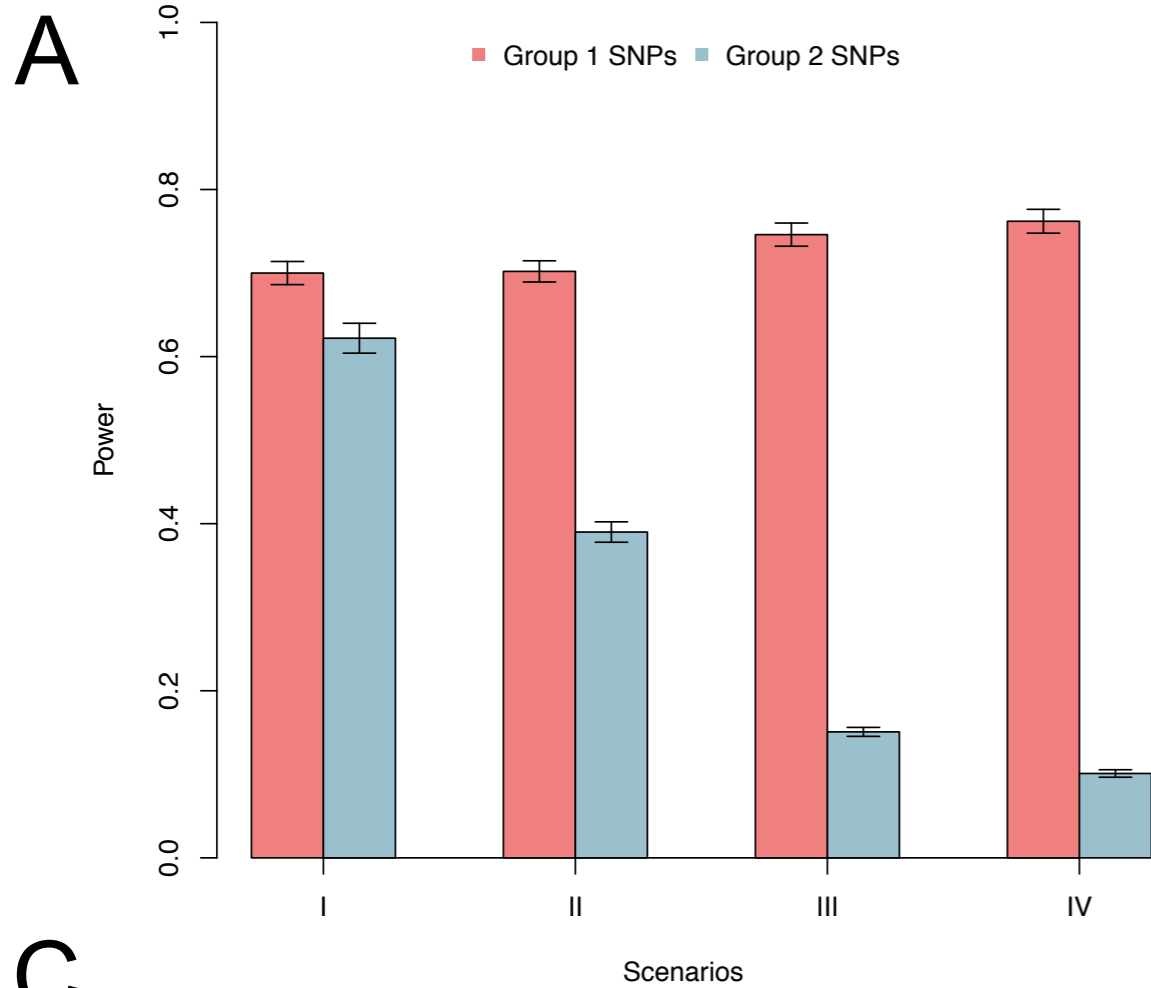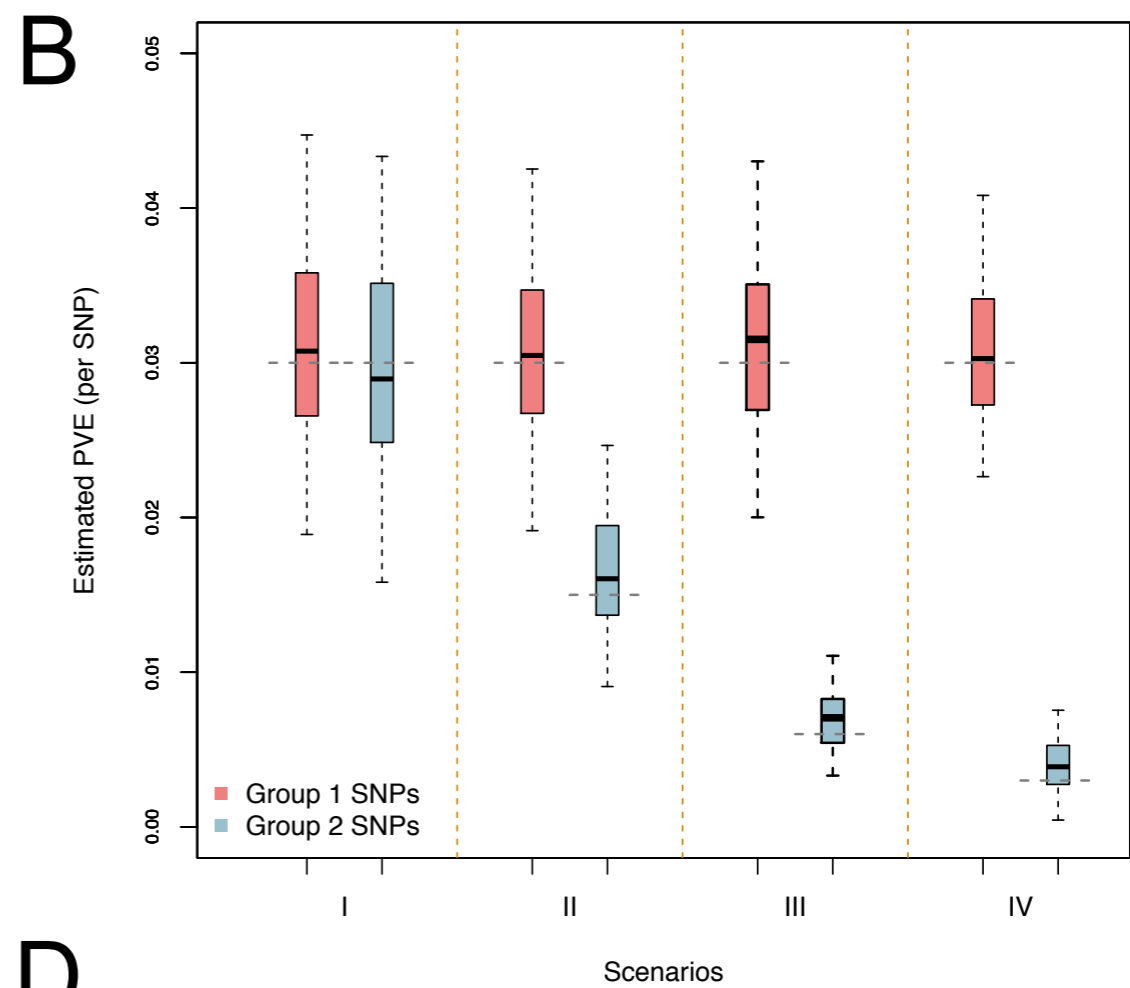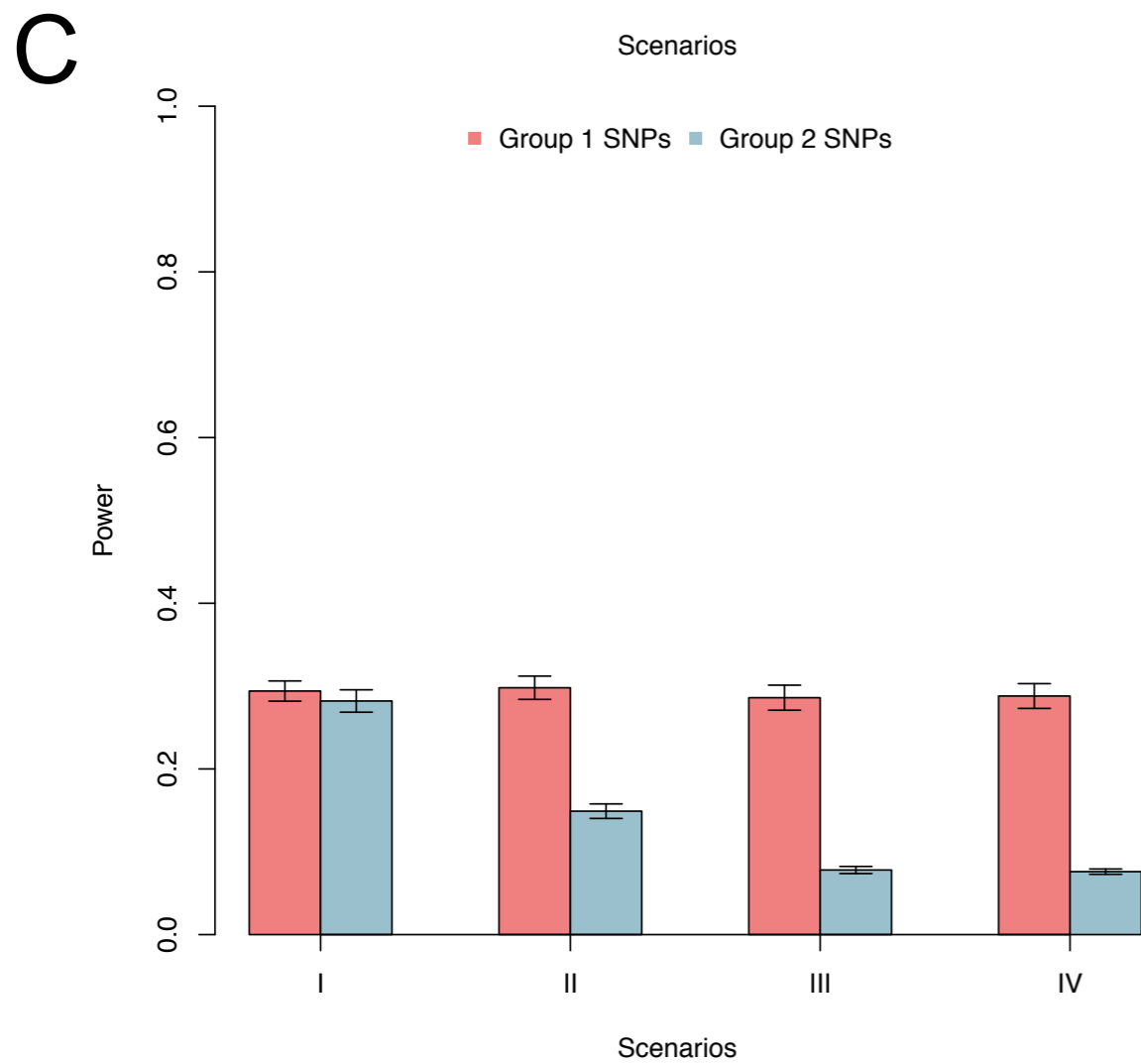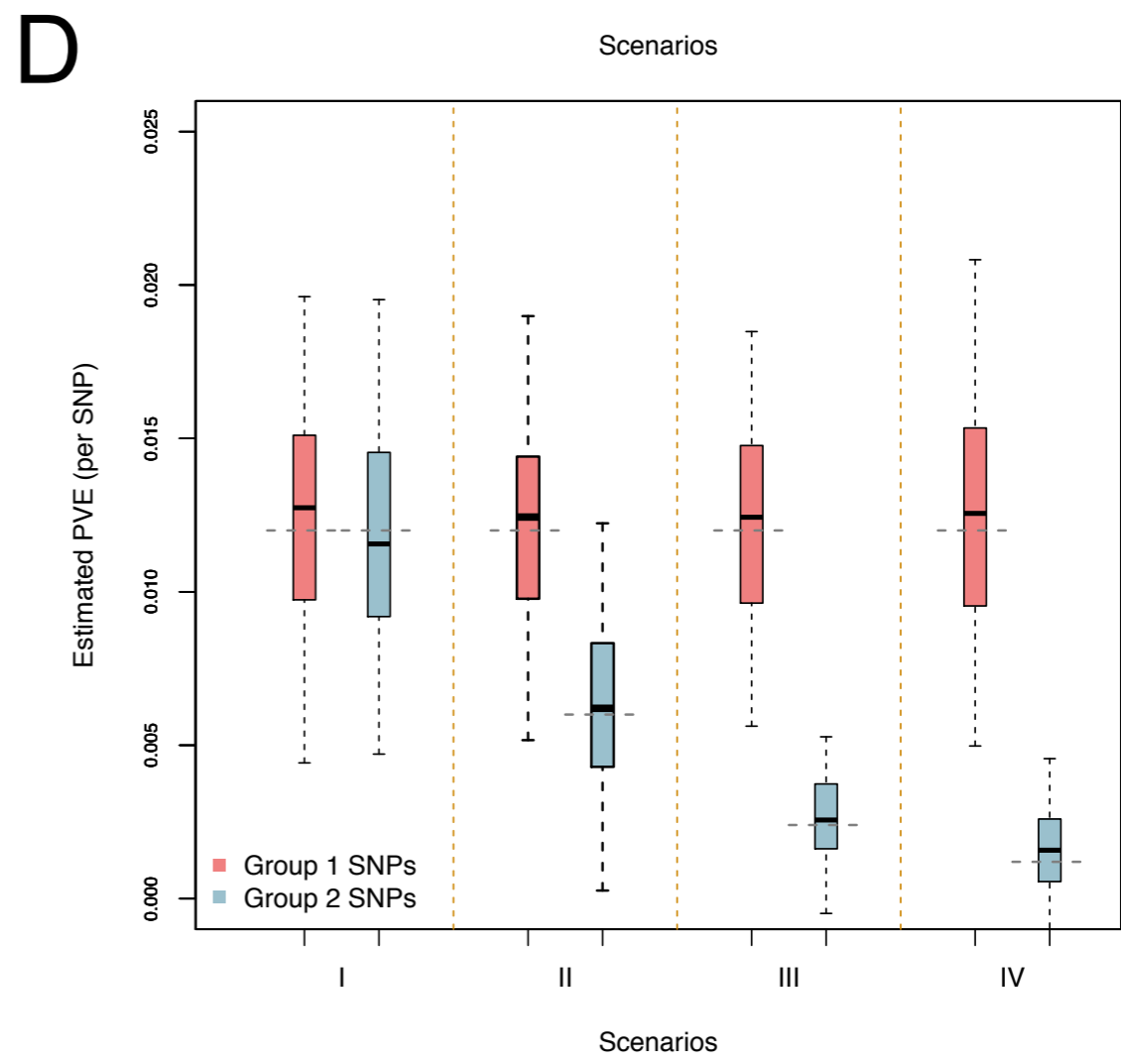

Supplement: S4 Fig — Groups 1 and 2 causal markers are colored in light red and light blue, respectively. These figures are based on a broad-sense heritability level of H2 = 0.6 and parameters ρ = 0.5 (A, B) and ρ = 0.8 (C, D), respectively. These results are estimated with 100 data replicates under simulation model (ii) with the top 10 genotype PCs. Here, ρ = {0.5, 0.8} was used to determine the portion of broad-sense heritability contributed by interaction effects. (A) and (C) show the power of MAPIT to identify SNPs in each causal group under significance level α = 0.05. The lines represent 95% variability due to resampling error. (B) and (D) show boxplots of the marginal PVE estimates for the group 1 and 2 causal SNPs from MAPIT for the four simulation scenarios. The true PVEs per causal SNP are shown as dashed grey horizontal lines. When ρ = 0.05, the true PVEs per causal SNP are: 0.03 for the group 1 SNPs; and 0.03, 0.015, 0.006, and 0.003 for the group 2 SNPs. When ρ = 0.08, the true PVEs per causal SNP are: 0.012 for the group 1 SNPs; and 0.012, 0.006, 0.0024, and 0.0012 for the Group 2 SNPs. (PDF) [file pgen.1006869.s004.pdf]

A

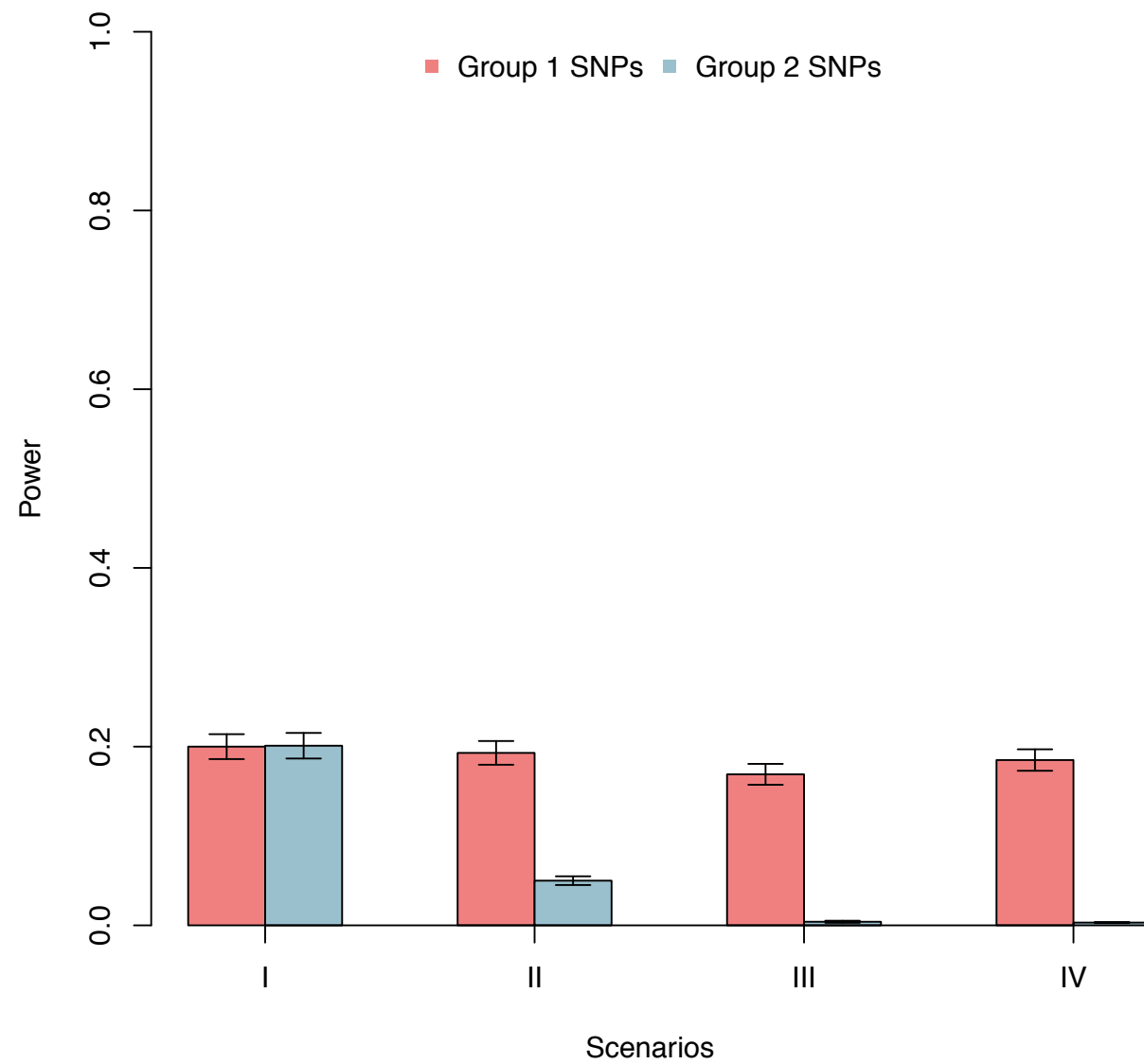

B

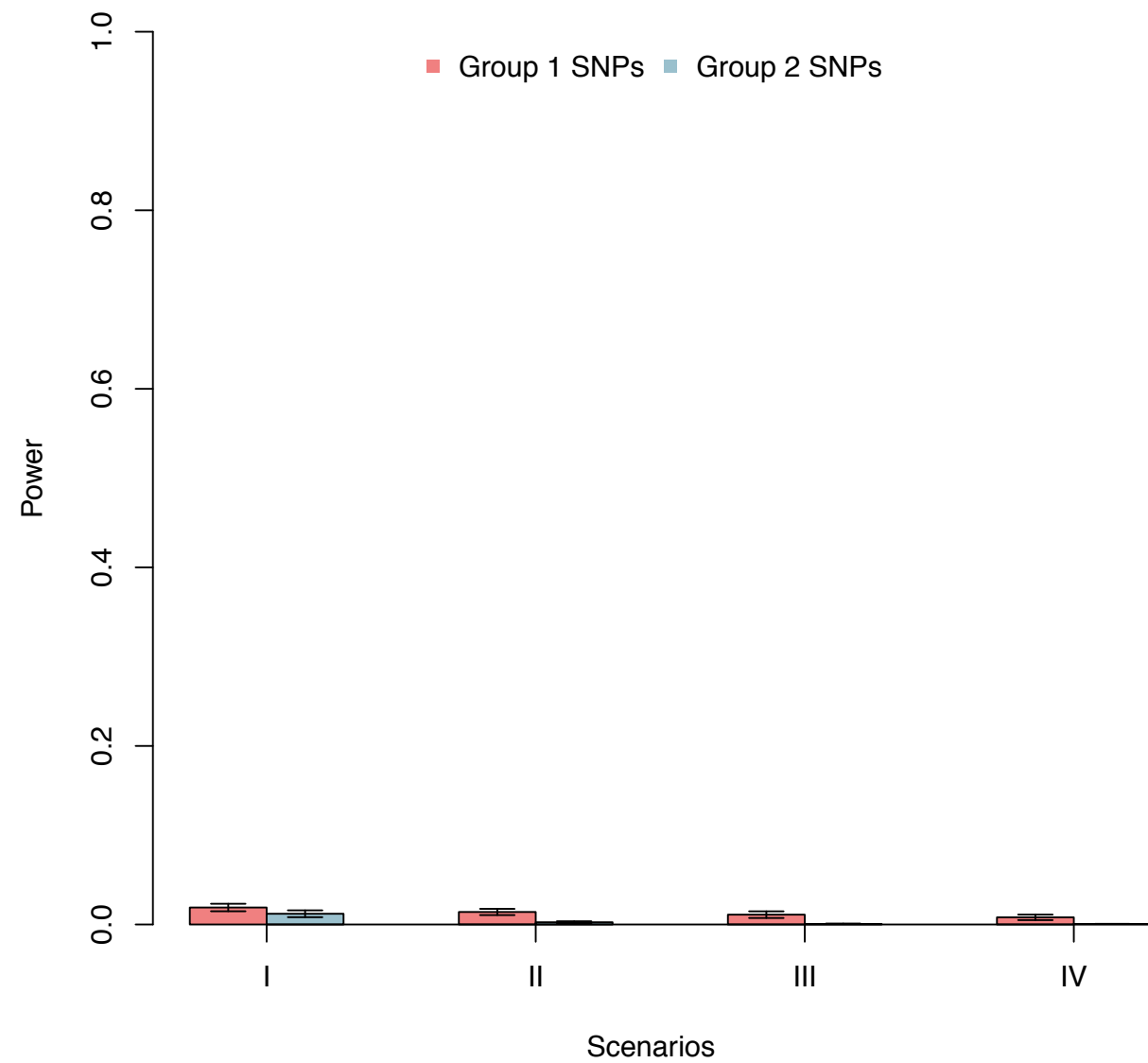

Supplement: S5 Fig — (A) and (B) show the power of MAPIT to identify SNPs in each causal group under the Bonferroni-corrected genome-wide significance level α = 8.3 × 10^−6^. Groups 1 and 2 causal markers are colored in light red and light blue, respectively. These figures are based on a broad-sense heritability level of H2 = 0.6, and parameters ρ = 0.5 (A) and ρ = 0.8 (B)—estimated with 100 replicates. Here, ρ was used to determine the portion of broad-sense heritability contributed by interaction effects. The lines represent 95% variability due to resampling error. (PDF) [file pgen.1006869.s005.pdf]

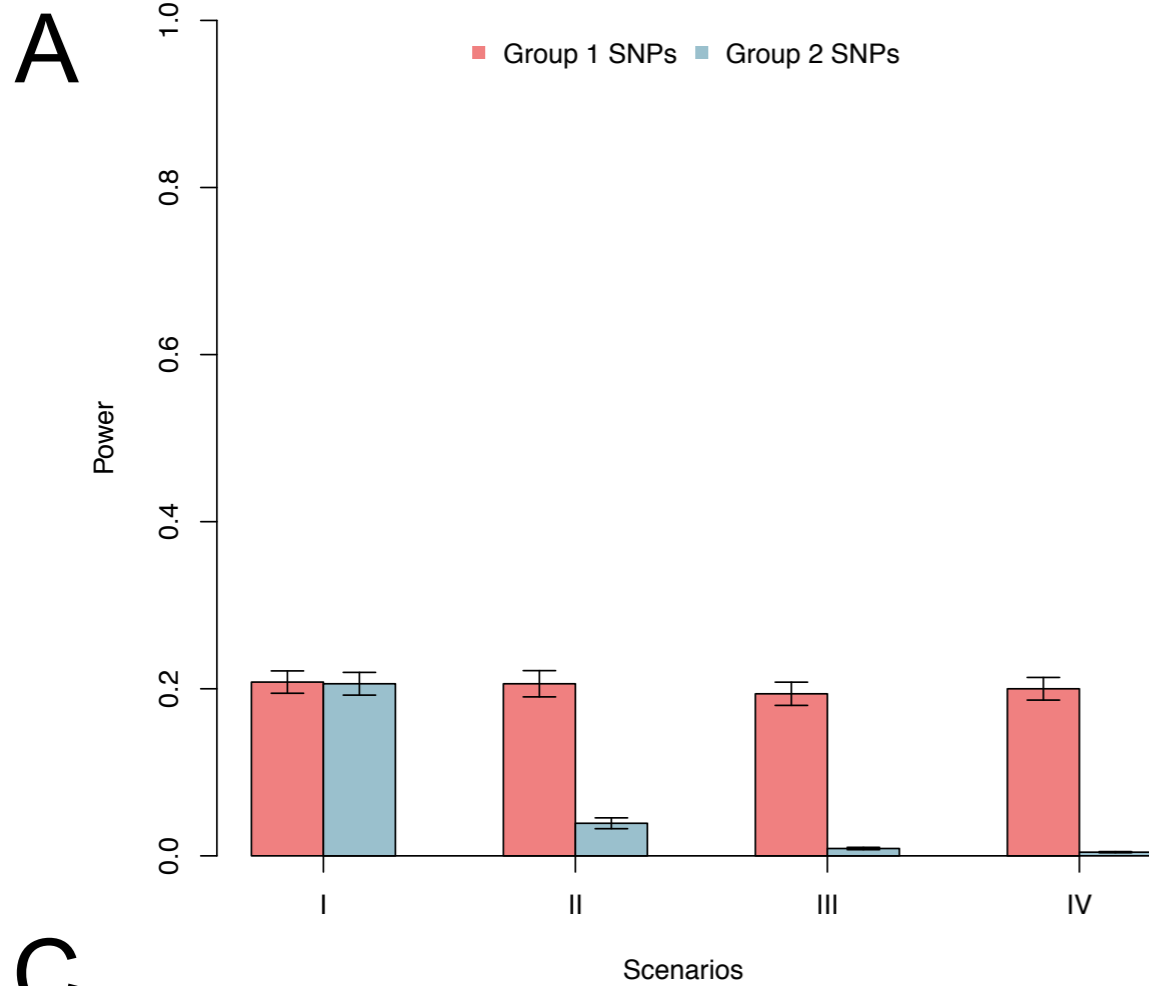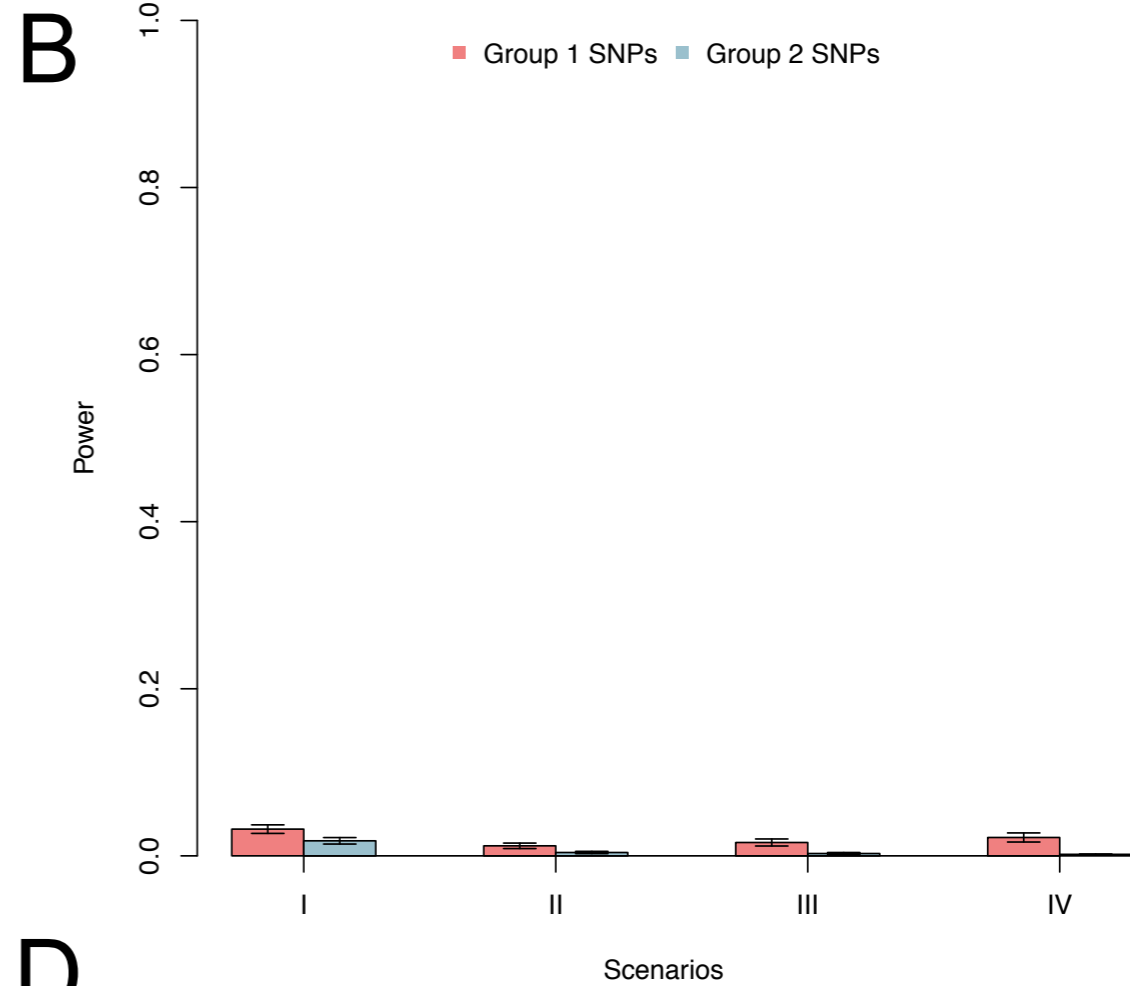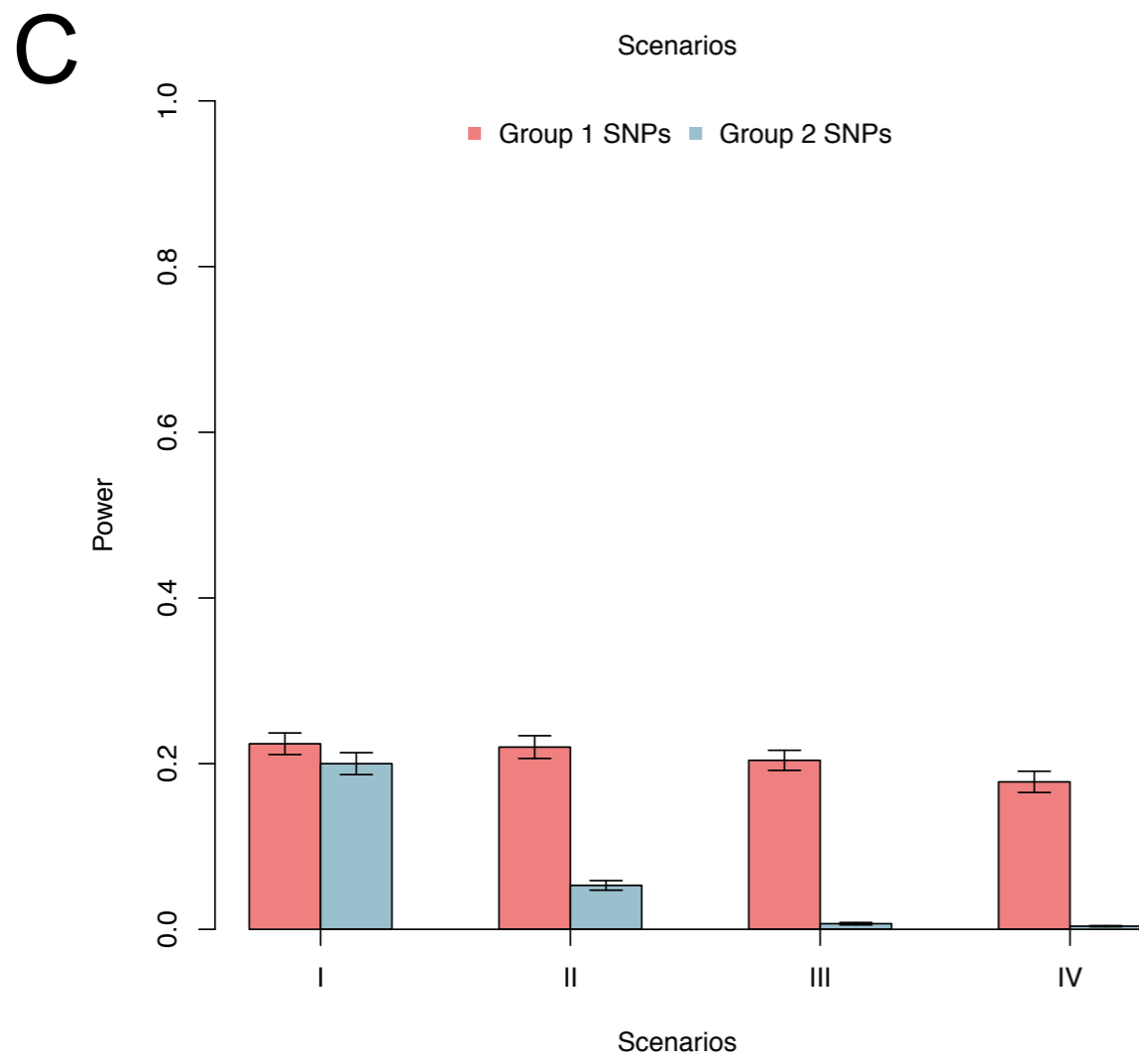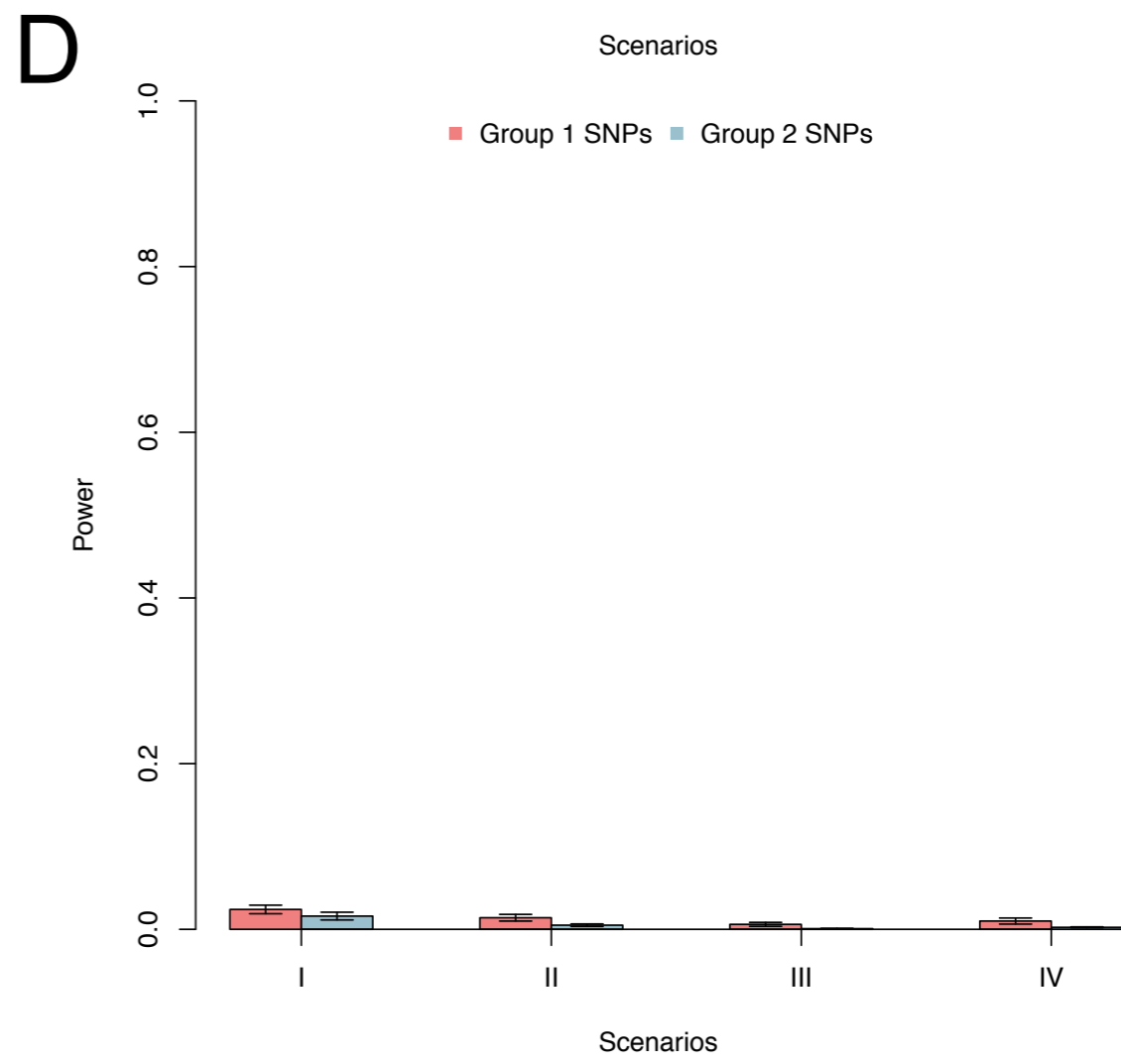

Supplement: S6 Fig — All Figures show the power of MAPIT to identify SNPs in each causal group under the Bonferroni-corrected genome-wide significance level α = 8.3 × 10^−6^. These results are estimated with 100 data replicates under simulation model (ii). (A) and (B) use the top 5 genotype PCs. (C) and (D) use the top 10 genotype PCs. Groups 1 and 2 causal markers are colored in light red and light blue, respectively. These figures are based on a broad-sense heritability level of H2 = 0.6, and parameters ρ = 0.5 (A, C) and ρ = 0.8 (B, D). Here, ρ was used to determine the portion of broad-sense heritability contributed by interaction effects. The lines represent 95% variability due to resampling error. (PDF) [file pgen.1006869.s006.pdf]

A

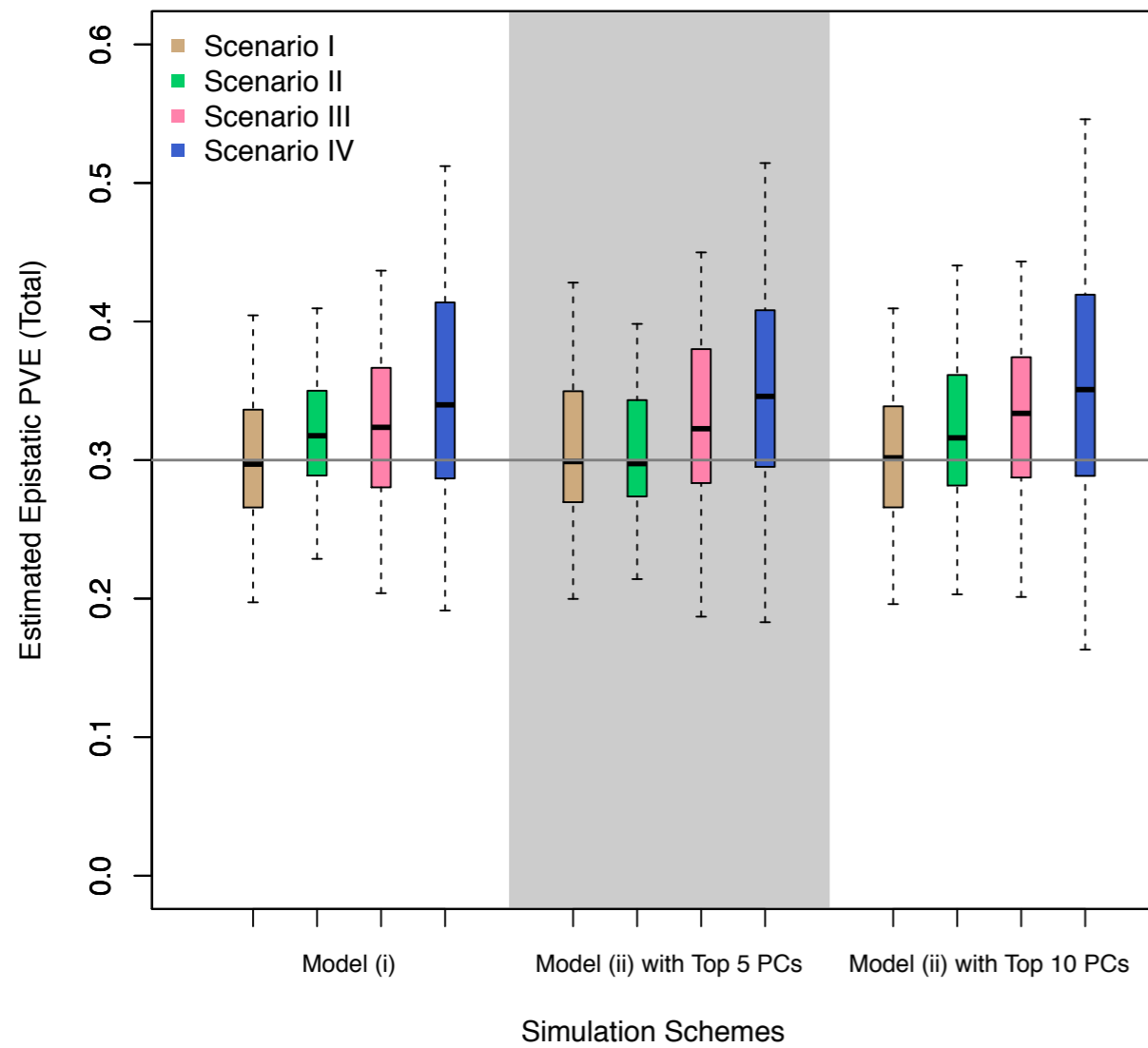

B

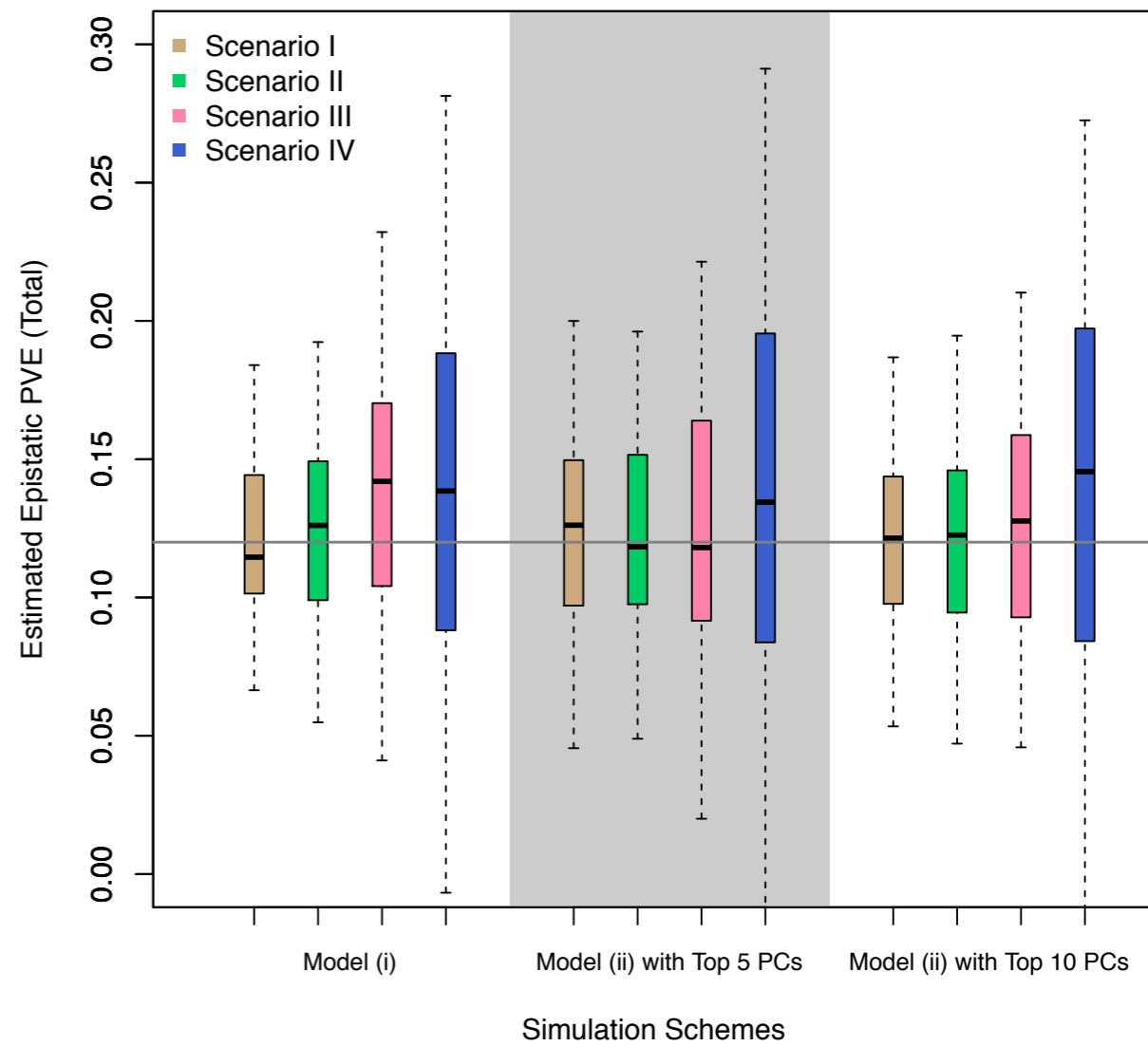

Supplement: S7 Fig — Compared here are the epistatic PVE estimates computed by the standard variance component model. Each simulation scenario is represented by a different color, with each of the three simulation schemes being labeled on the x-axis. These figures are based on 100 simulations where the overall broad-sense heritability level is H2 = 0.6, and the parameters ρ = 0.5 (A) and ρ = 0.8 (B). Here, ρ was used to determine the portion of broad-sense heritability contributed by interaction effects. In (A), the true epistatic PVE is 0.3. In (B), the true epistatic PVE is 0.12. In both cases, the true PVE is shown as the grey horizontal line. (PDF) [file pgen.1006869.s007.pdf]

A

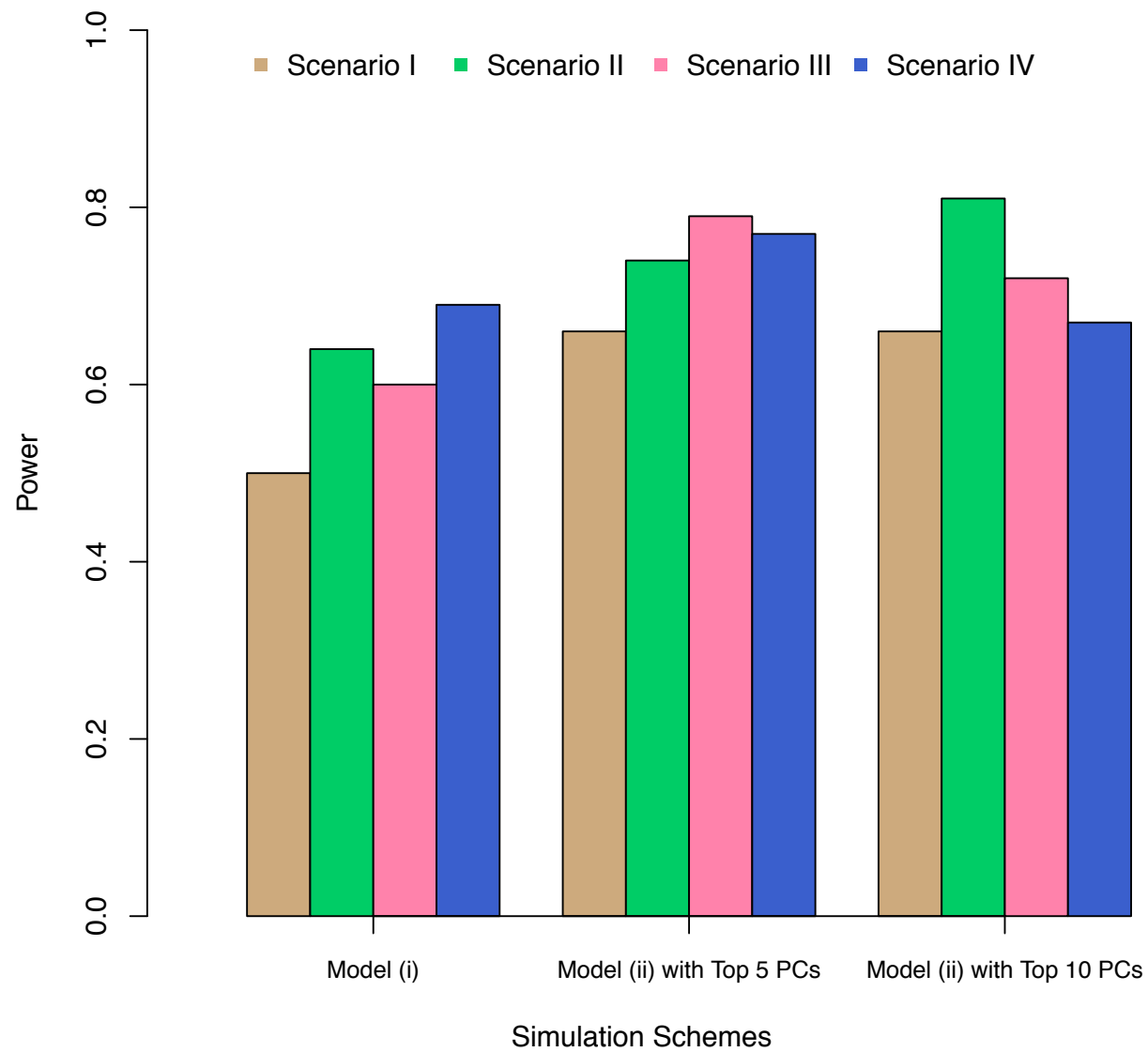

B

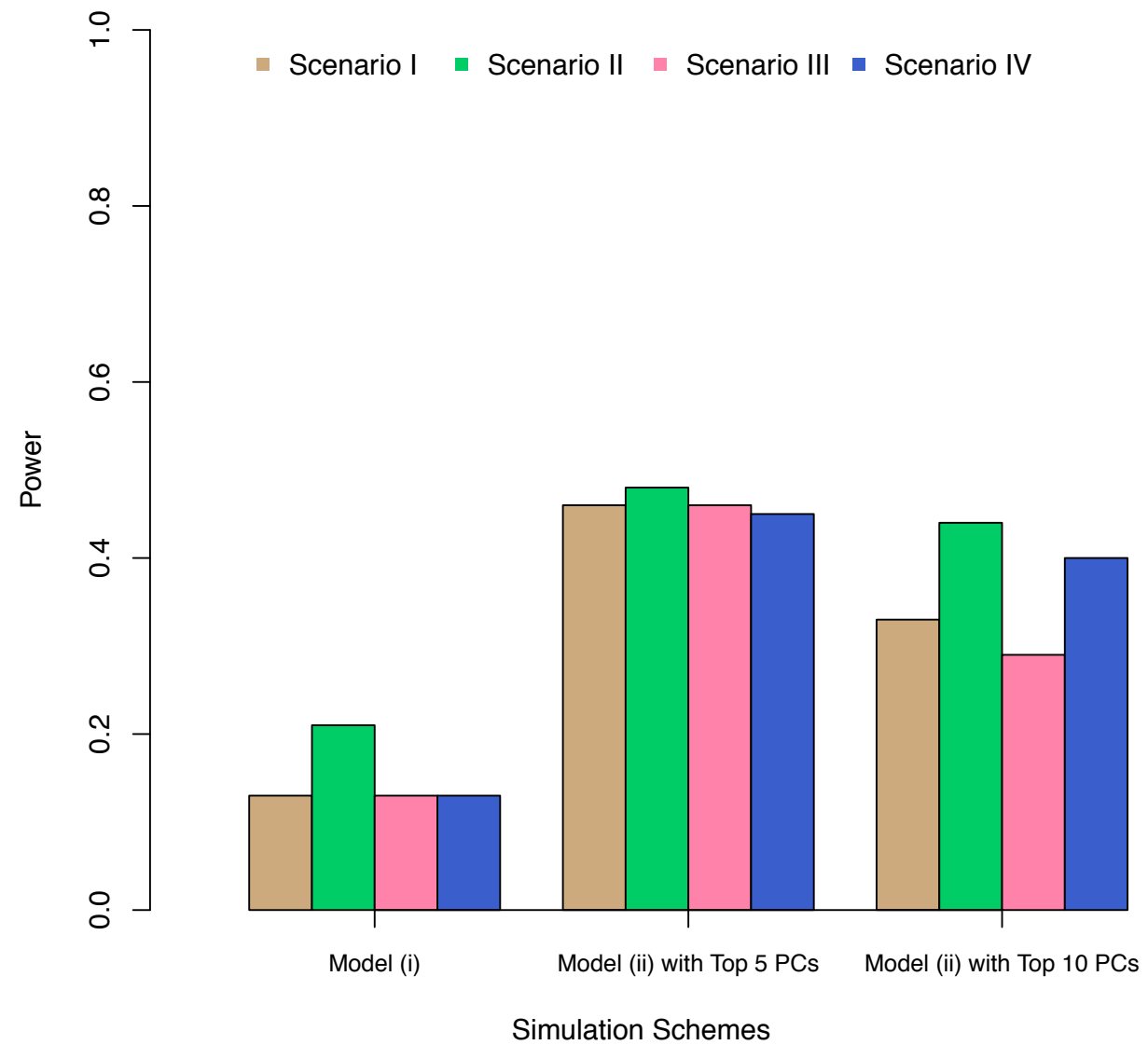

Supplement: S8 Fig — Compared here is the power of the standard variance component model to estimate the true non-zero pairwise epistatic PVE at the significance level of α = 0.05 under a standard asymptotic normal test. Each simulation scenario is represented by a different color, with each of the three simulation schemes being labeled on the x-axis. These figures are based on 100 simulations where the overall broad-sense heritability level is H2 = 0.6, and the parameters ρ = 0.5 (A) and ρ = 0.8 (B). Here, ρ was used to determine the portion of broad-sense heritability contributed by interaction effects. (PDF) [file pgen.1006869.s008.pdf]

**A**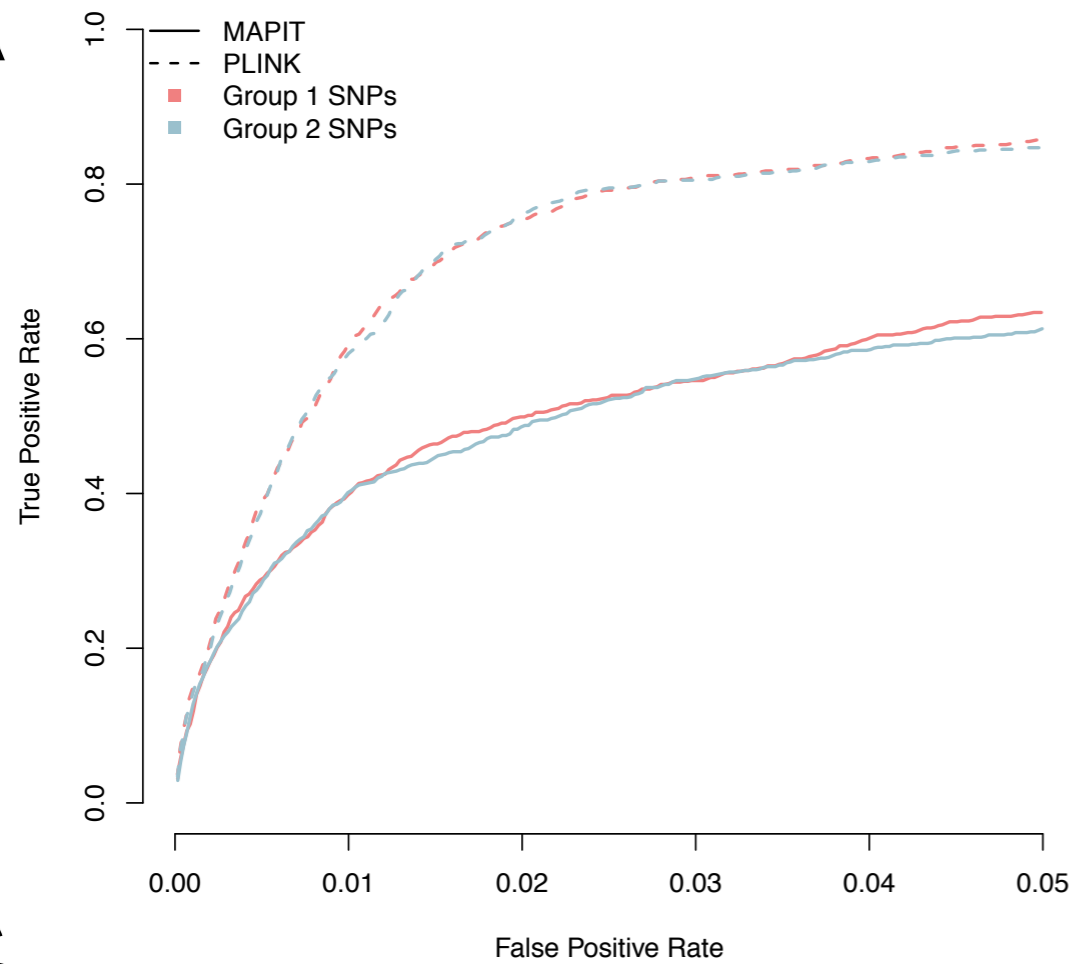**B**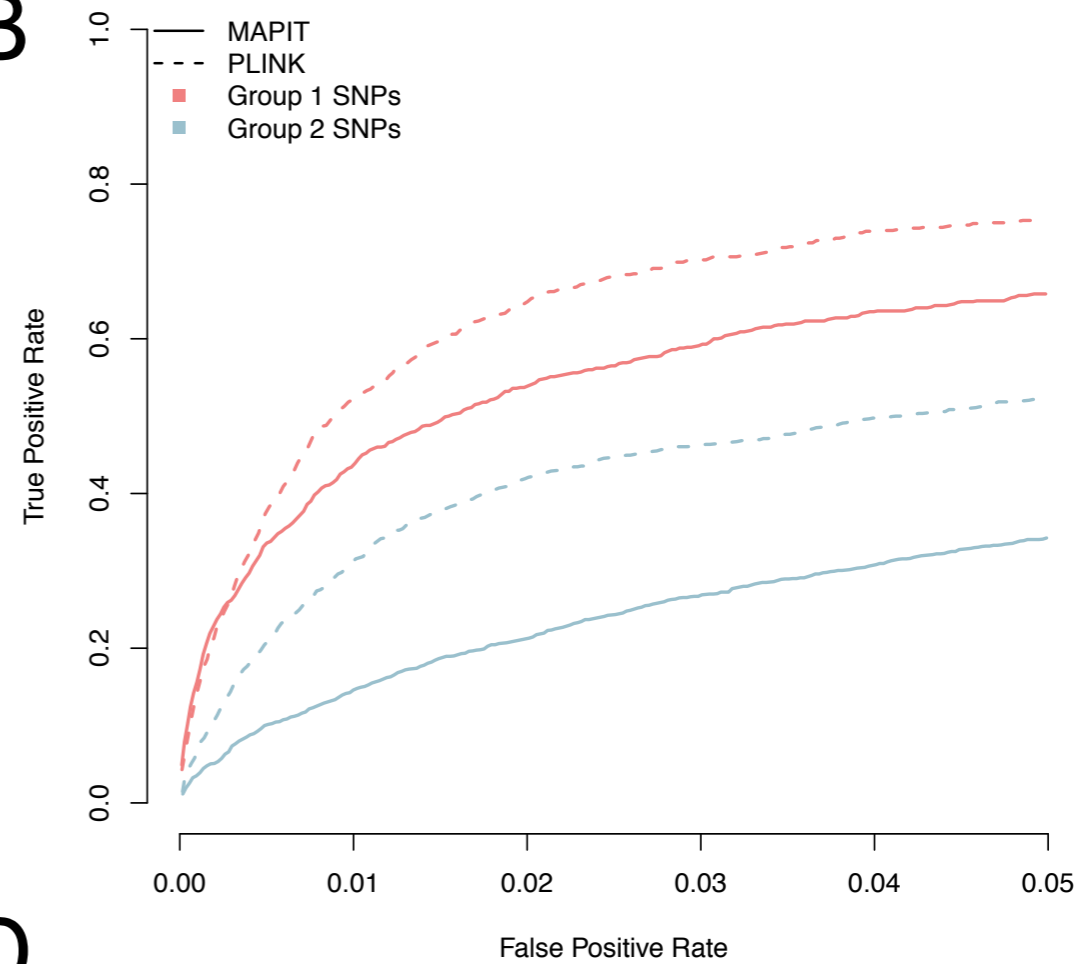**C**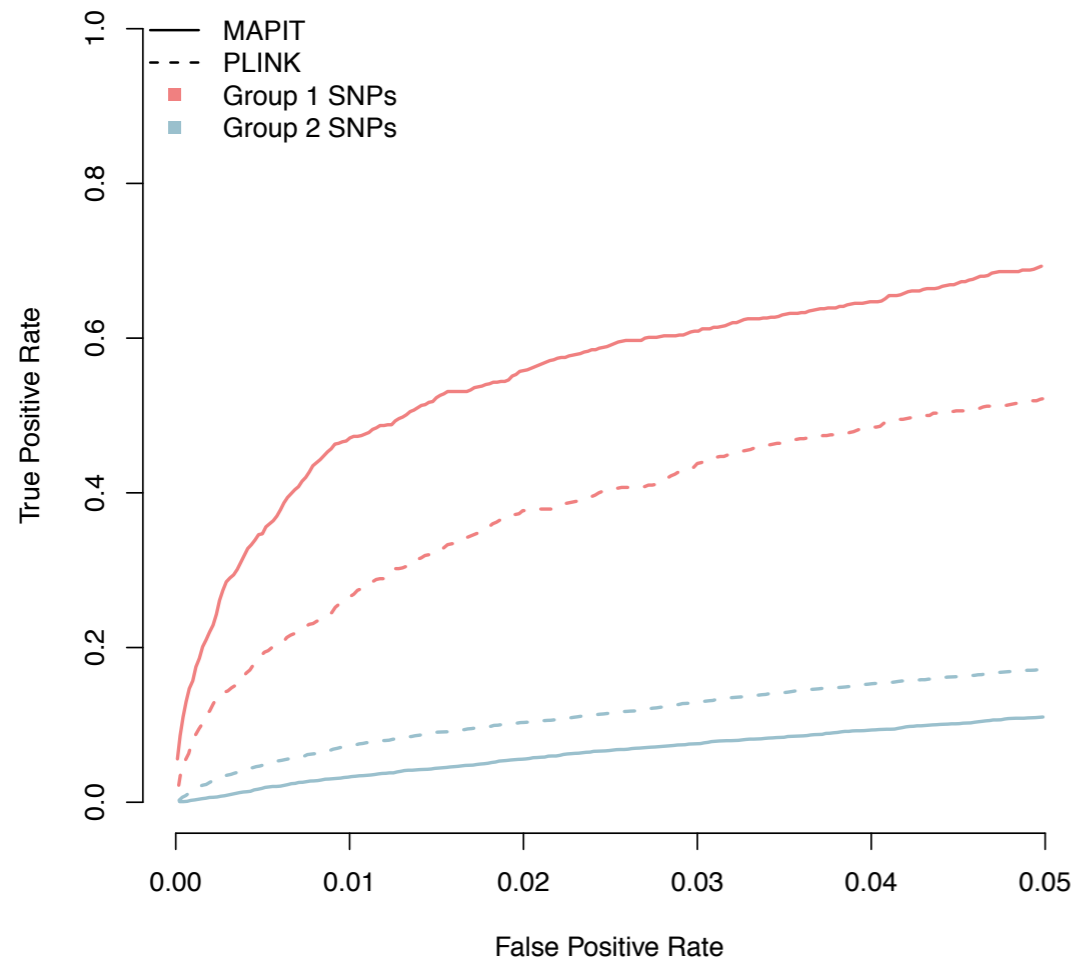**D**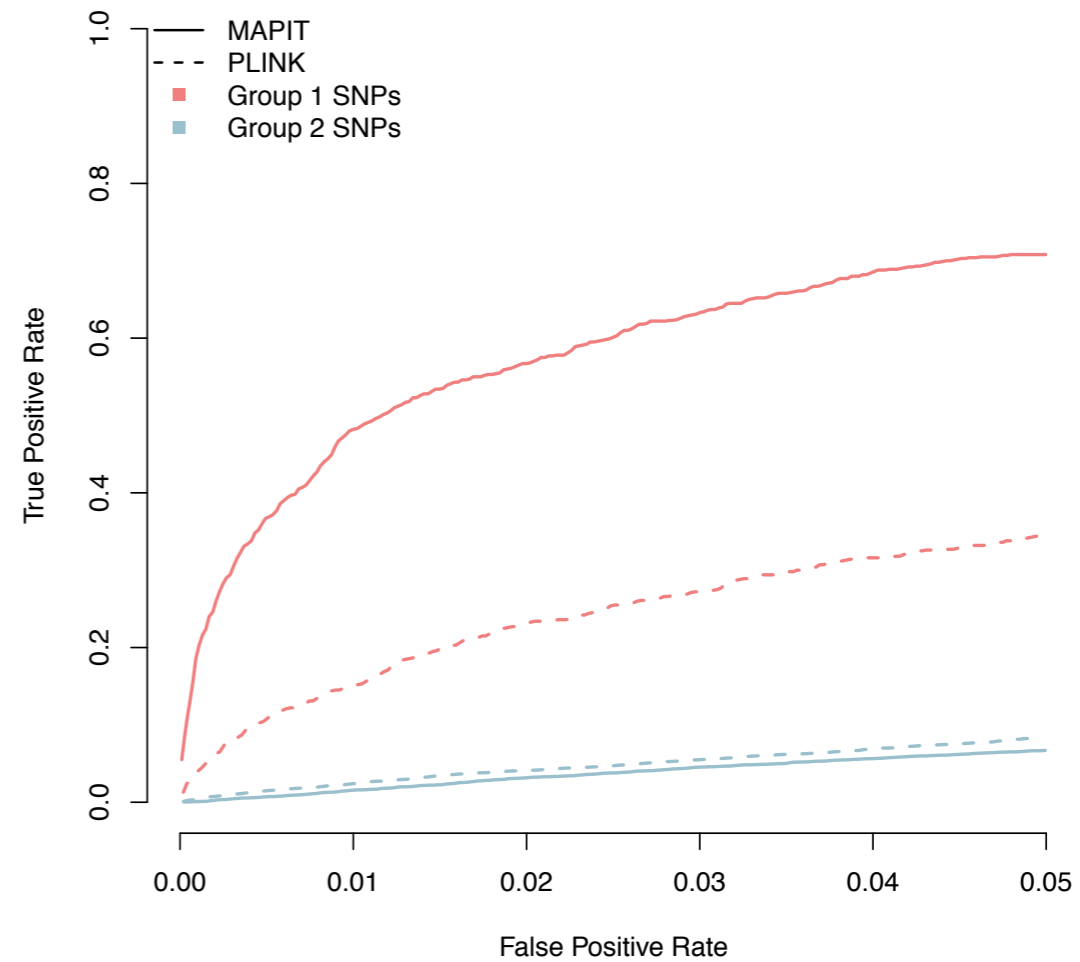

Supplement: S9 Fig — We compare the mapping abilities of MAPIT (solid line) to the exhaustive search procedure in PLINK (dotted line) in scenarios I (A), II (B), III (C), and IV (D), under broad-sense heritability level H2 = 0.6 and ρ = 0.5. Here, ρ = 0.5 was used to determine the portion of broad-sense heritability contributed by interaction effects. Group 1 (light red) and group 2 (light blue) causal SNPs. The x-axis shows the false positive rate, while the y-axis gives the rate at which true causal variants were identified. Results are based on 100 replicates in each case. (PDF) [file pgen.1006869.s009.pdf]

**A**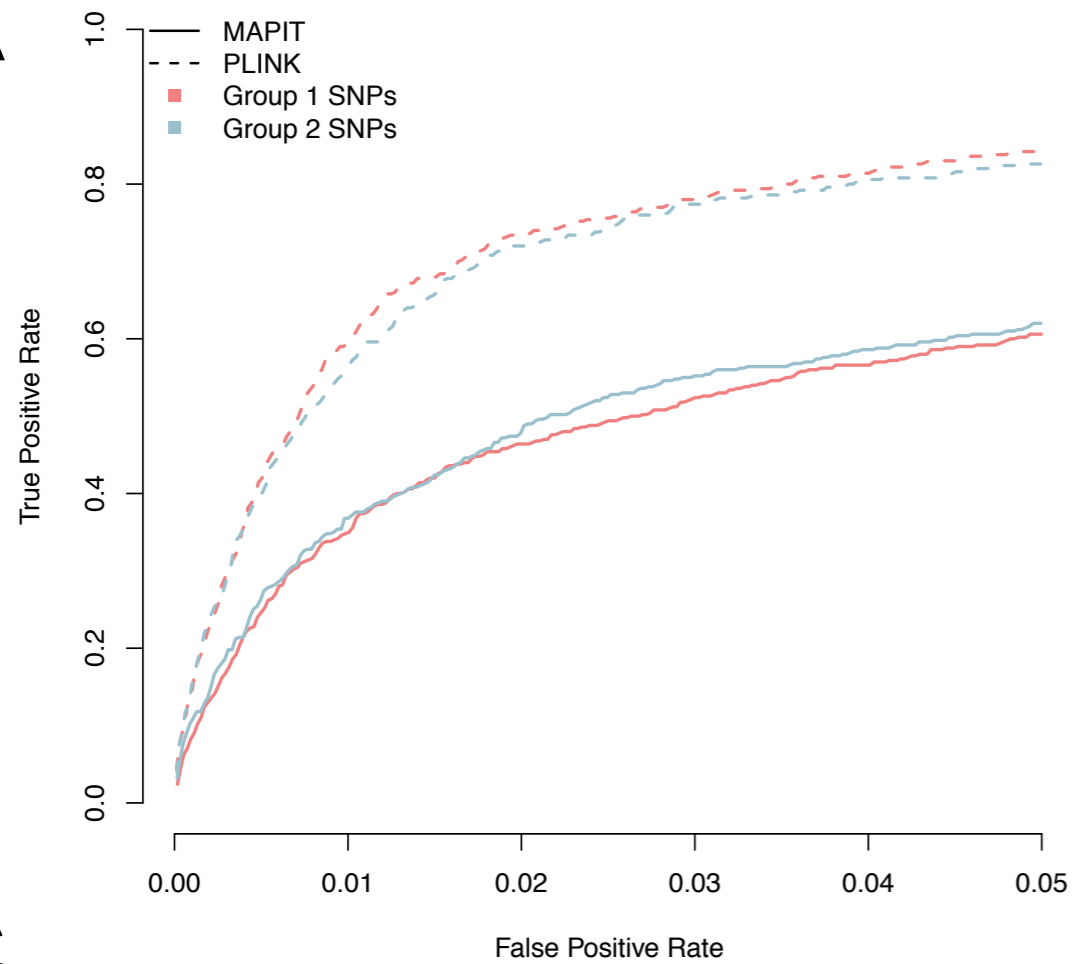**B**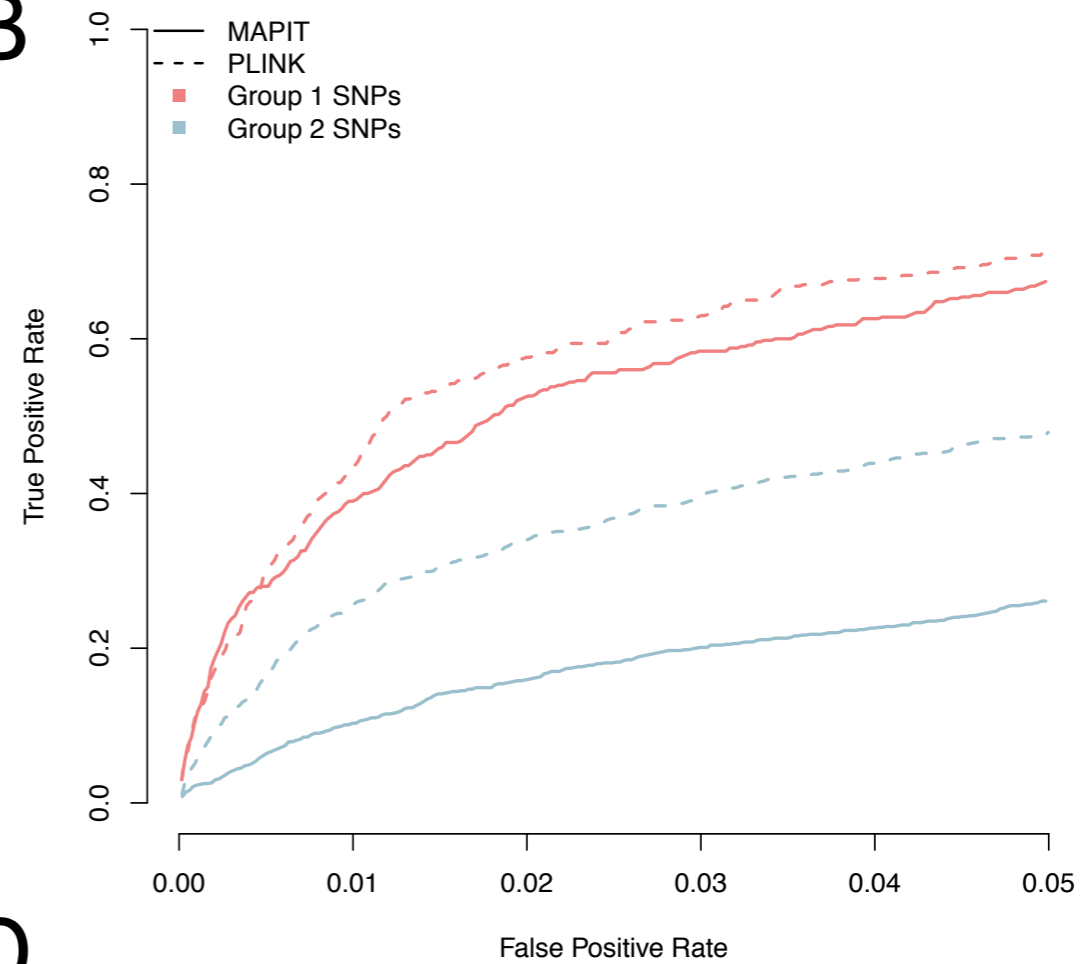**C**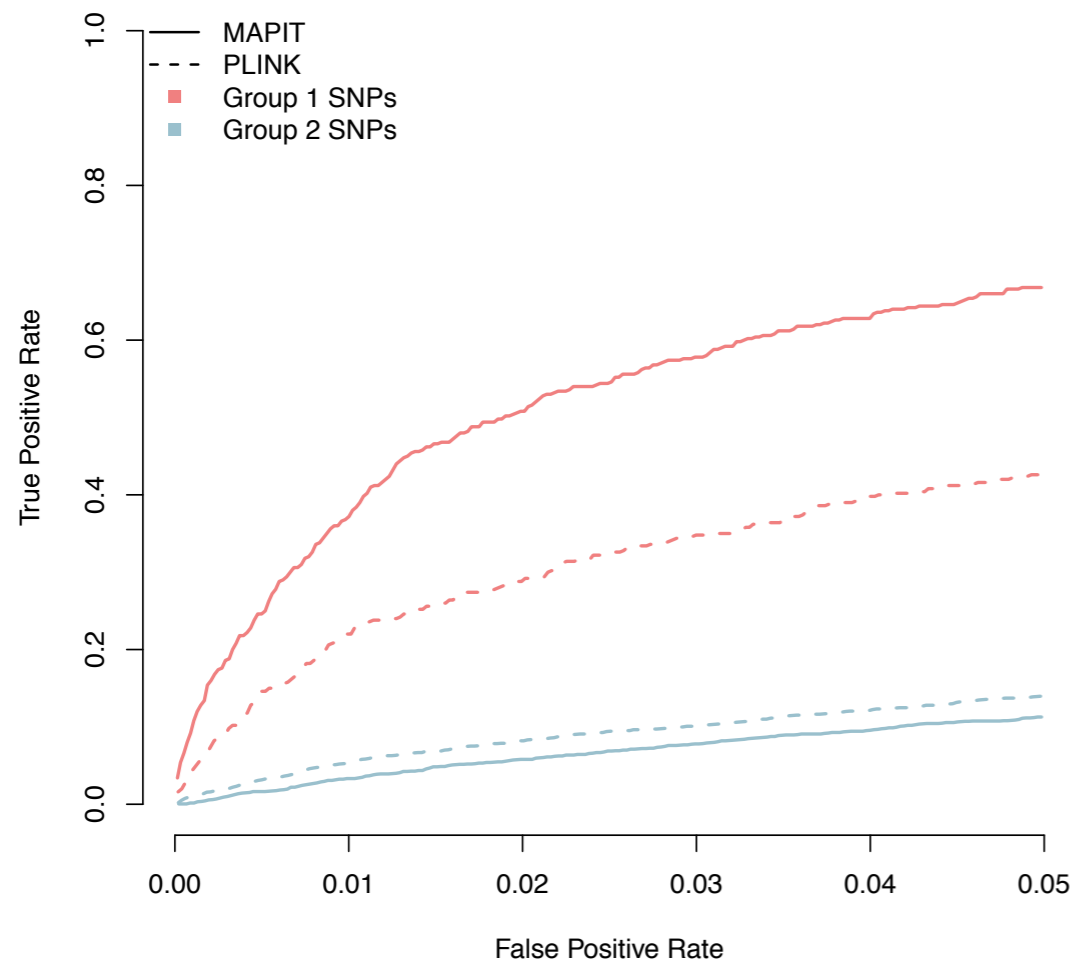**D**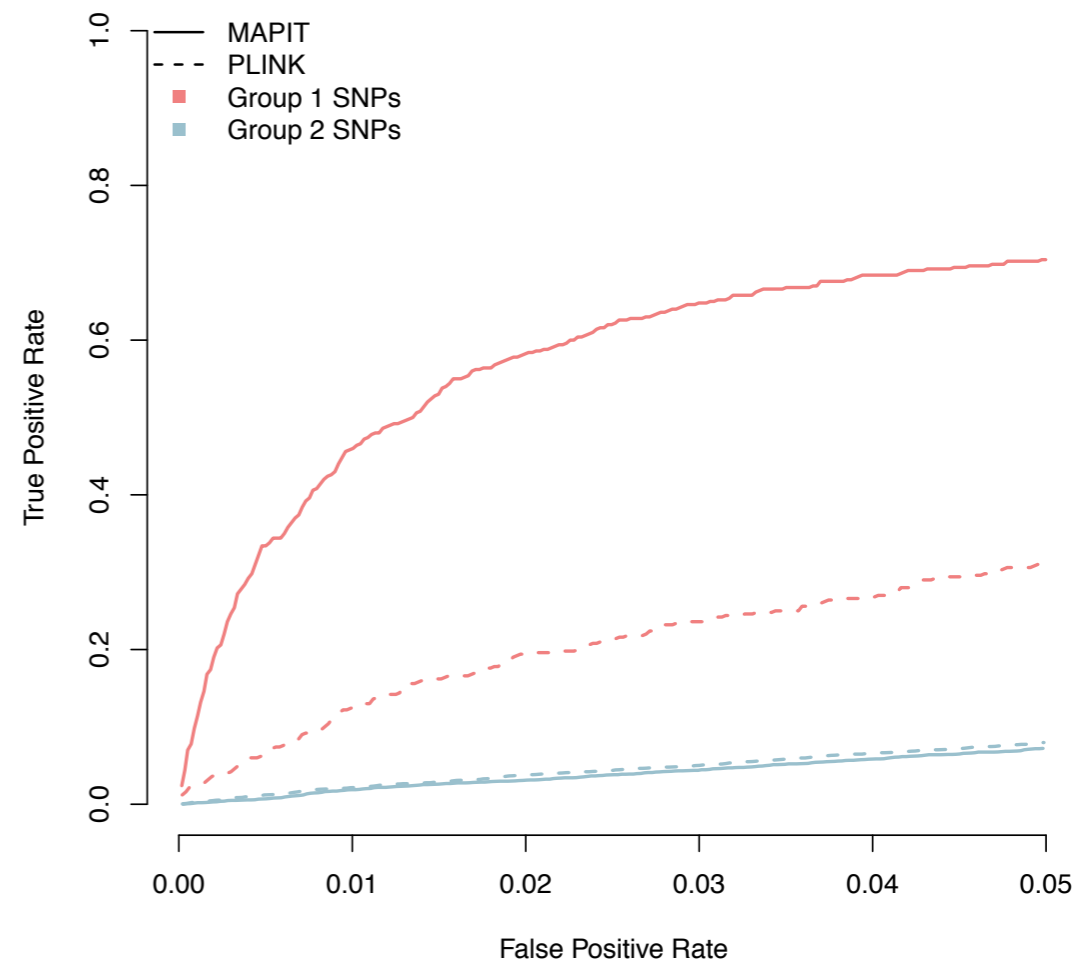

Supplement: S10 Fig — We compare the mapping abilities of MAPIT (solid line) to the exhaustive search procedure in PLINK (dotted line) in scenarios I (A), II (B), III (C), and IV (D), under broad-sense heritability level H2 = 0.6 and ρ = 0.5. Here, ρ = 0.5 was used to determine the portion of broad-sense heritability contributed by interaction effects. Group 1 (light red) and group 2 (light blue) causal SNPs. The x-axis shows the false positive rate, while the y-axis gives the rate at which true causal variants were identified. Results are based on 100 replicates in each case, where the data was created under simulation model (ii) with the top 5 genotype PCs. (PDF) [file pgen.1006869.s010.pdf]

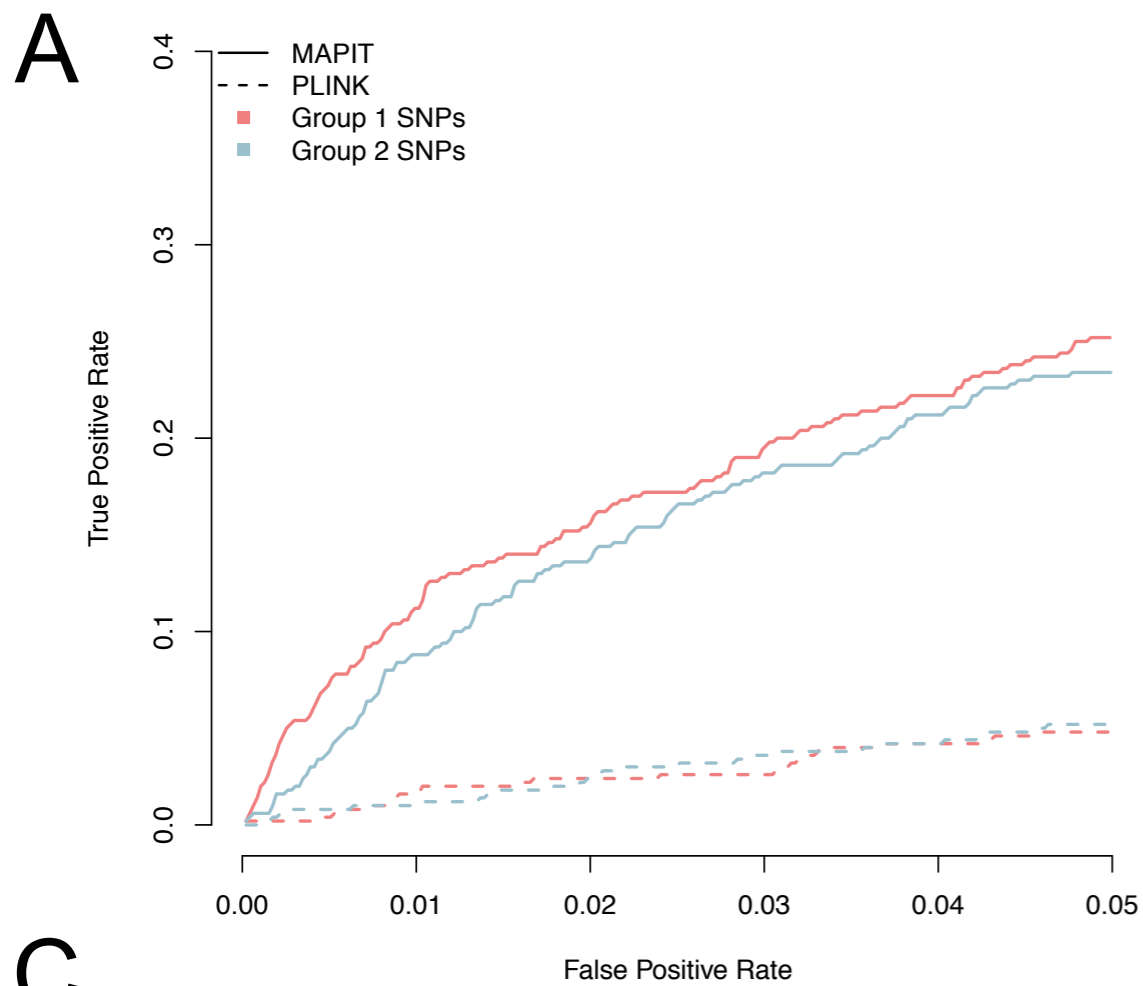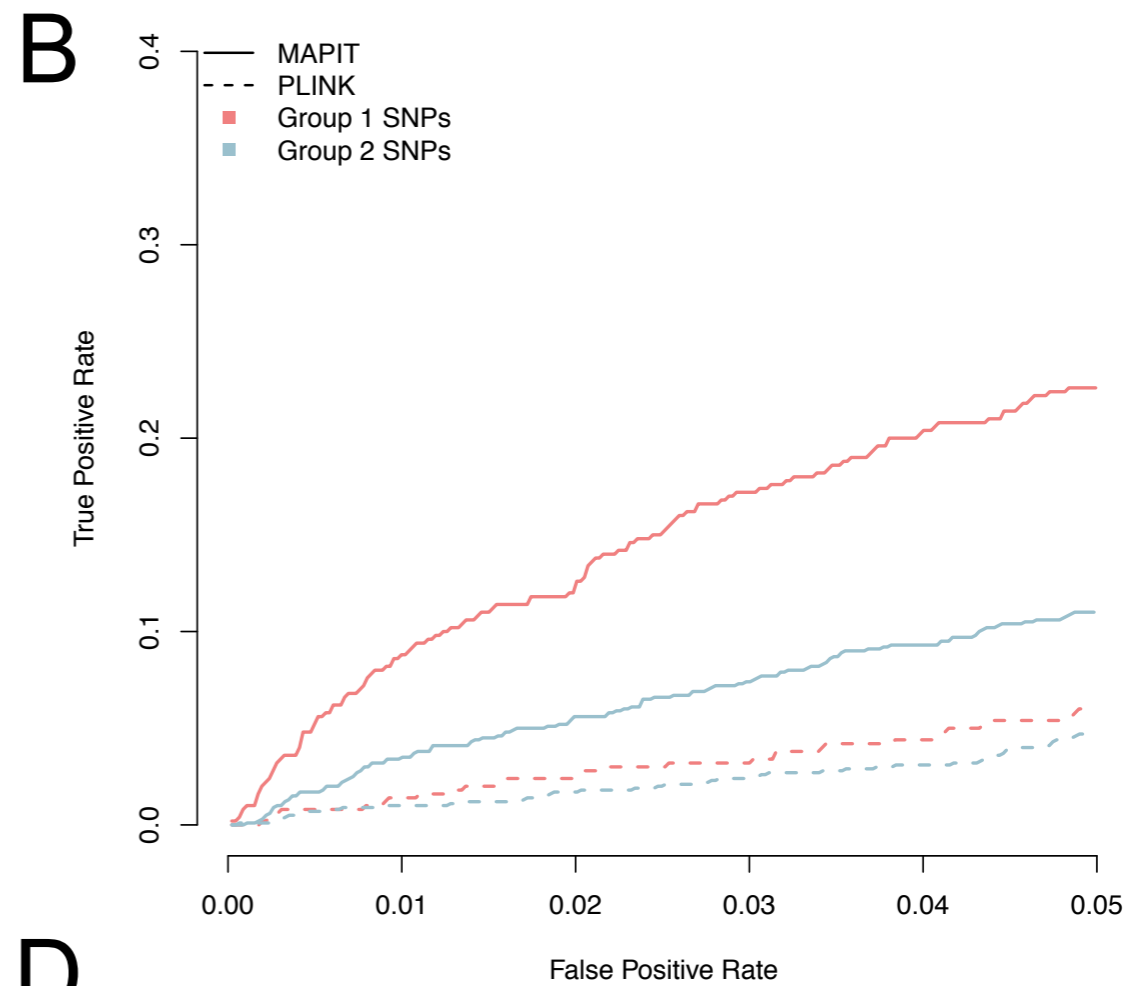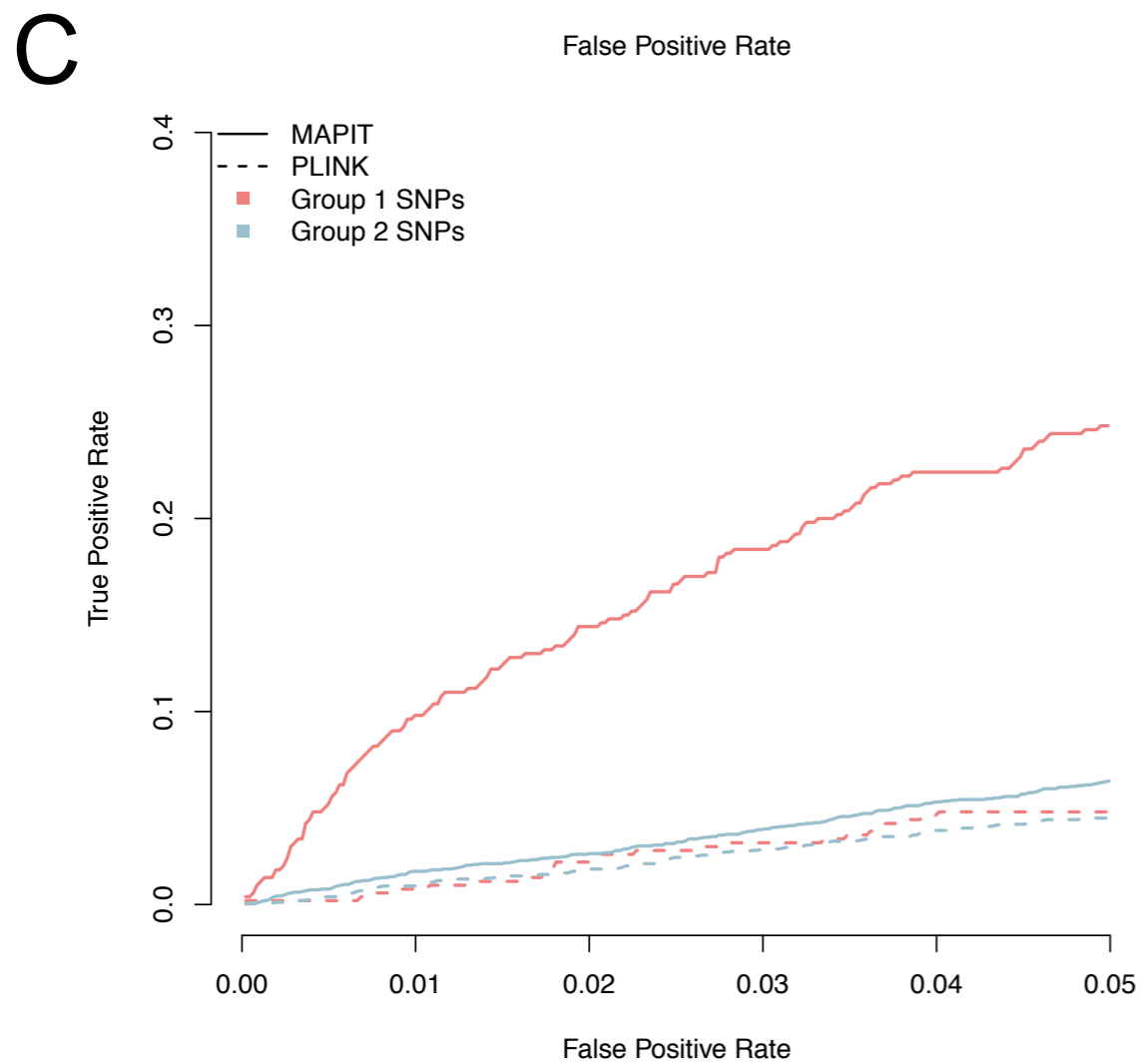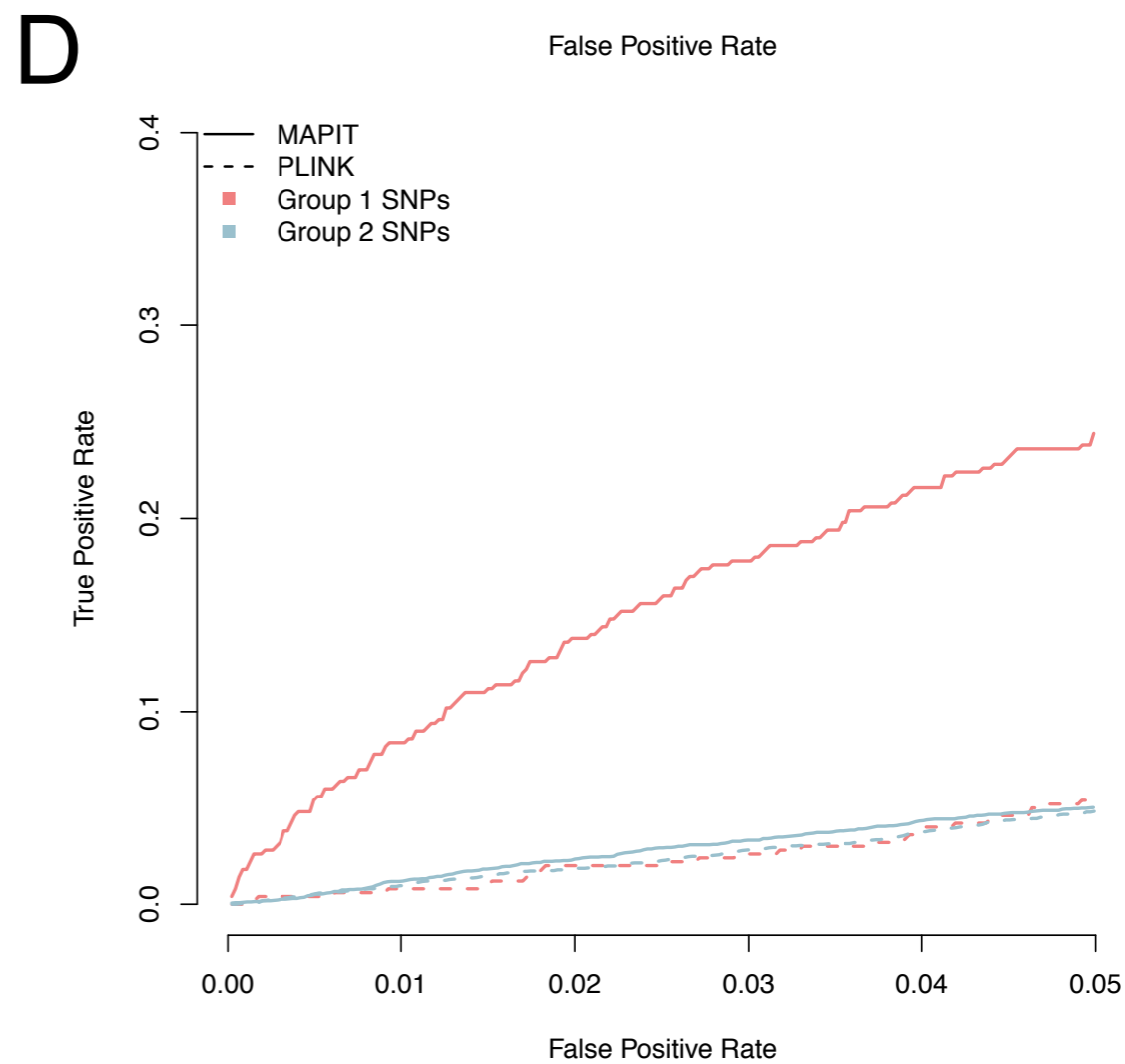

Supplement: S11 Fig — We compare the mapping abilities of MAPIT (solid line) to the exhaustive search procedure in PLINK (dotted line) in scenarios I (A), II (B), III (C), and IV (D), under broad-sense heritability level H2 = 0.6 and ρ = 0.8. Here, ρ = 0.8 was used to determine the portion of broad-sense heritability contributed by interaction effects. Group 1 (light red) and group 2 (light blue) causal SNPs. The x-axis shows the false positive rate, while the y-axis gives the rate at which true causal variants were identified. Results are based on 100 replicates in each case, where the data was created under simulation model (ii) with the top 5 genotype PCs. (PDF) [file pgen.1006869.s011.pdf]

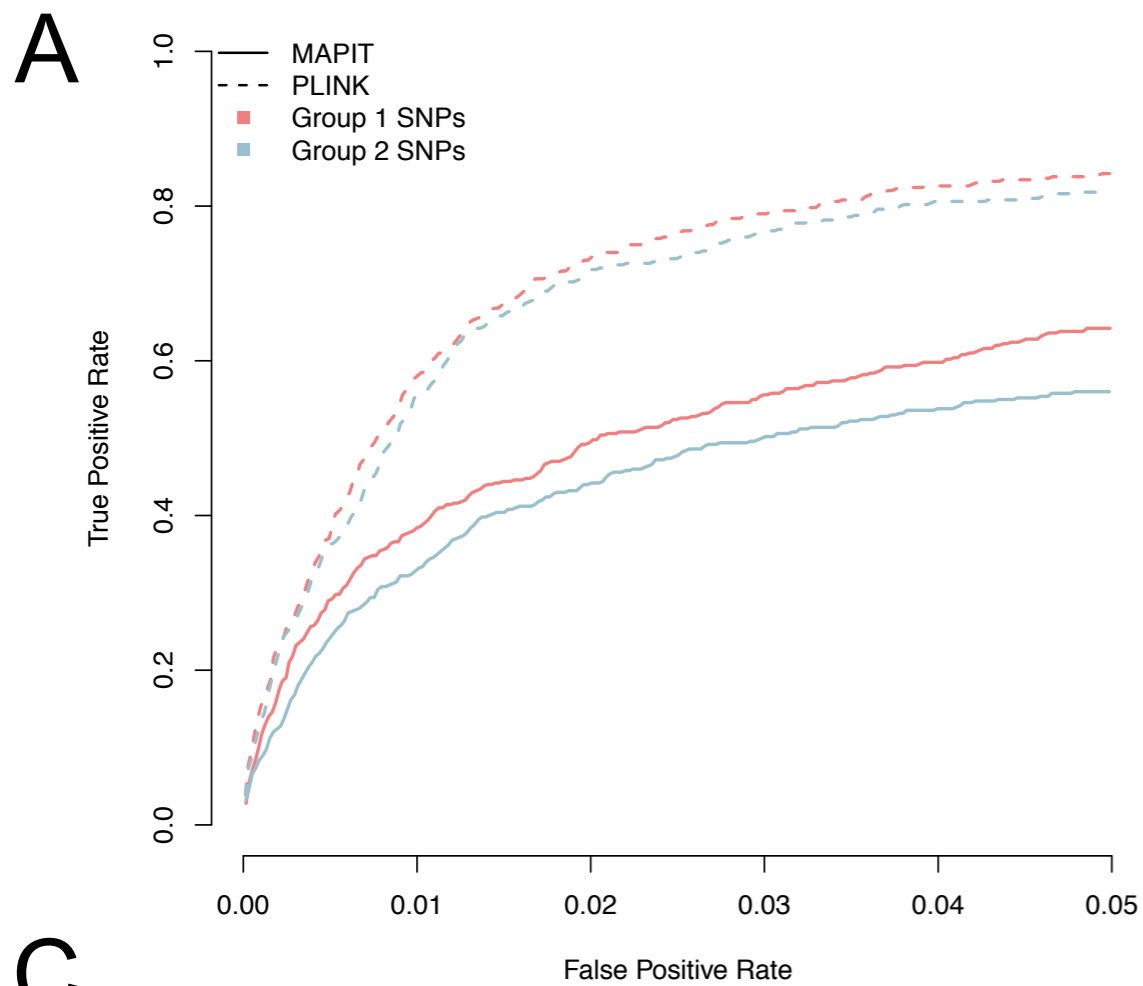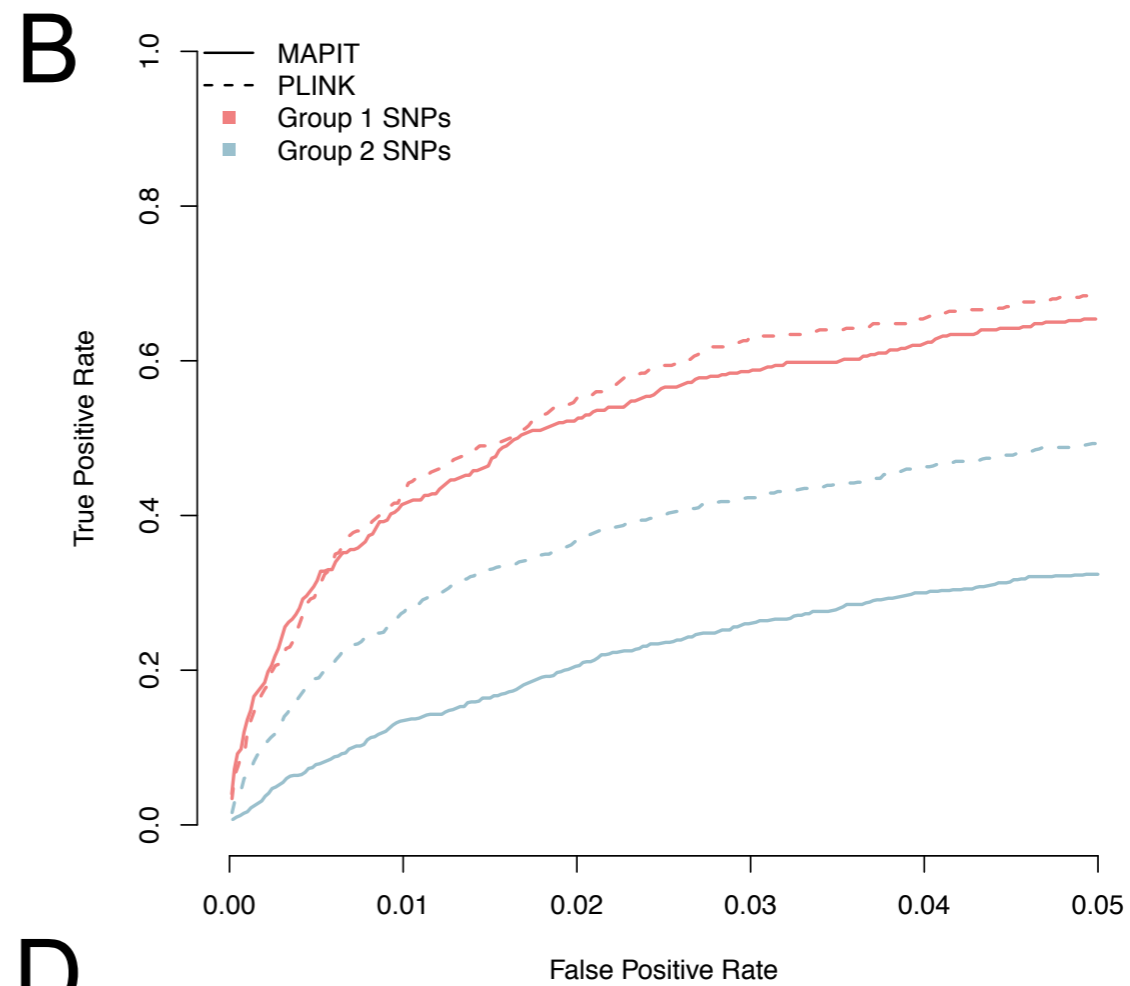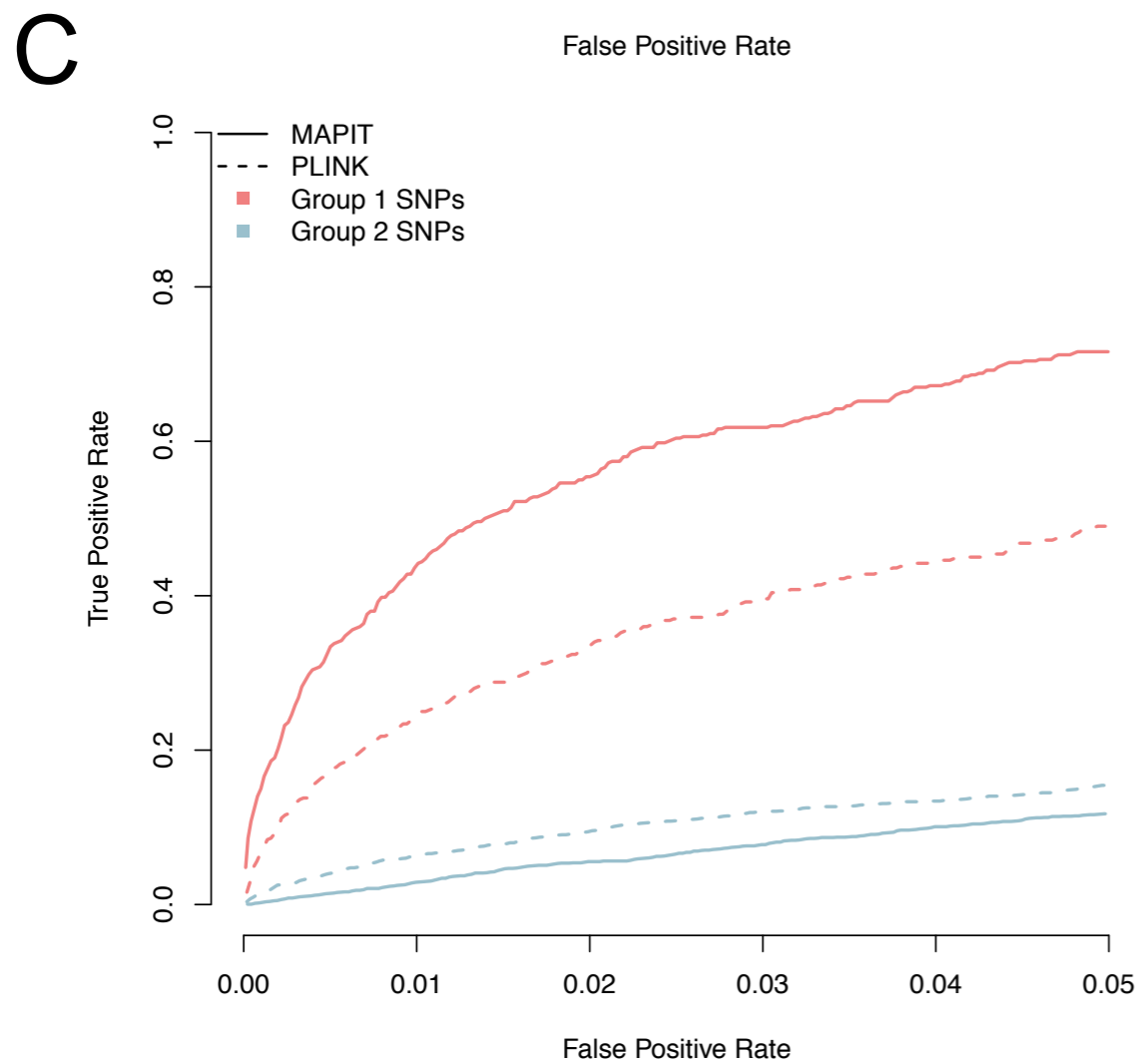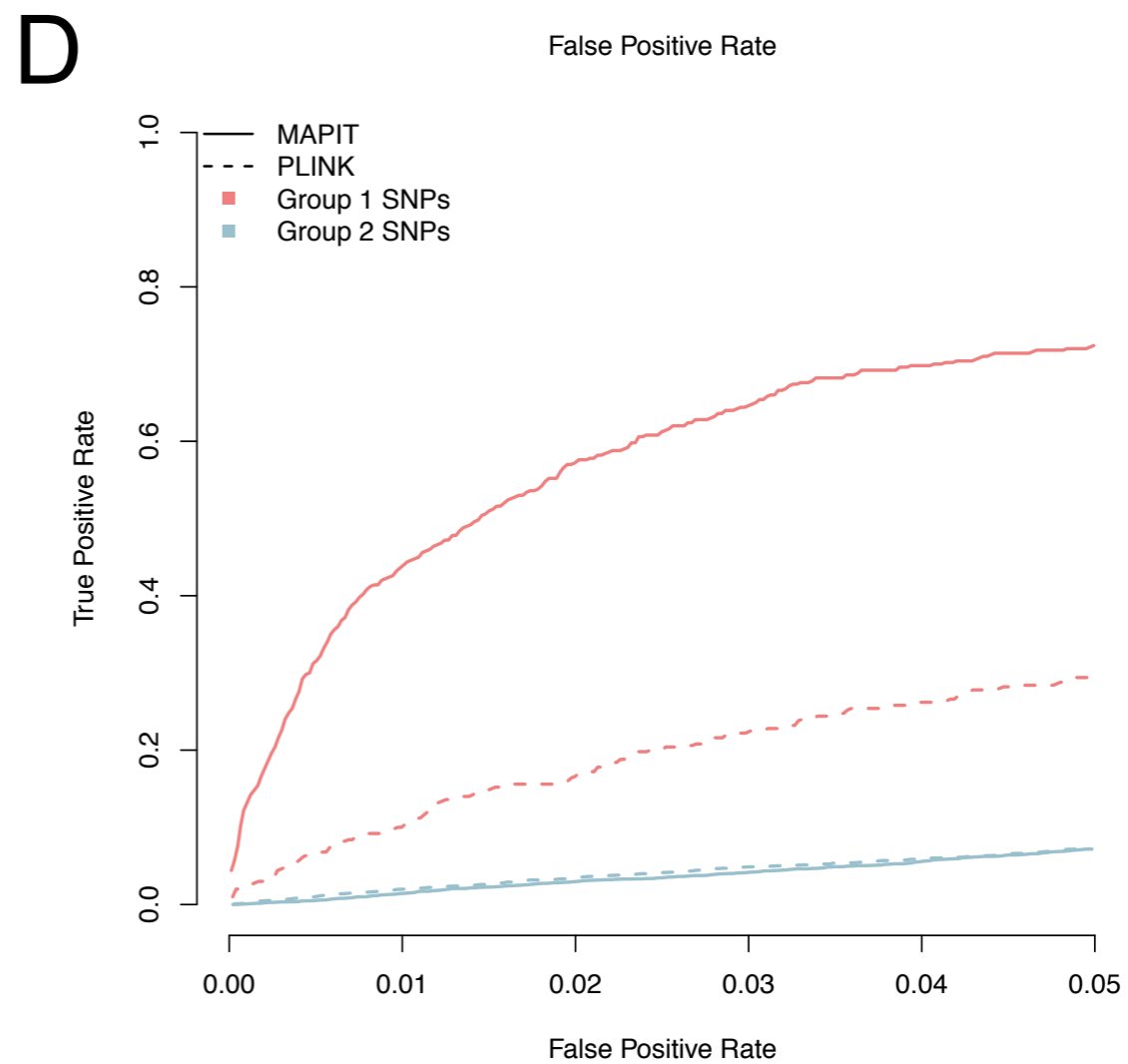

Supplement: S12 Fig — We compare the mapping abilities of MAPIT (solid line) to the exhaustive search procedure in PLINK (dotted line) in scenarios I (A), II (B), III (C), and IV (D), under broad-sense heritability level H2 = 0.6 and ρ = 0.5. Here, ρ = 0.5 was used to determine the portion of broad-sense heritability contributed by interaction effects. Group 1 (light red) and group 2 (light blue) causal SNPs. The x-axis shows the false positive rate, while the y-axis gives the rate at which true causal variants were identified. Results are based on 100 replicates in each case, where the data was created under simulation model (ii) with the top 10 genotype PCs. (PDF) [file pgen.1006869.s012.pdf]

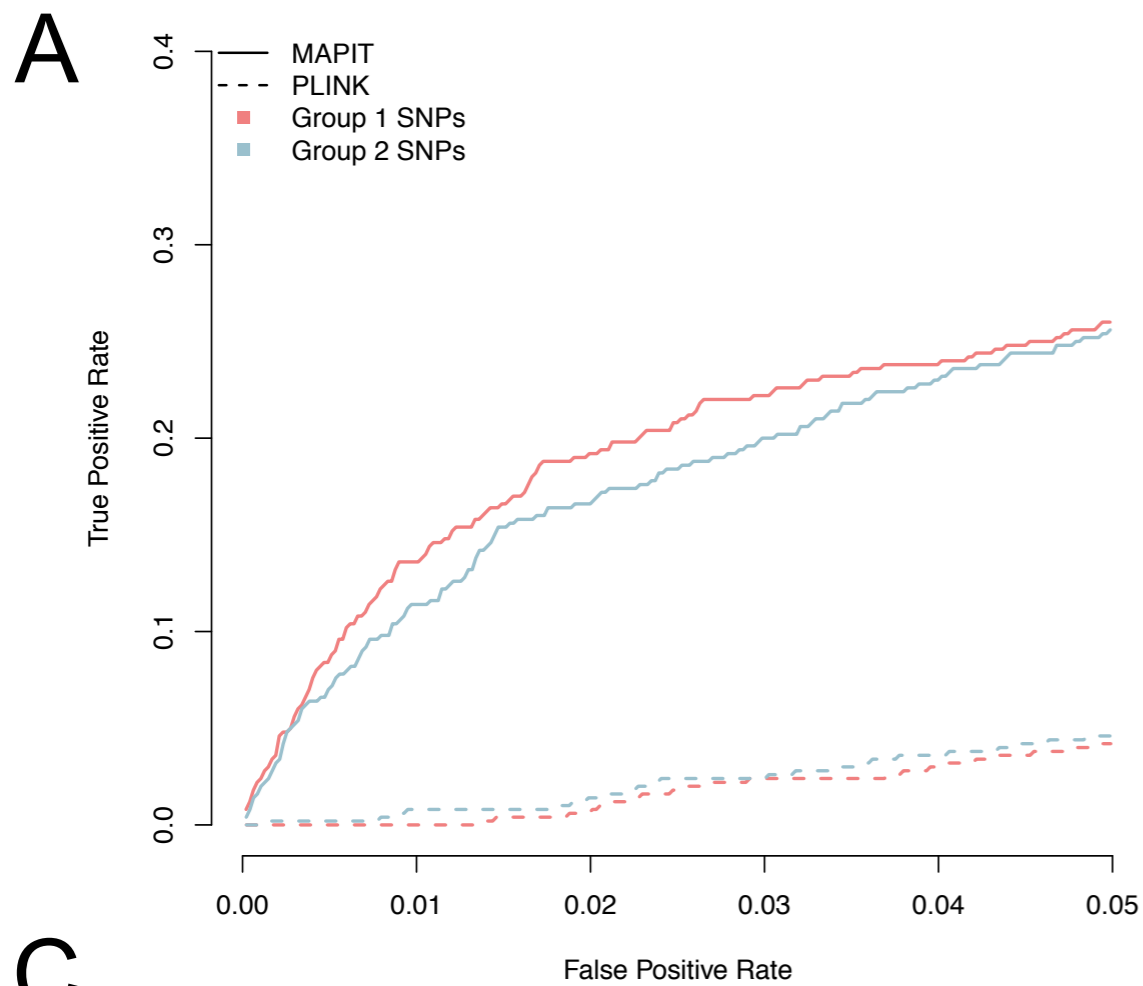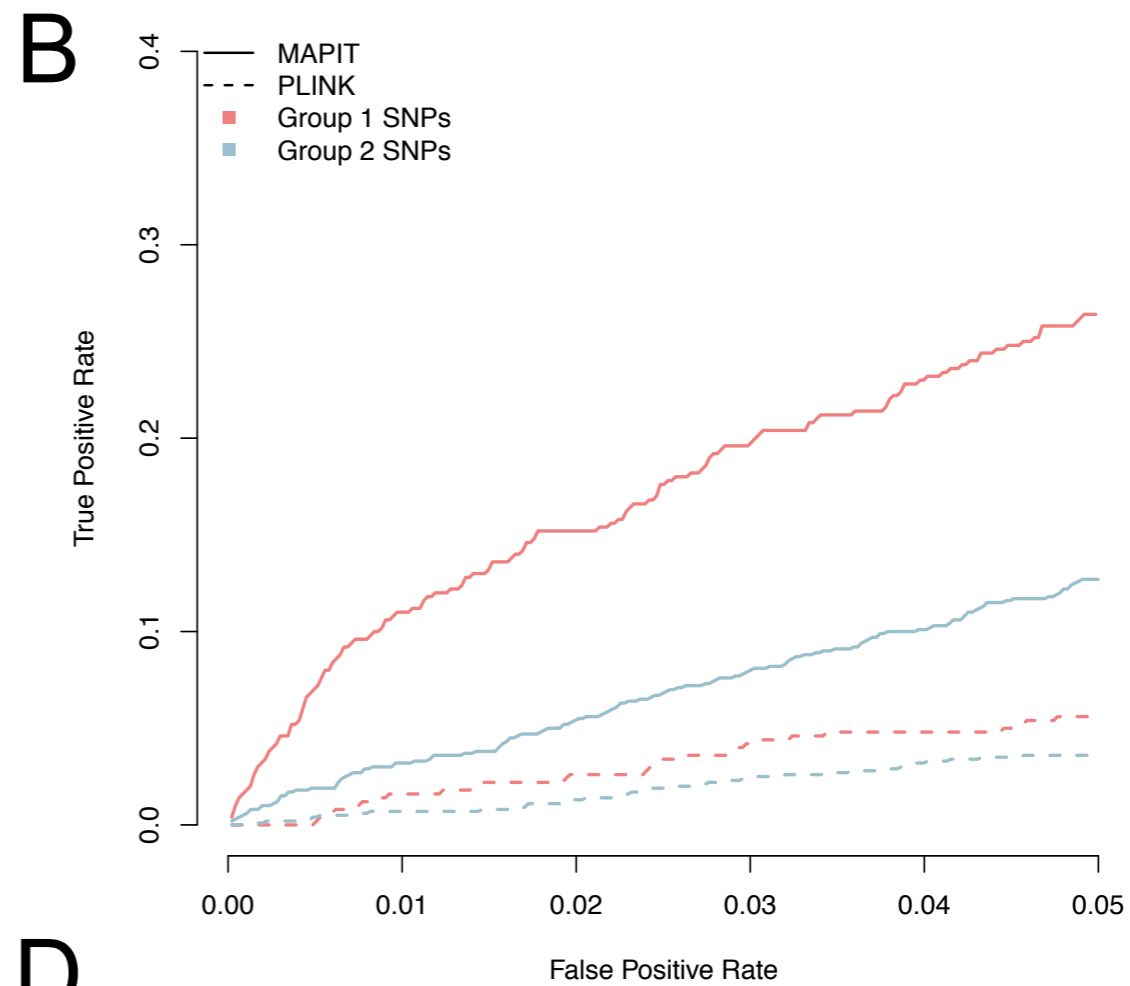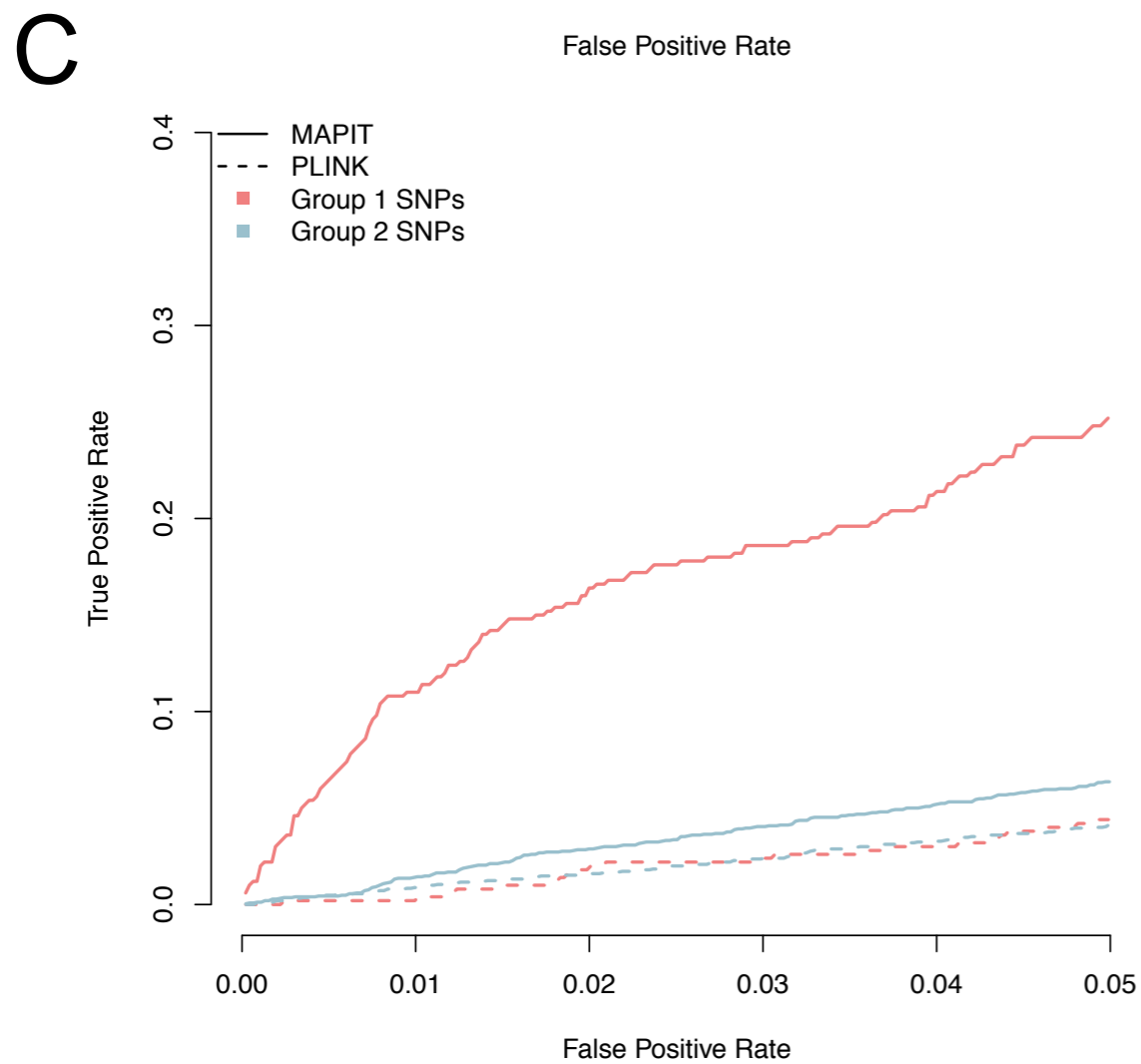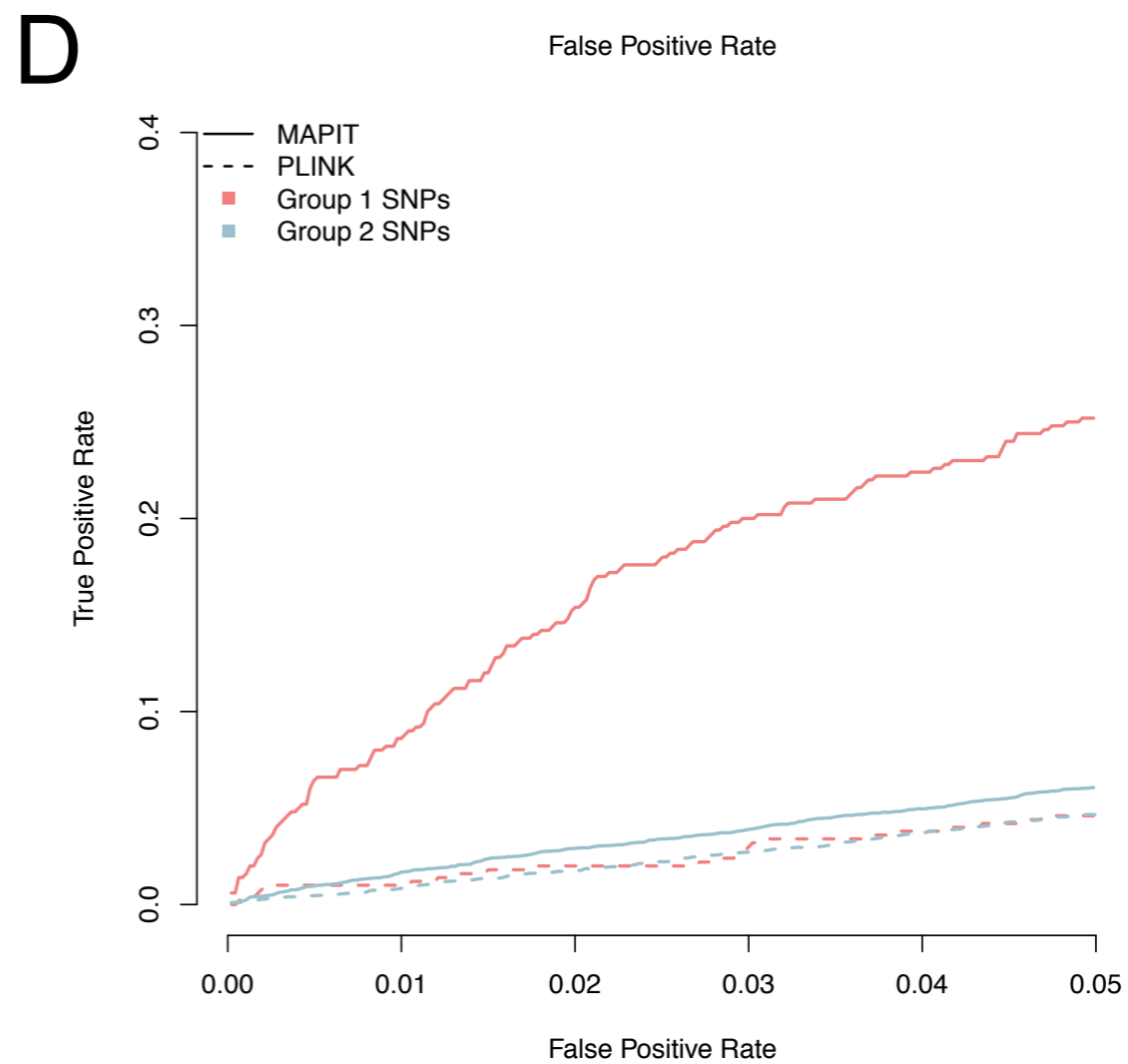

Supplement: S13 Fig — We compare the mapping abilities of MAPIT (solid line) to the exhaustive search procedure in PLINK (dotted line) in scenarios I (A), II (B), III (C), and IV (D), under broad-sense heritability level H2 = 0.6 and ρ = 0.8. Here, ρ = 0.8 was used to determine the portion of broad-sense heritability contributed by interaction effects. Group 1 (light red) and group 2 (light blue) causal SNPs. The x-axis shows the false positive rate, while the y-axis gives the rate at which true causal variants were identified. Results are based on 100 replicates in each case, where the data was created under simulation model (ii) with the top 10 genotype PCs. (PDF) [file pgen.1006869.s013.pdf]

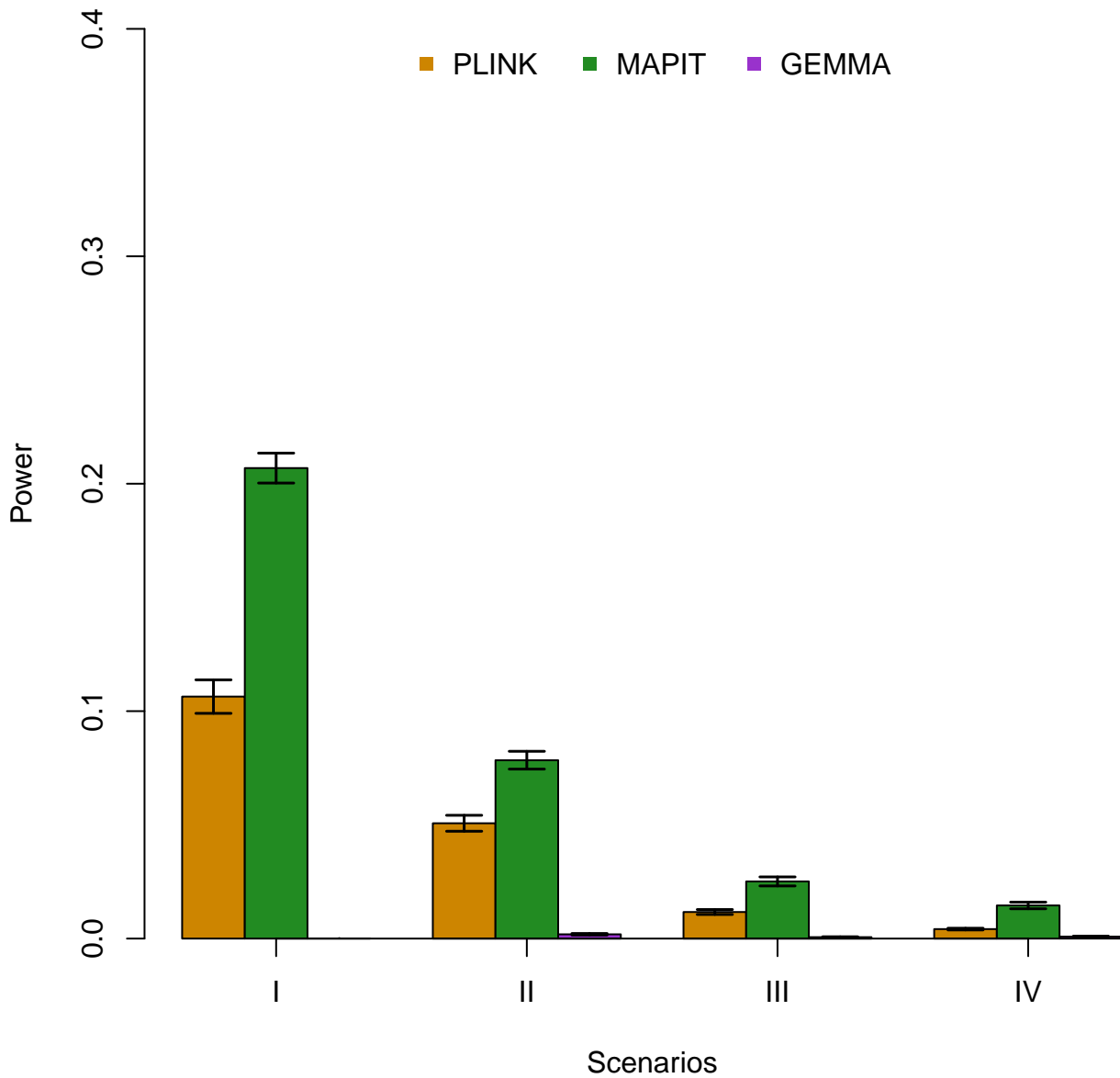

Supplement: S14 Fig — Here, the effectiveness of MAPIT (green) as an initial step in a pairwise detection filtration process is compared against the more conventional single-SNP testing procedure, which is carried out via GEMMA (purple). In both cases, the search for epistatic pairs occurs between the top 100 significant marginally associated SNPs are considered. We use the fully exhaustive search model in PLINK (orange) as a baseline comparison. We compare the three methods in all scenarios (x-axis), under broad-sense heritability level H2 = 0.6. Here, ρ = 0.5 was used to determine the portion of broad-sense heritability contributed by interaction effects. The y-axis gives the rate at which true causal epistatic pairs were identified. Results are based on 100 replicates in each case. The lines represent 95% variability due to resampling error. (PDF) [file pgen.1006869.s014.pdf]

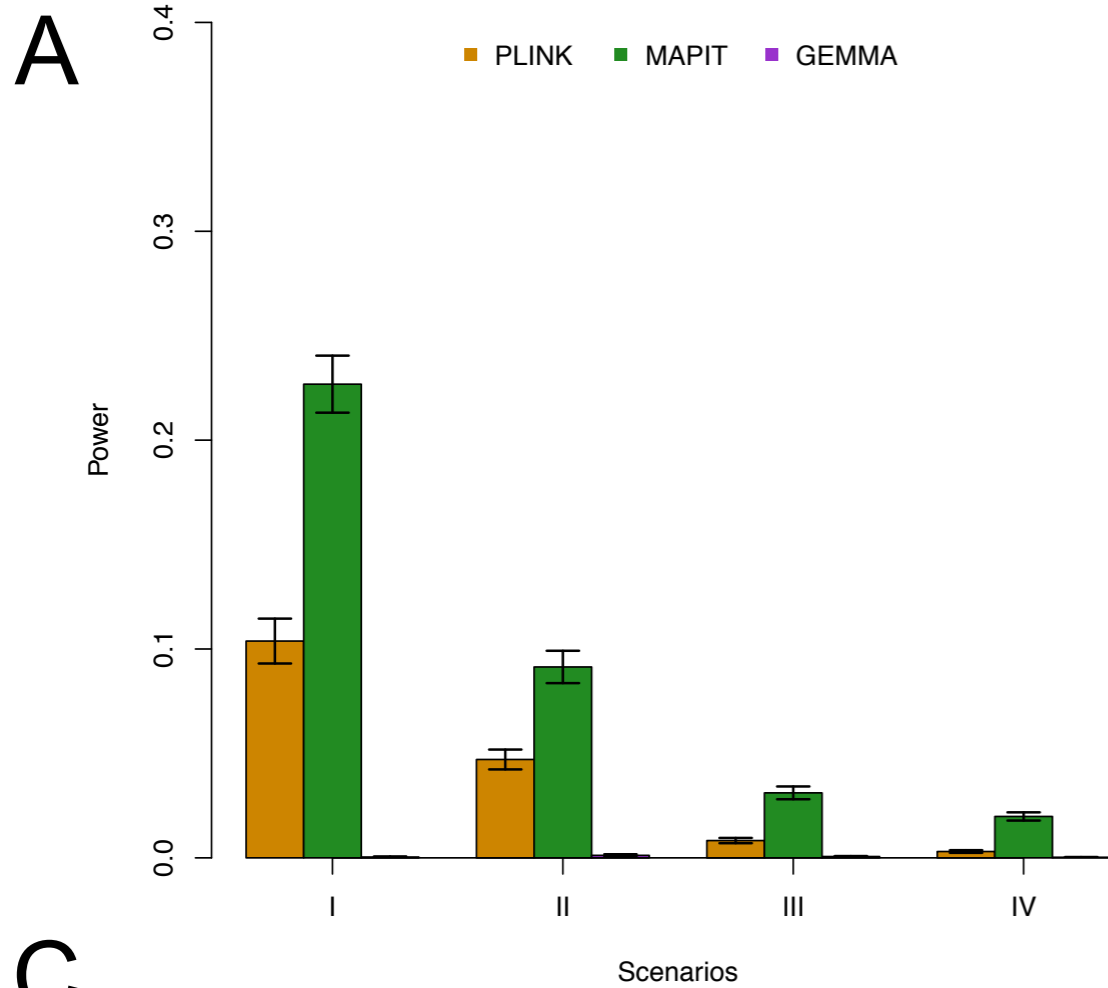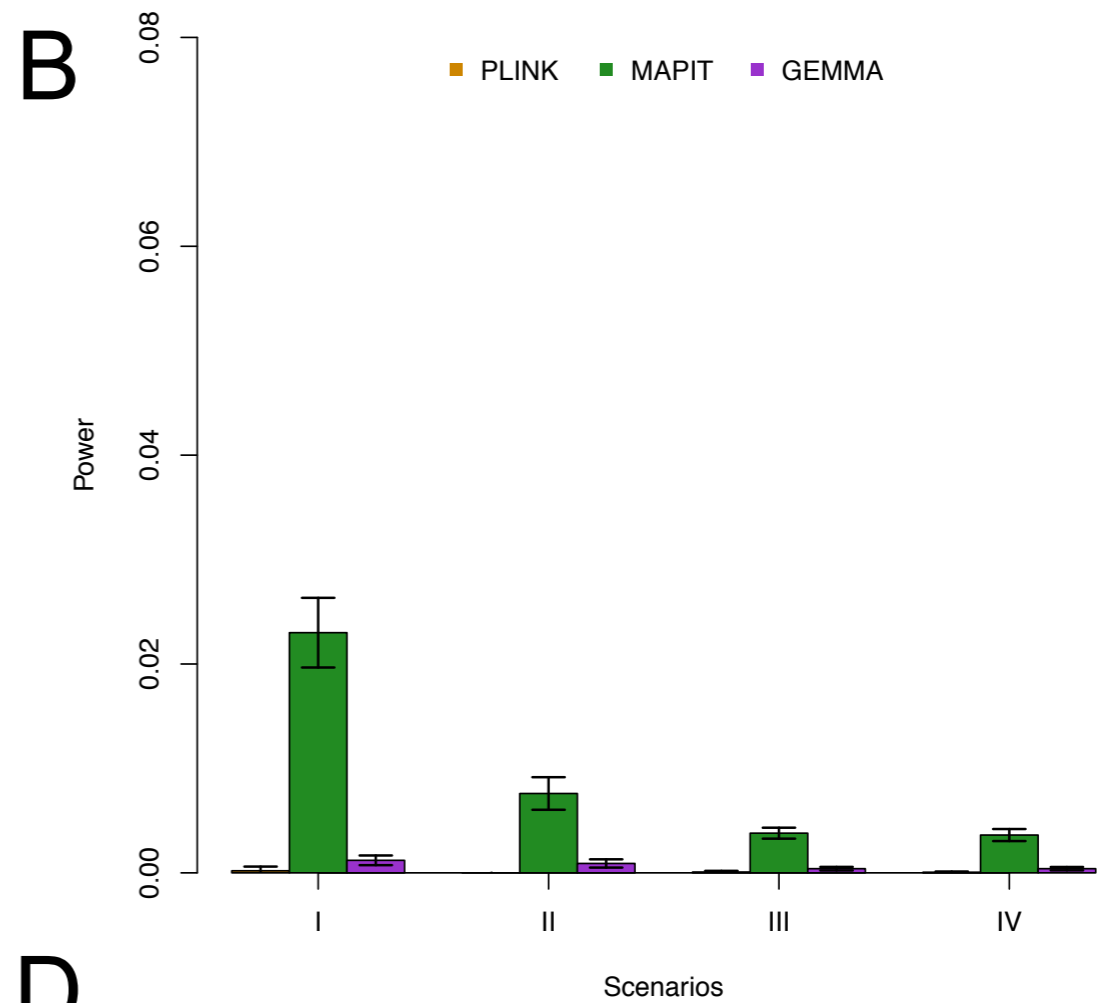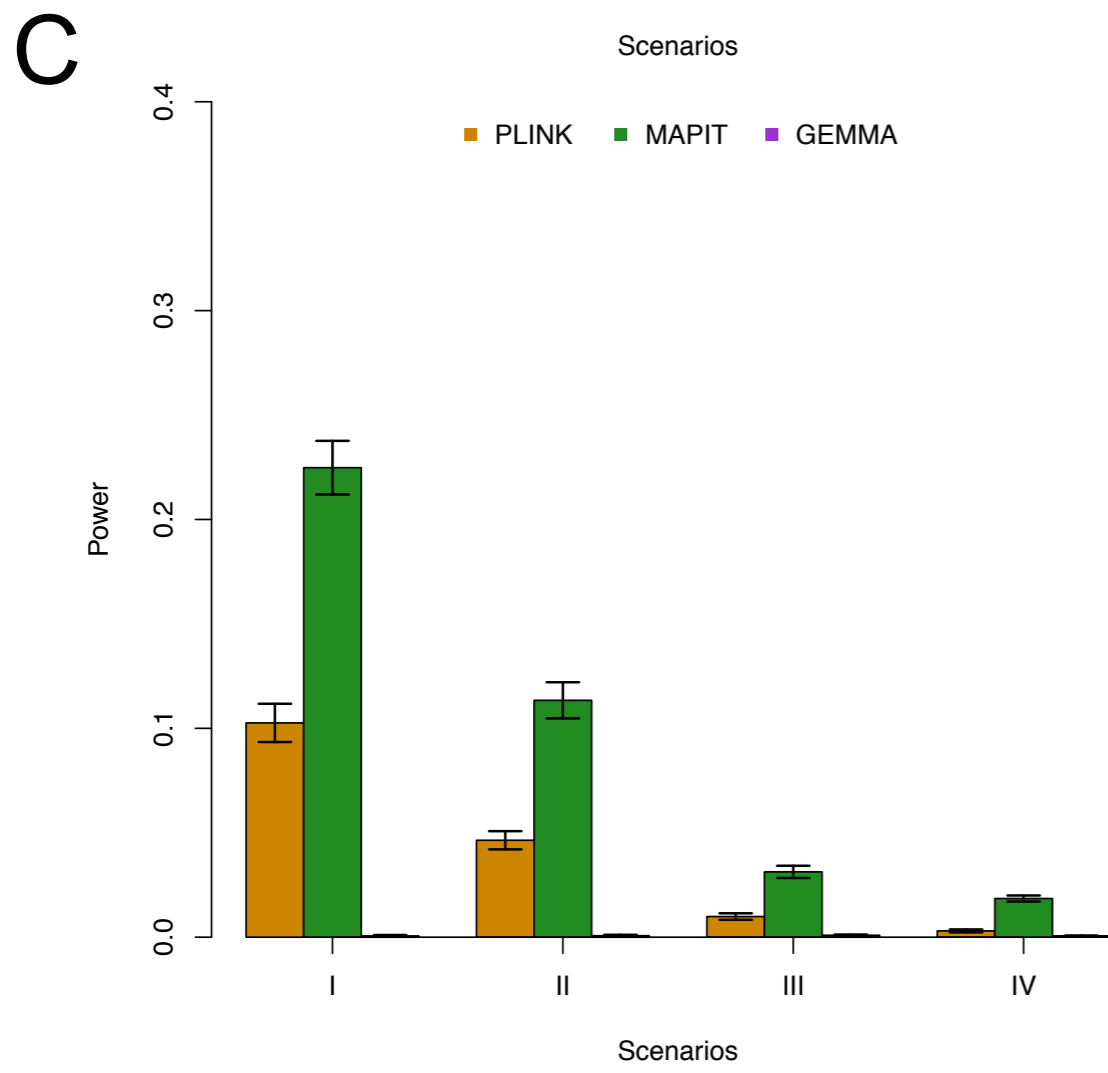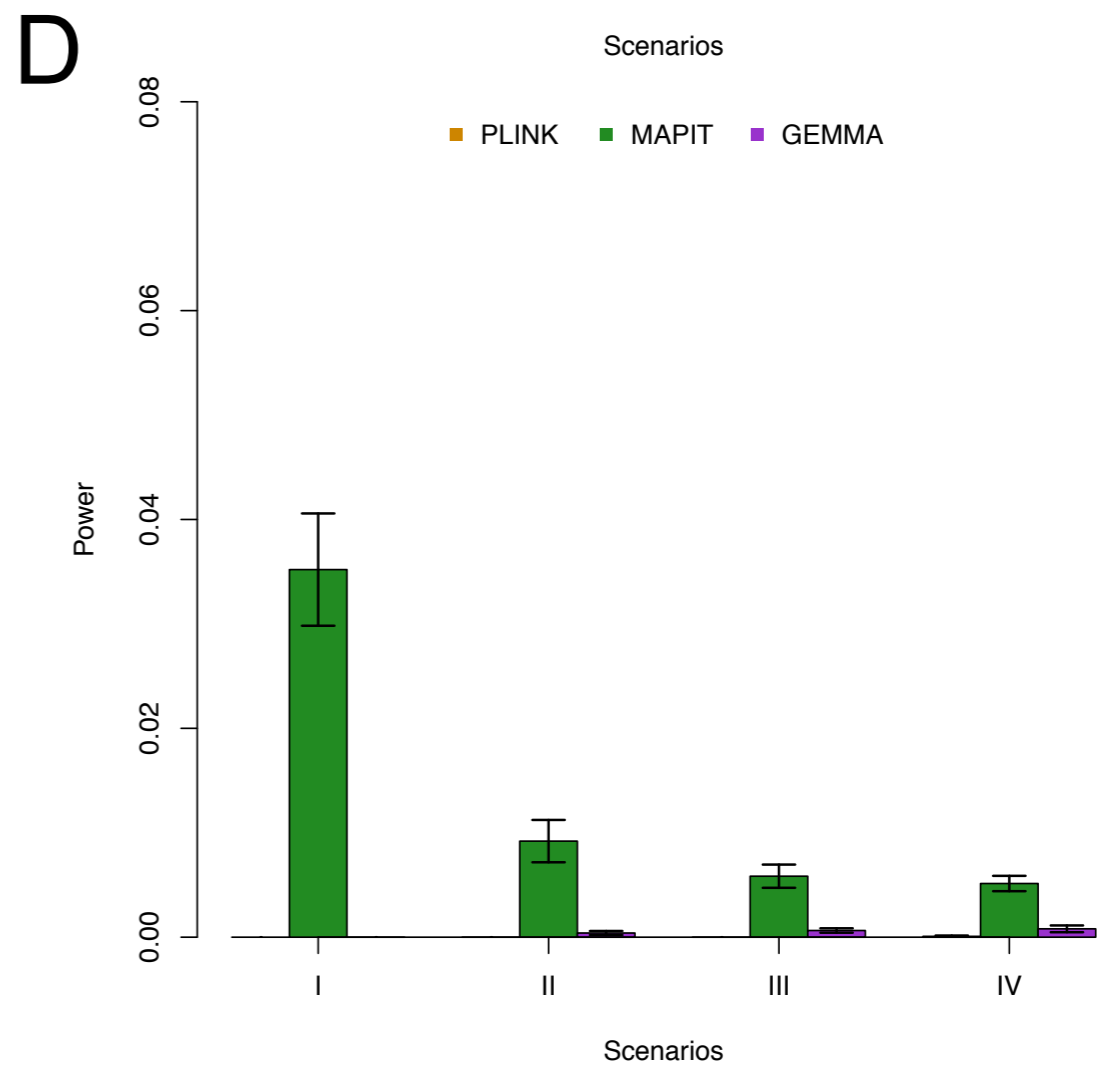

Supplement: S15 Fig — Here, the effectiveness of MAPIT (green) as an initial step in a pairwise detection filtration process is compared against the more conventional single-SNP testing procedure, which is carried out via GEMMA (purple). In both cases, the search for epistatic pairs occurs between the top 100 significant marginally associated SNPs are considered. We use the fully exhaustive search model in PLINK (orange) as a baseline comparison. We compare the three methods in all scenarios (x-axis), under broad-sense heritability level H2 = 0.6. Here, ρ = 0.5 (A, C) and ρ = 0.8 (B, D) were used to determine the portion of broad-sense heritability contributed by interaction effects. The y-axis gives the rate at which true causal epistatic pairs were identified. Results are based on 100 replicates in each case, where the data was created under simulation model (ii). (A) and (B) use the top 5 genotype PCs, while (C) and (D) use the top 10 genotypes PCs. The lines represent 95% variability due to resampling error. (PDF) [file pgen.1006869.s015.pdf]

**A**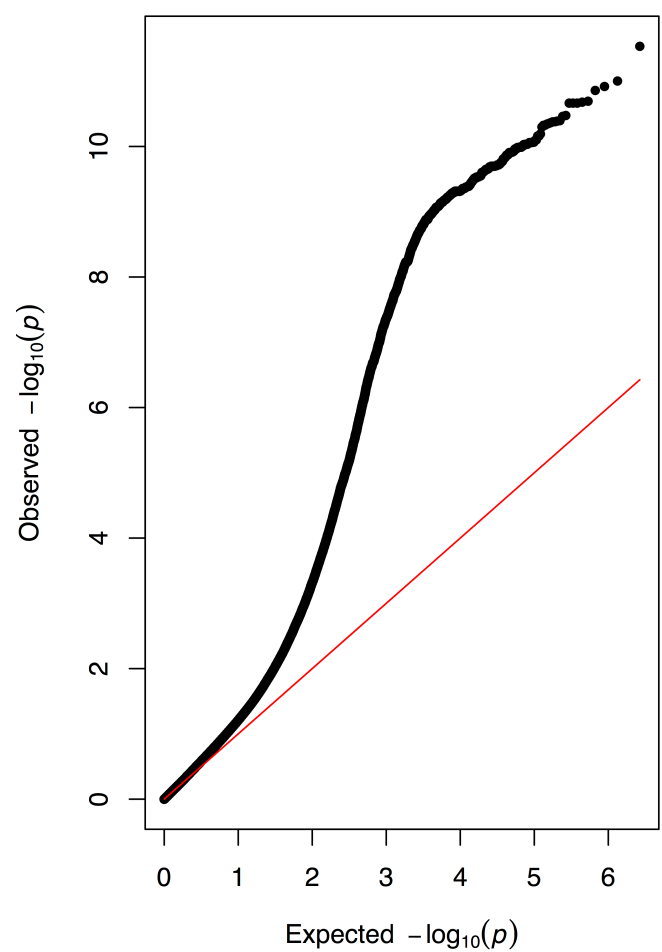**B**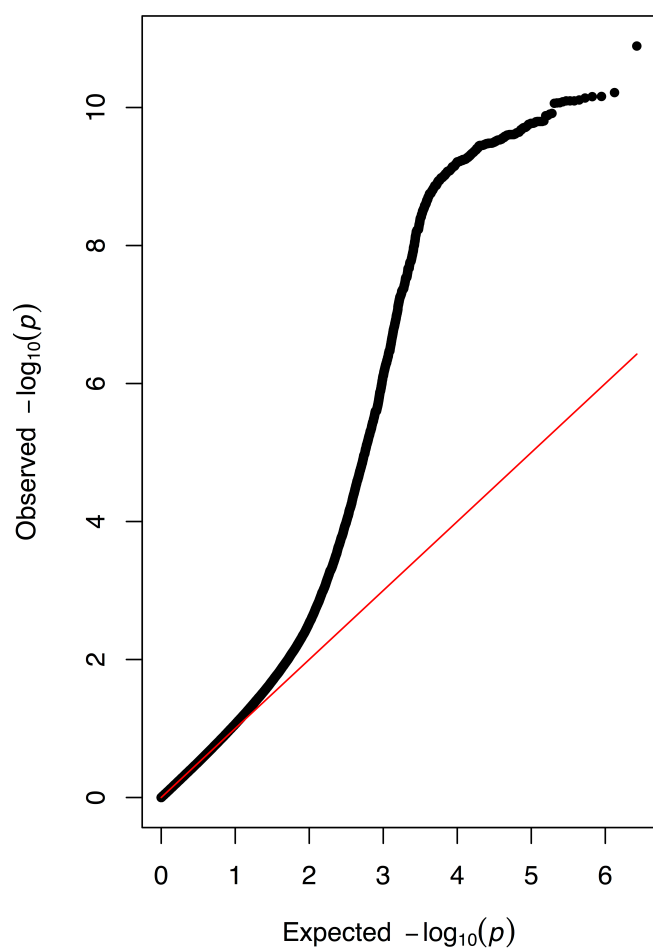**C**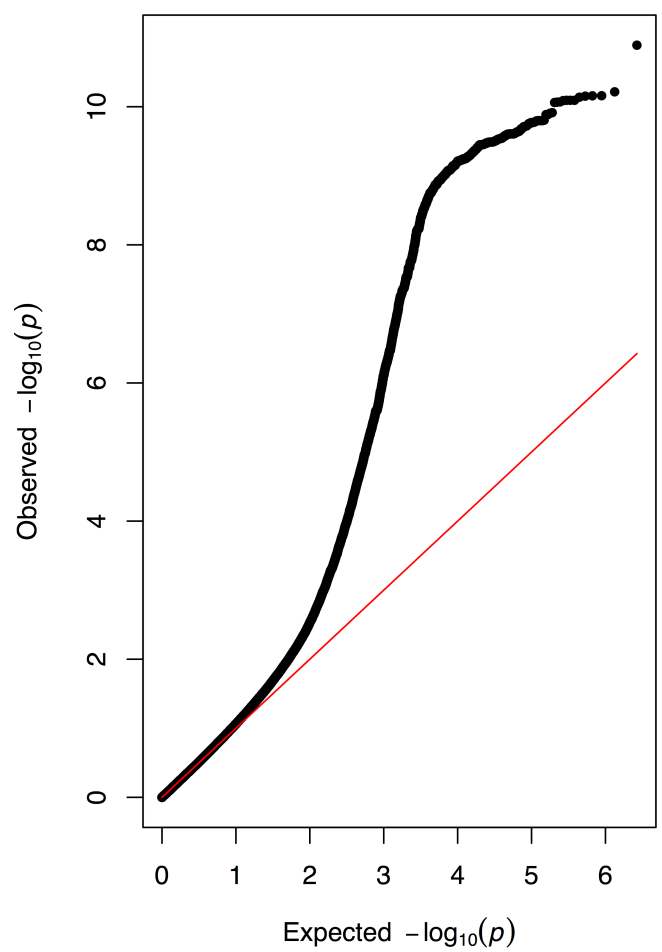**D**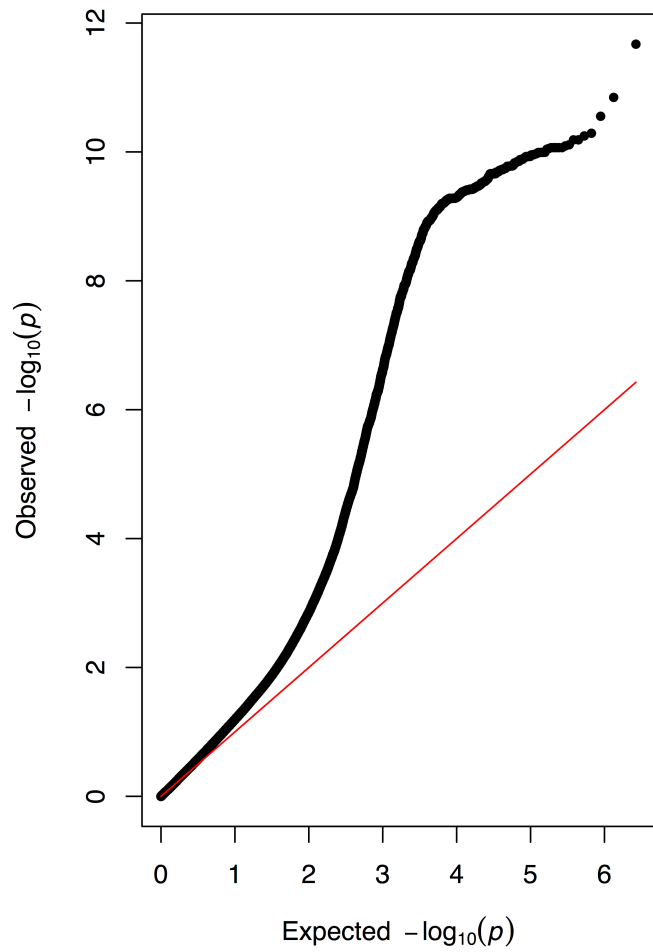

Supplement: S16 Fig — (A) corresponds to using MAPIT with a genetic relatedness matrix Kcis, where for the expression of each gene Kcis was computed using only the corresponding cis-SNPs. (B) corresponds to using MAPIT with a genetic relatedness matrix Ktrans, where for the expression of each gene Ktrans was computed using SNPs outside of the corresponding cis-window. (C) corresponds to using MAPIT with a genome-wide genetic relatedness matrix KGW, where KGW was computed using all SNPs in the study. (D) illustrates results from using MAPIT with KPop, which was computed after we first controlled for residual population stratification effects. (PDF) [file pgen.1006869.s016.pdf]

A

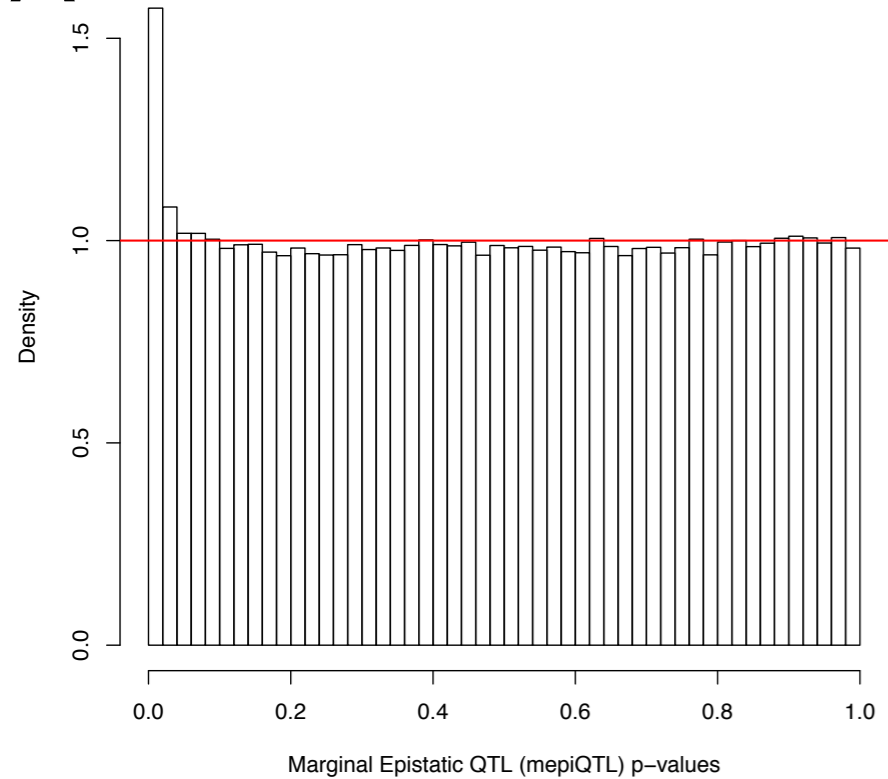

B

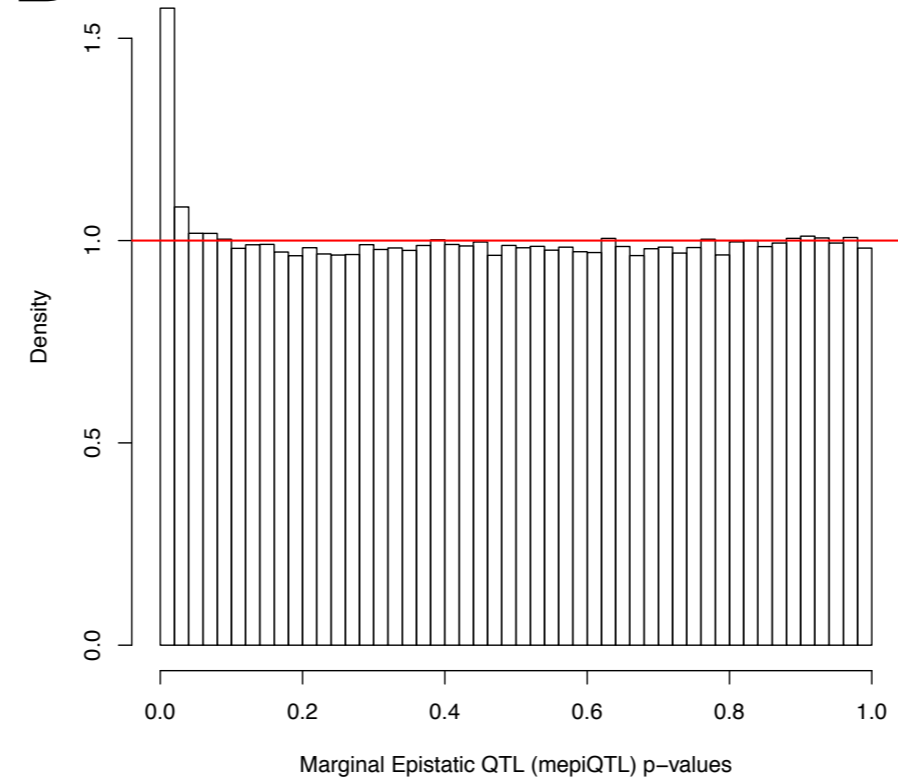

C

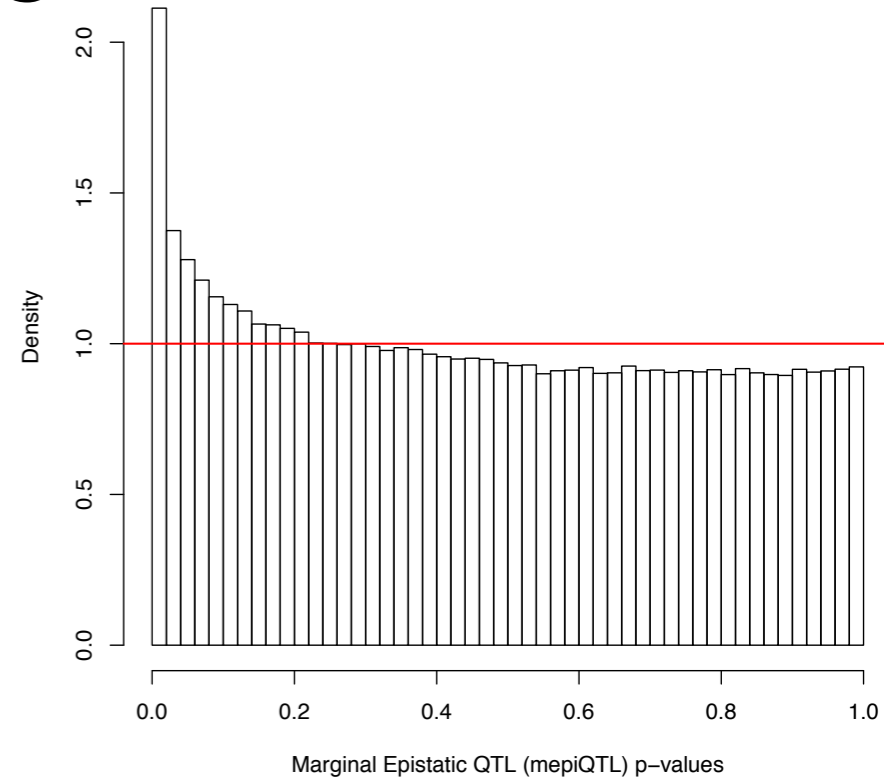

D

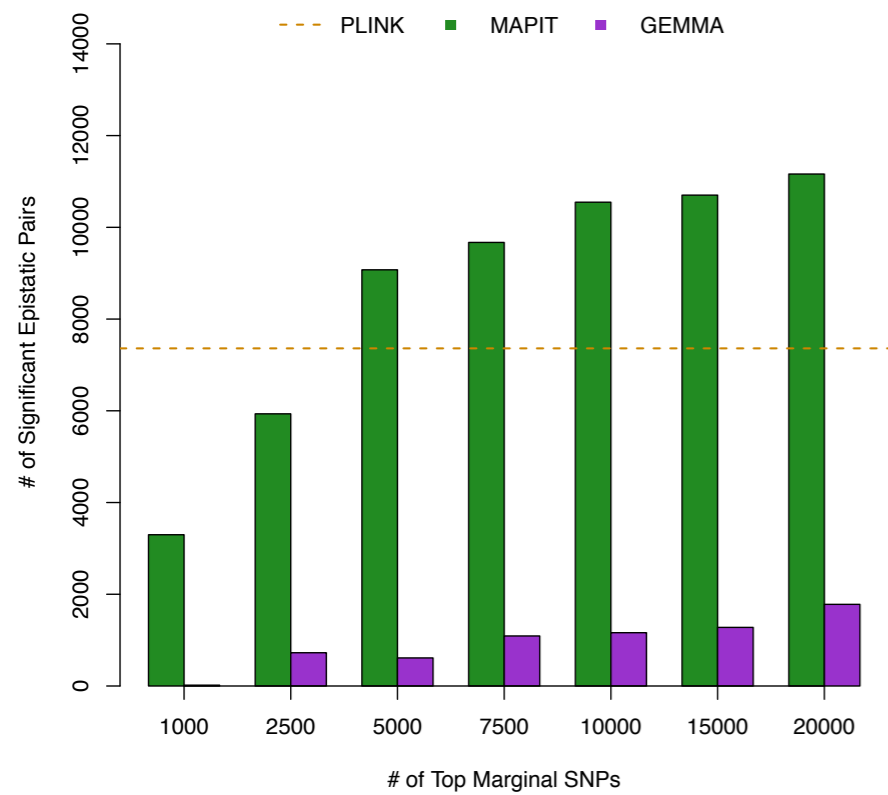

E

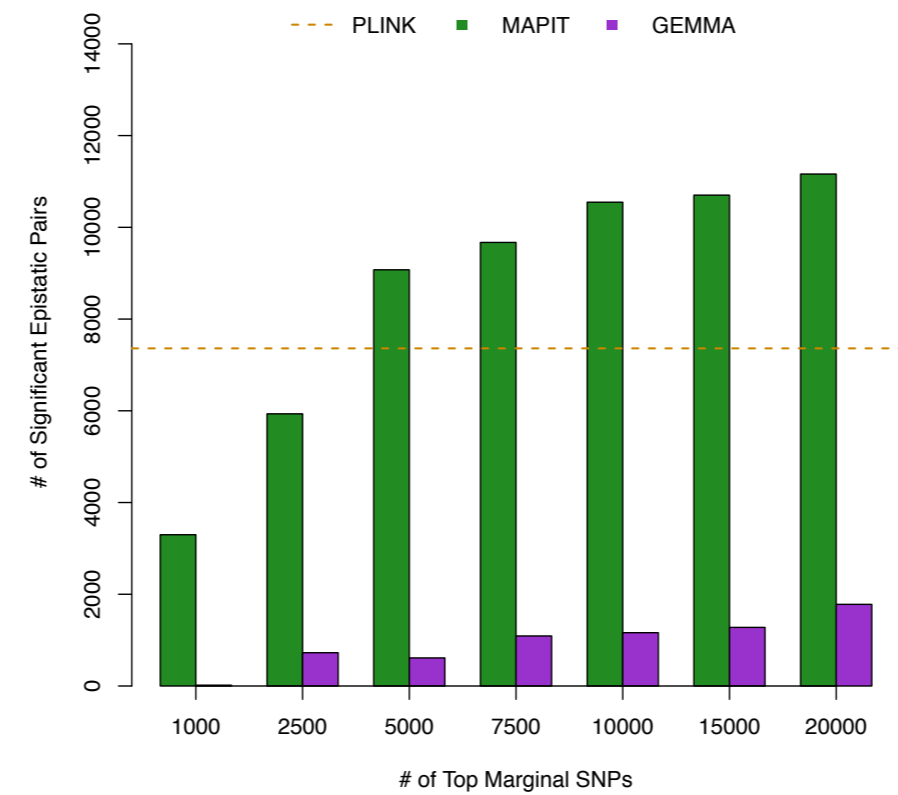

F

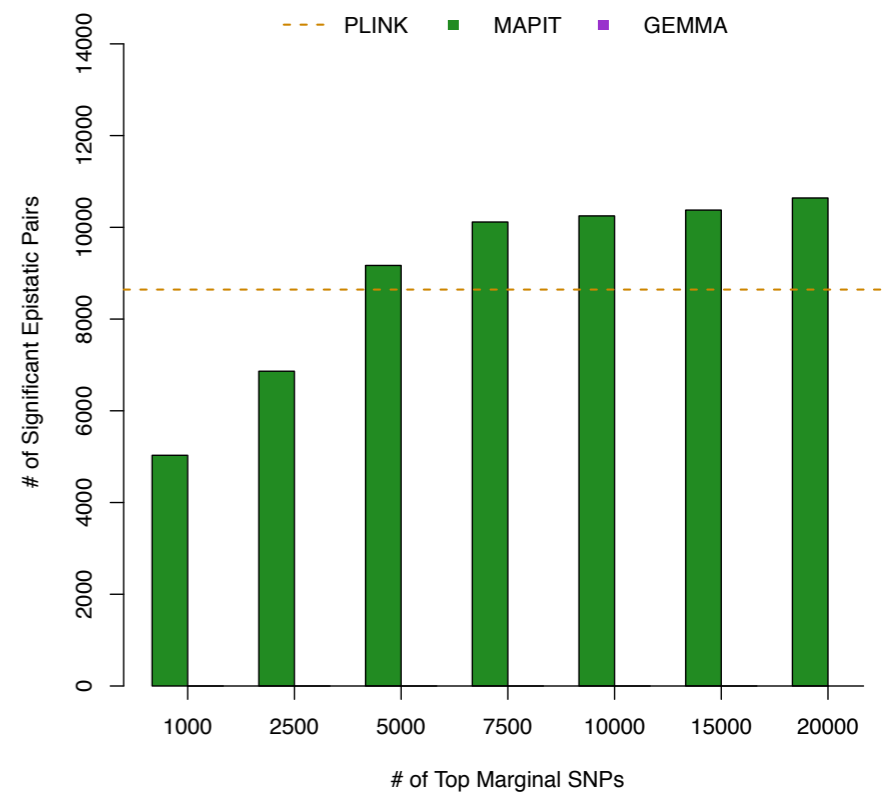

Supplement: S17 Fig — (A)-(C) show a histograms of the MAPIT p-values for all variants in the GEUVADIS data set using the genome-wide genetic relatedness matrix Ktrans (A), KGW (B), and KPop (C), respectively. The horizontal red line corresponds to a uniform distribution of p-values. (D)-(F) show the number of significant pairwise interactions (y-axis) identified by MAPIT (green) and GEMMA (purple) when searching between the top v = {1000, 2500, 5000, 7500, 10000, 15000, 20000} marginally associated variants (x-axis). We use the number of significant pairs identified by fully exhaustive search model in PLINK as a baseline comparison (orange dotted line). Note that PLINK only analyzes cis-SNPs pairs, while MAPIT with Ktrans (D), KGW (E), and KPop (F) effectively analyzes the marginal epistatic effects of cis-SNPs with all genome-wide SNPs. This image shows the distributions of genome-wide significant epistatic pairs as found by each method. An interaction for MAPIT and GEMMA was deemed signifiant if it had a joint p-value below the threshold P = 0.05/(∑i qi(qi − 1)/2), where qi is the number of top variants located in the cis-window of gene i. In the case of PLINK, we consider two variants to be a significantly associated epistatic pair if they have a joint p-value below the threshold P = 1.09 × 10^−10^, which corresponds to the Bonferroni-correction that would be used if we examined all possible genome-wide SNP pairs across all genes in the final data set. Overall, PLINK detected 7,361 (D, E), and 8,643 (F) significant pairwise interactions. (PDF) [file pgen.1006869.s017.pdf]

**A**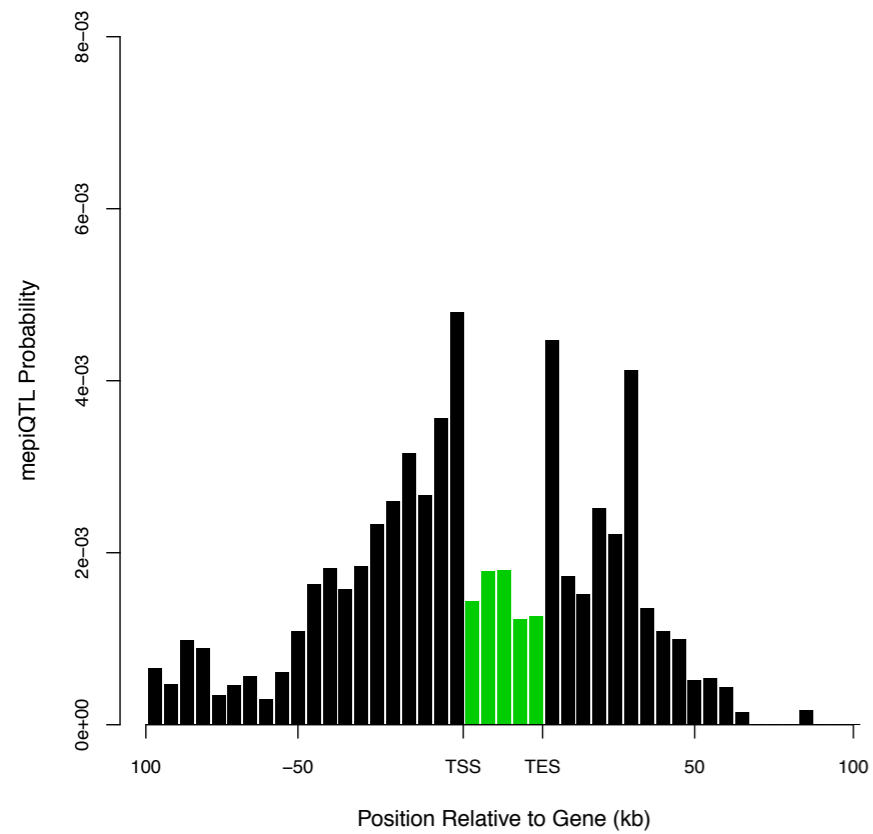**B**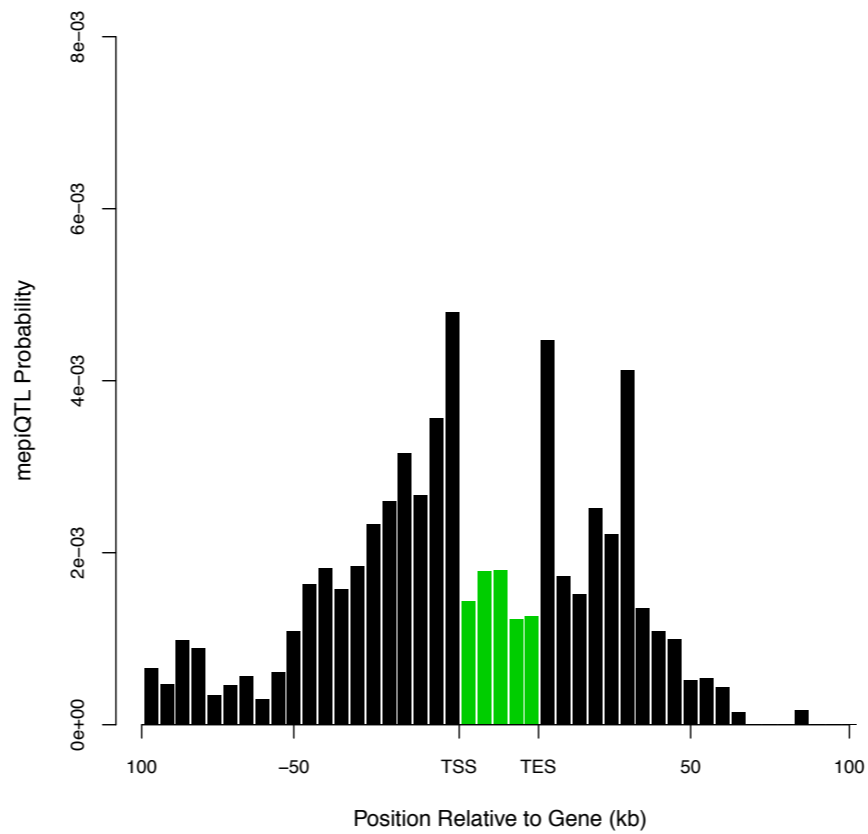**C**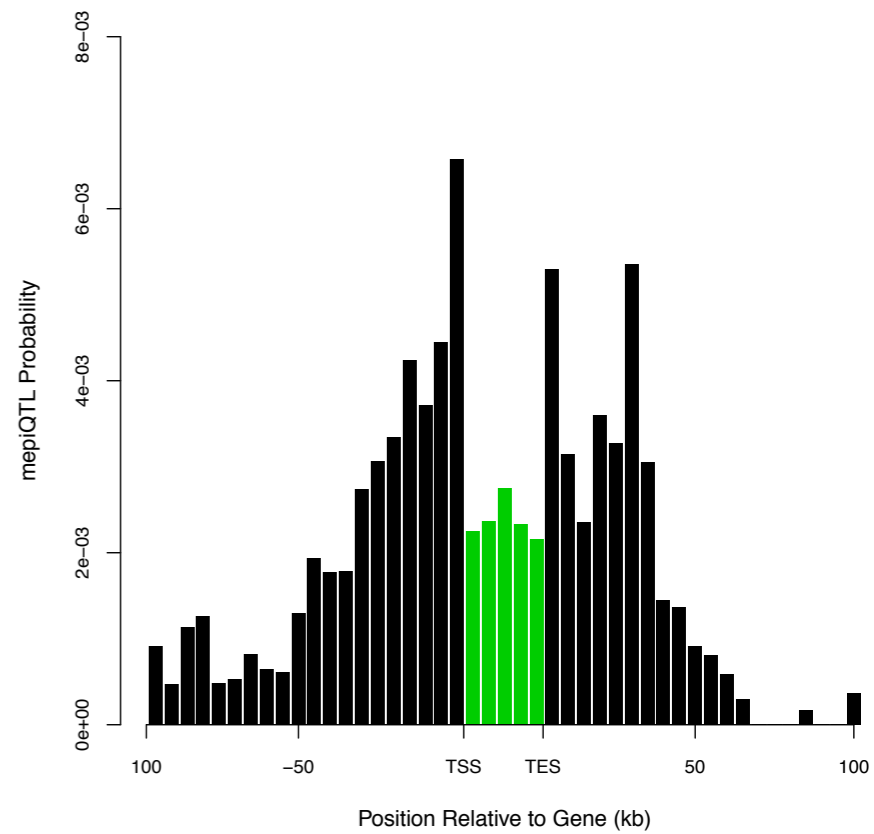

Supplement: S18 Fig — Shown here are the distribution of locations for significant SNPs, relative to the 5′ most gene transcription start site (TSS) and the 3′ most gene transcription end site (TES). (A) displays the marginally epistatic QTL (mepiQTL) detected by MAPIT using the genetic relatedness matrix Ktrans. (B) displays the marginally epistatic QTL (mepiQTL) detected by MAPIT using the genetic relatedness matrix KGW. (C) corresponds to the mepiQTL identified by MAPIT using KPop. The x-axis of each plot divides a typical cis-candidate region into a series of bins. The y-axis plots the number of SNPs in each bin that have a p-value less than a gene specific Bonferroni-corrected significance p-value threshold P = 0.05/∑i si, where si is the number of cis-SNPs for gene i, divided by the total number of SNPs in that bin. Bars in green denote the region bounded by the TSS and TES, with gene lengths divided into 20 bins for visibility—because the gene body is thus artificially enlarged, SNP density within genes cannot be directly compared with SNP density outside of genes. (PDF) [file pgen.1006869.s018.pdf]

A

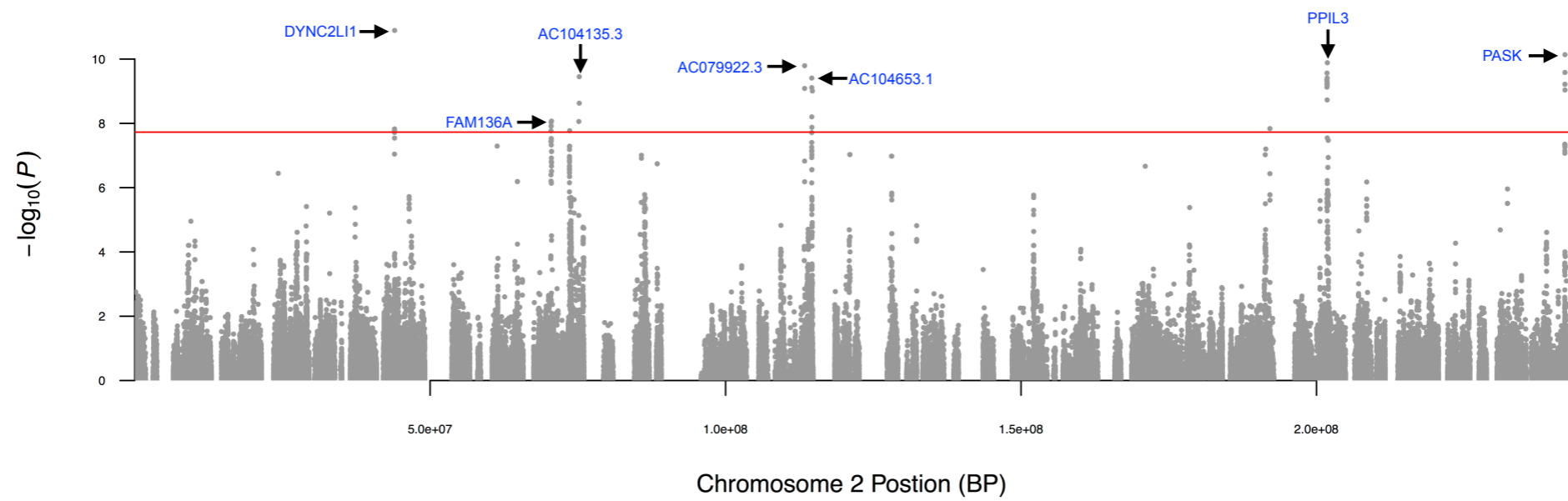

B

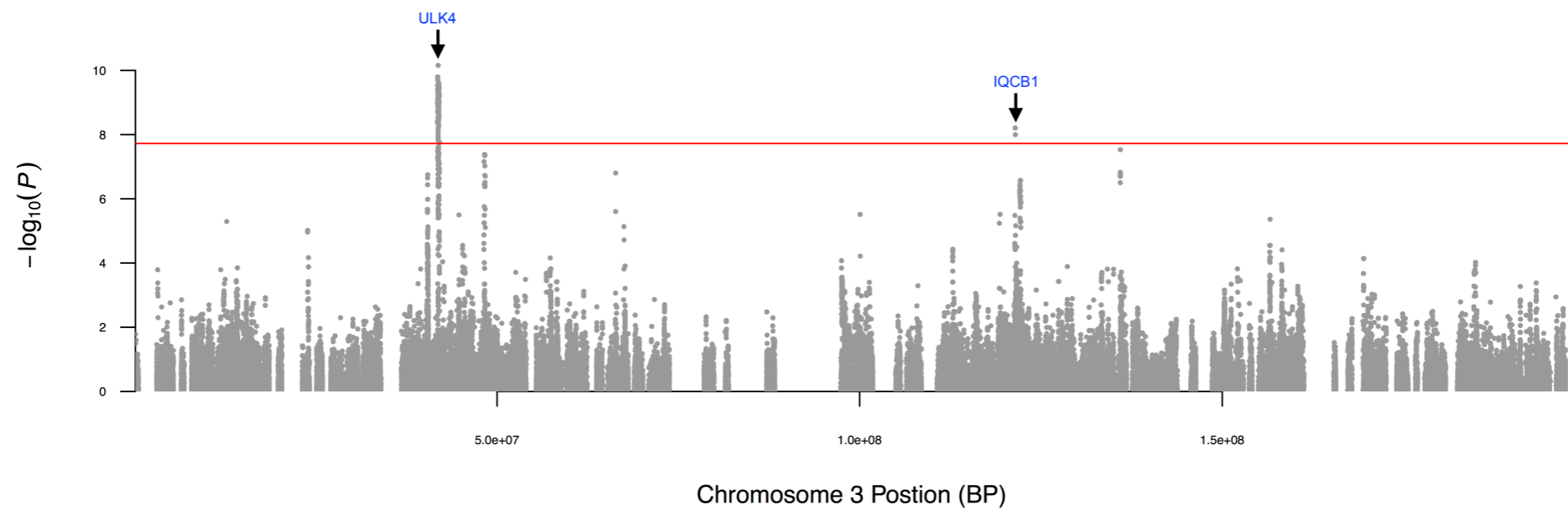

C

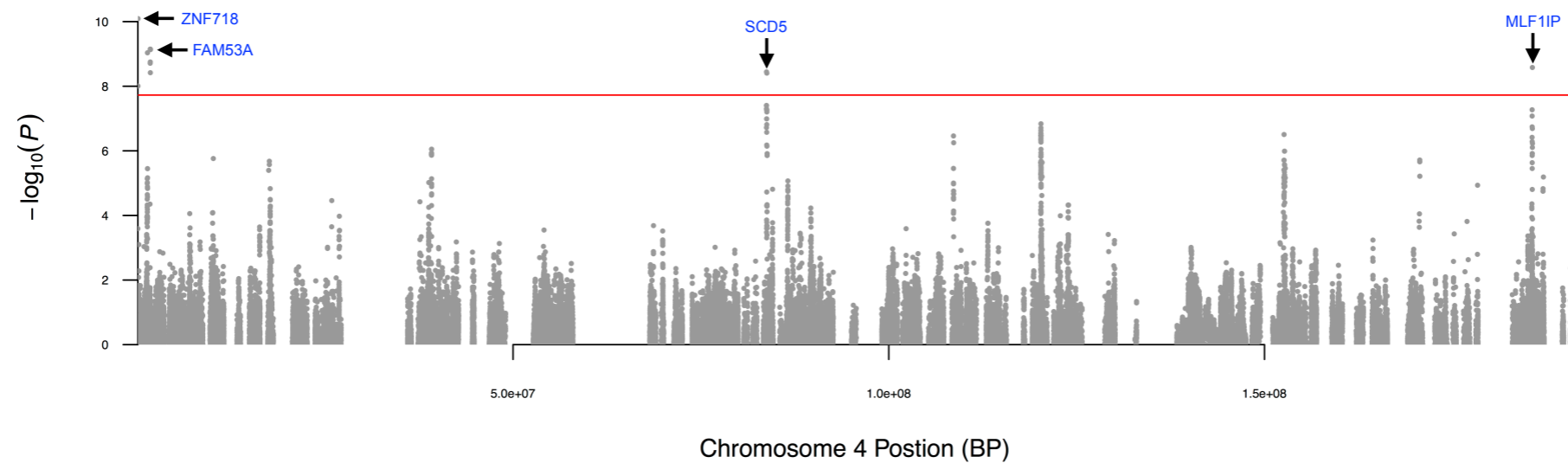

Supplement: S19 Fig — Depicted are the −log10(P) transformed MAPIT p-values of quality-control-positive cis-SNPs plotted against their genomic position in chromosomes (A) 2, (B) 3, and (C) 4, respectively. Note that MAPIT was implemented with Kcis. Here, the epistatic associated genes are labeled (blue). The (red) horizontal line indicates a genome-wide significance threshold (P = 1.828 × 10^−8^). Note that all panels are truncated at −log10(P) = 10 for consistency and presentation, although for some genes there are strongly marginally epistatic associated markers with p-values P ≈ 0. (PDF) [file pgen.1006869.s019.pdf]

A

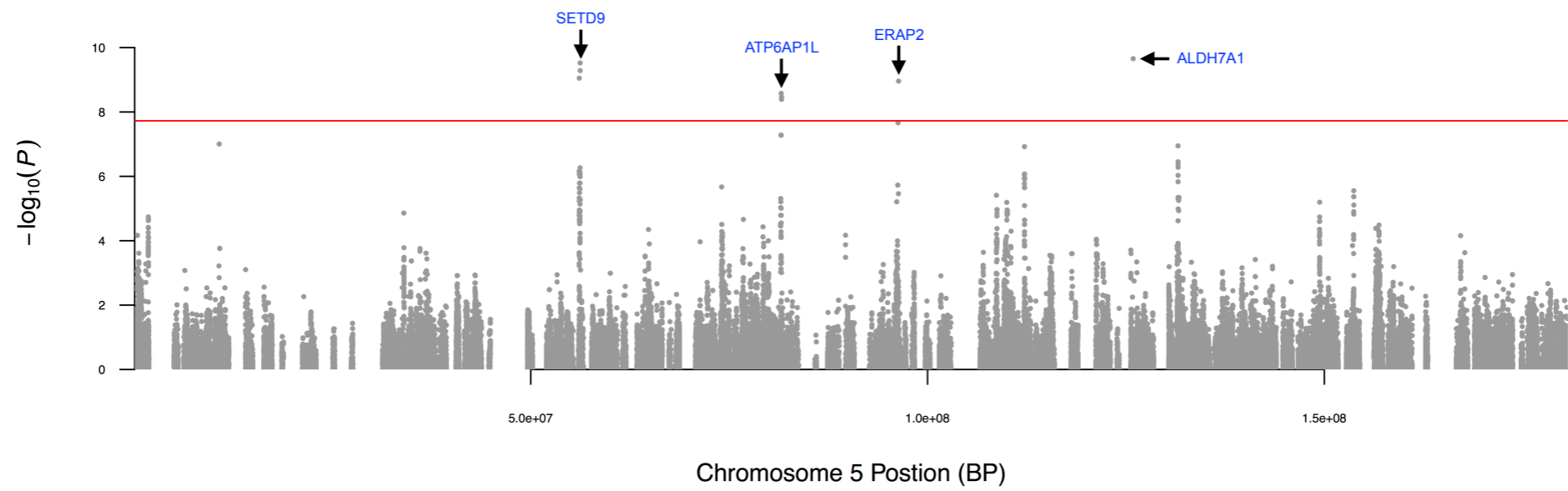

B

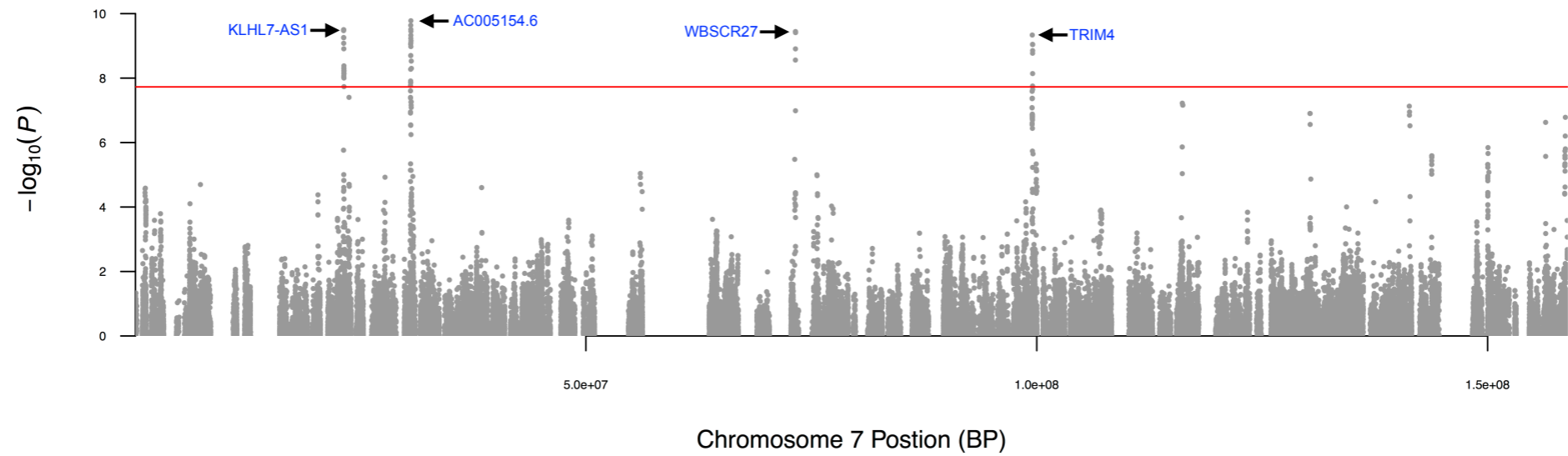

C

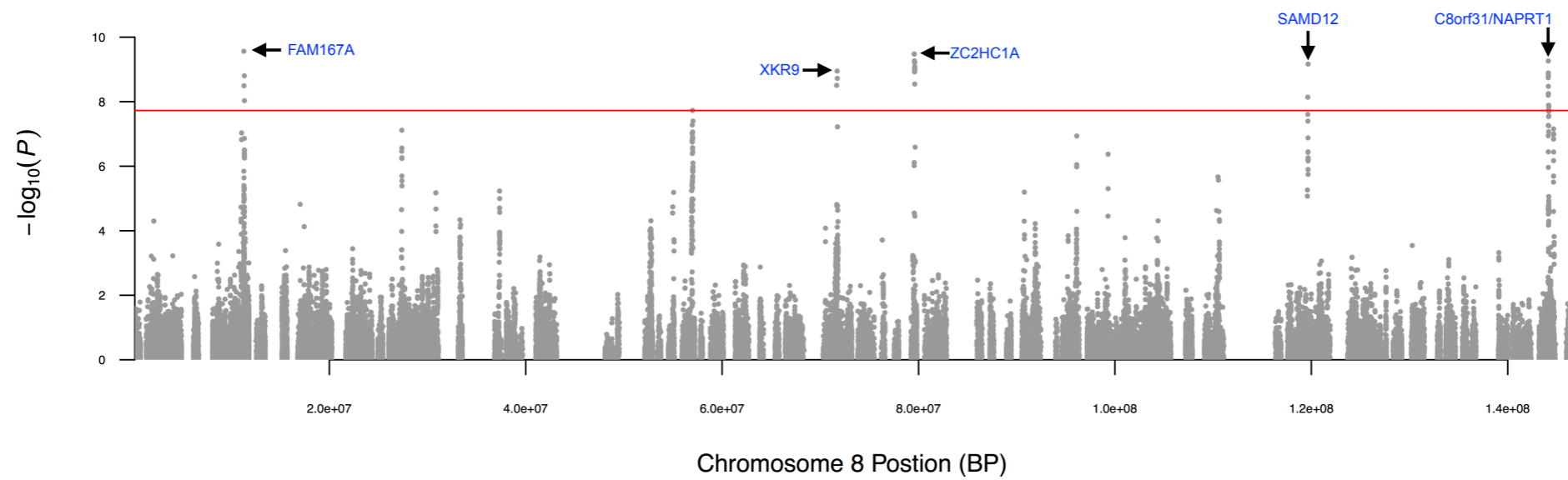

Supplement: S20 Fig — Depicted are the −log10(P) transformed MAPIT p-values of quality-control-positive cis-SNPs plotted against their genomic position in chromosomes (A) 5, (B) 7, and (C) 8, respectively. Note that MAPIT was implemented with Kcis. Here, the epistatic associated genes are labeled (blue). The (red) horizontal line indicates a genome-wide significance threshold (P = 1.828 × 10^−8^). Note that all panels are truncated at −log10(P) = 10 for consistency and presentation, although for some genes there are strongly marginally epistatic associated markers with p-values P ≈ 0. (PDF) [file pgen.1006869.s020.pdf]

A

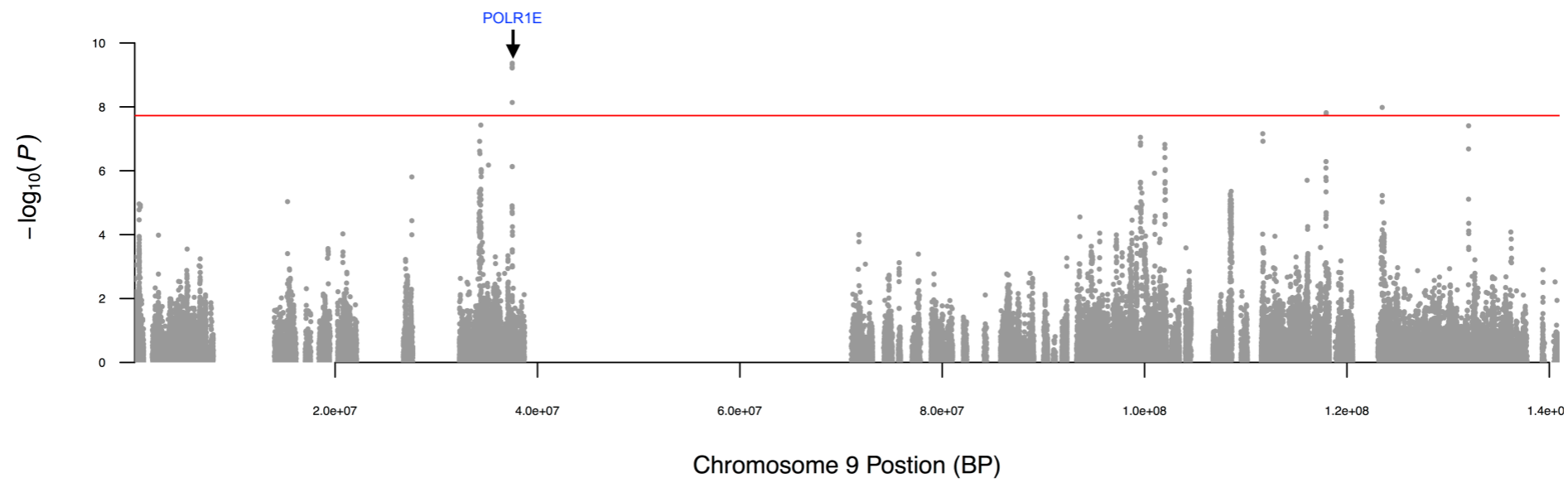

B

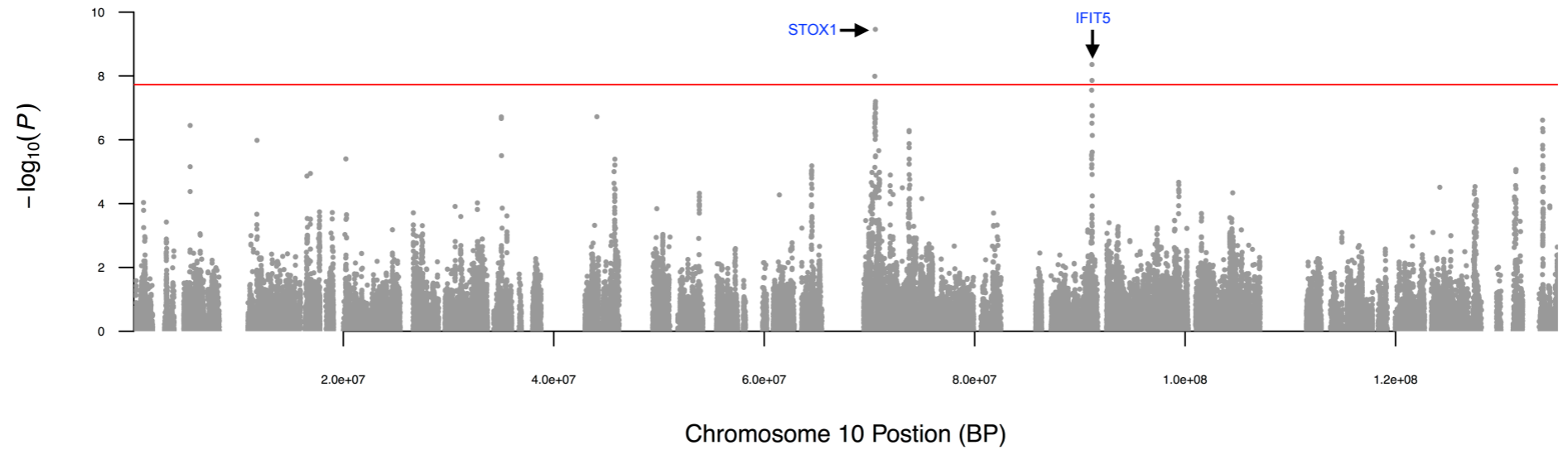

C

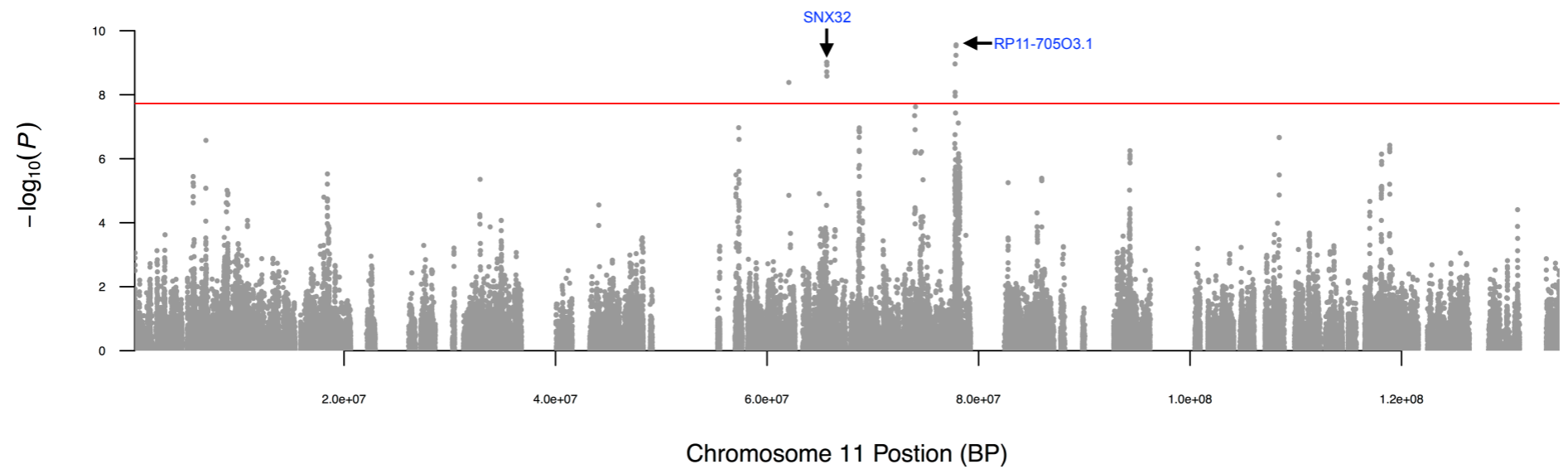

Supplement: S21 Fig — Depicted are the −log10(P) transformed MAPIT p-values of quality-control-positive cis-SNPs plotted against their genomic position in chromosomes (A) 9, (B) 10, and (C) 11, respectively. Note that MAPIT was implemented with Kcis. Here, the epistatic associated genes are labeled (blue). The (red) horizontal line indicates a genome-wide significance threshold (P = 1.828 × 10^−8^). Note that all panels are truncated at −log10(P) = 10 for consistency and presentation, although for some genes there are strongly marginally epistatic associated markers with p-values P ≈ 0. (PDF) [file pgen.1006869.s021.pdf]

A

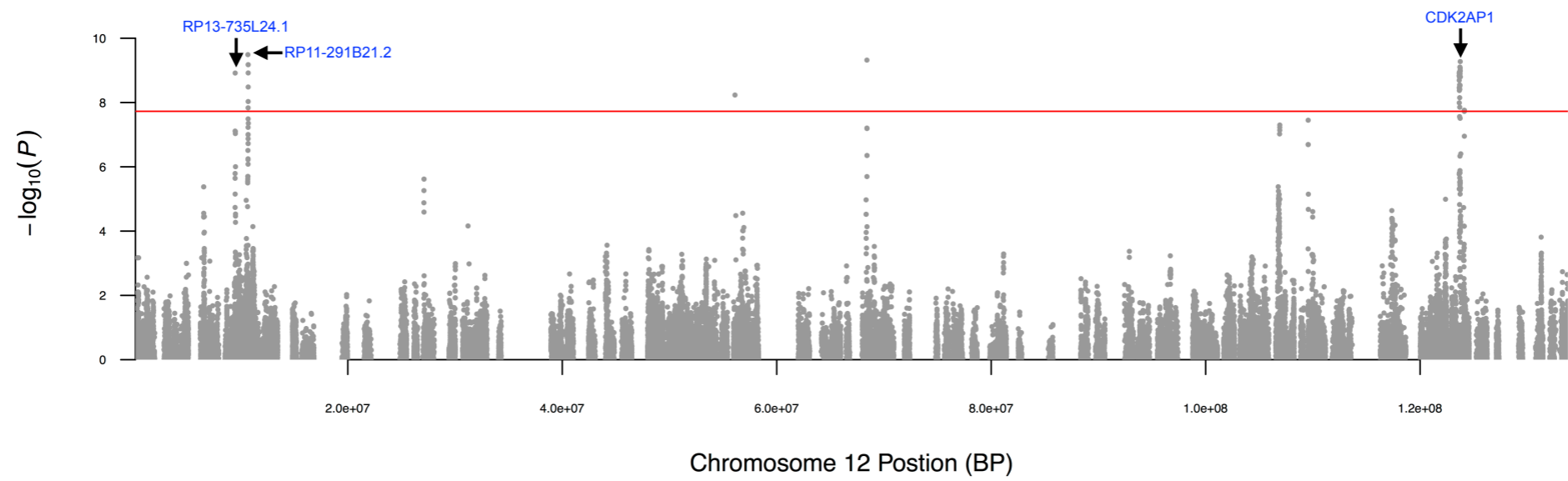

B

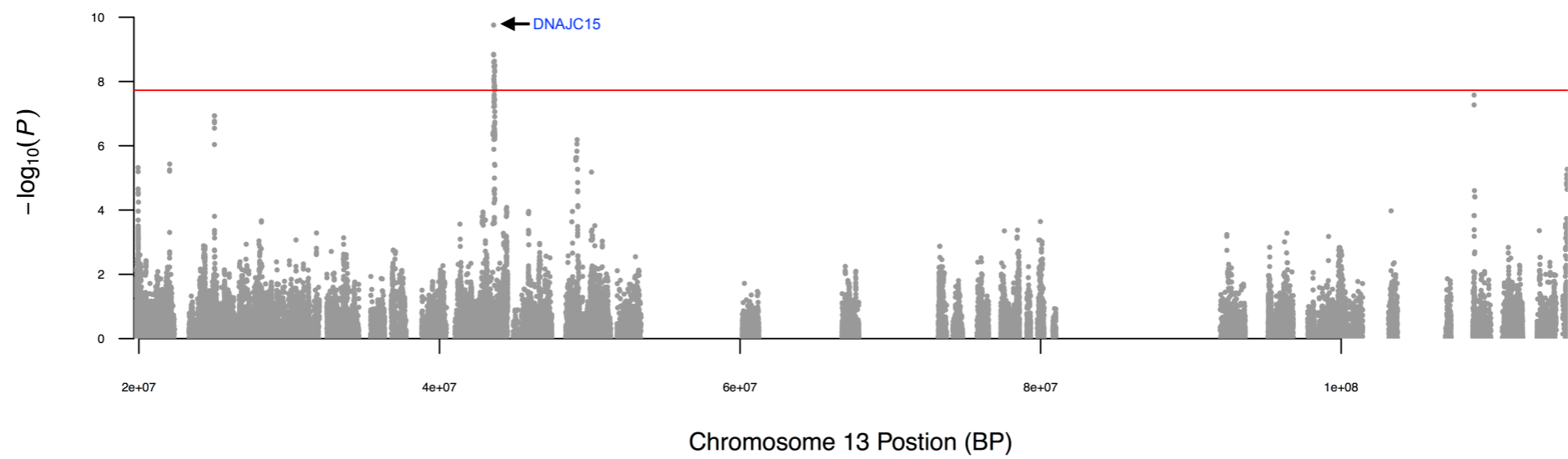

C

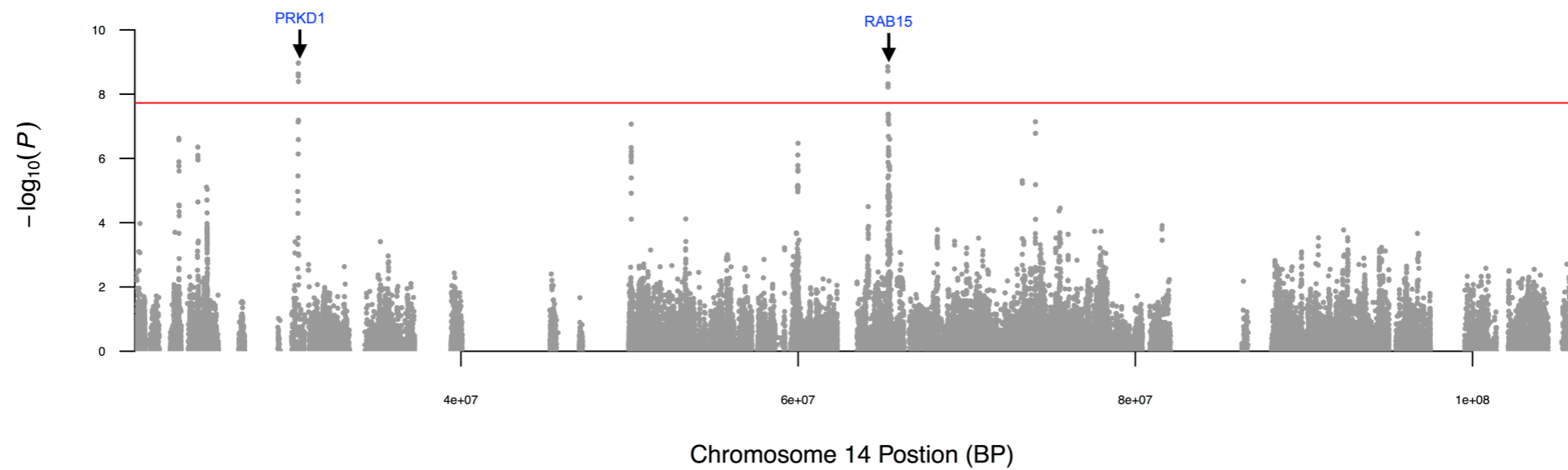

Supplement: S22 Fig — Depicted are the −log10(P) transformed MAPIT p-values of quality-control-positive cis-SNPs plotted against their genomic position in chromosomes (A) 12, (B) 13, and (C) 14, respectively. Note that MAPIT was implemented with Kcis. Here, the epistatic associated genes are labeled (blue). The (red) horizontal line indicates a genome-wide significance threshold (P = 1.828 × 10^−8^). Note that all panels are truncated at −log10(P) = 10 for consistency and presentation, although for some genes there are strongly marginally epistatic associated markers with p-values P ≈ 0. (PDF) [file pgen.1006869.s022.pdf]

A

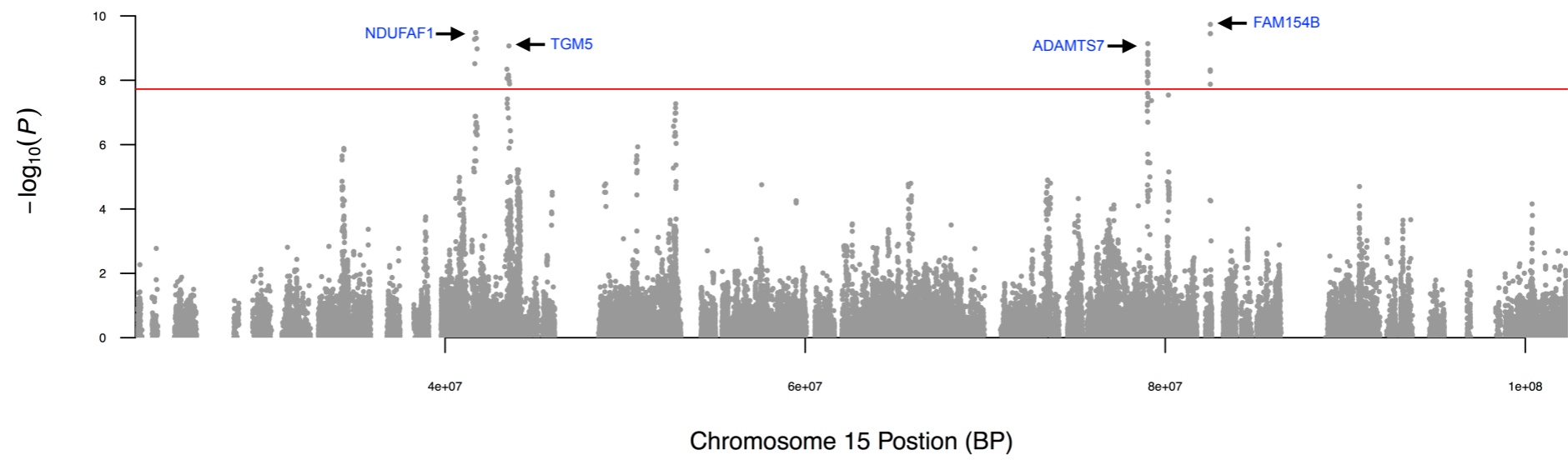

B

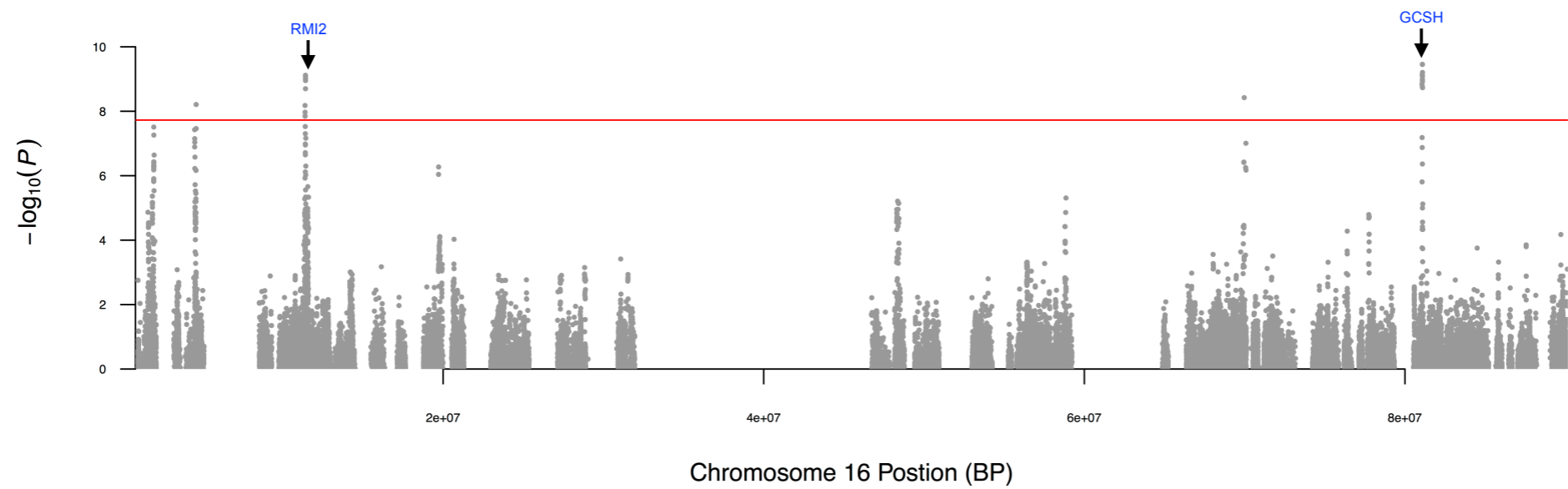

C

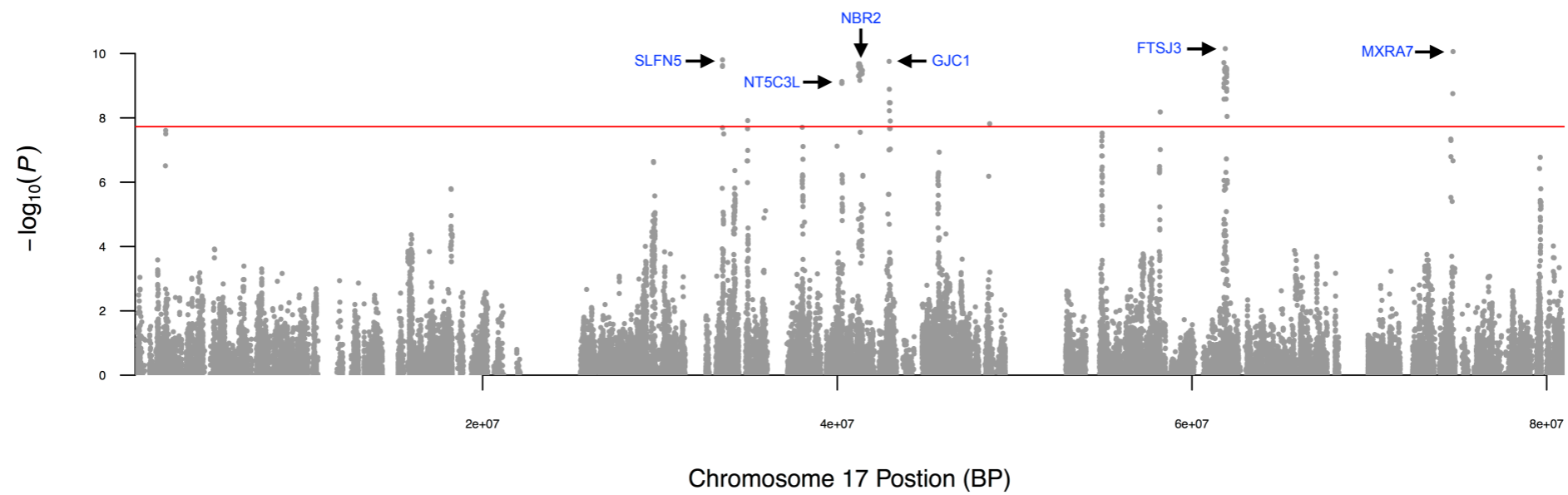

Supplement: S23 Fig — Depicted are the −log10(P) transformed MAPIT p-values of quality-control-positive cis-SNPs plotted against their genomic position in chromosomes (A) 15, (B) 16, and (C) 17, respectively. Note that MAPIT was implemented with Kcis. Here, the epistatic associated genes are labeled (blue). The (red) horizontal line indicates a genome-wide significance threshold (P = 1.828 × 10^−8^). Note that all panels are truncated at −log10(P) = 10 for consistency and presentation, although for some genes there are strongly marginally epistatic associated markers with p-values P ≈ 0. (PDF) [file pgen.1006869.s023.pdf]

A

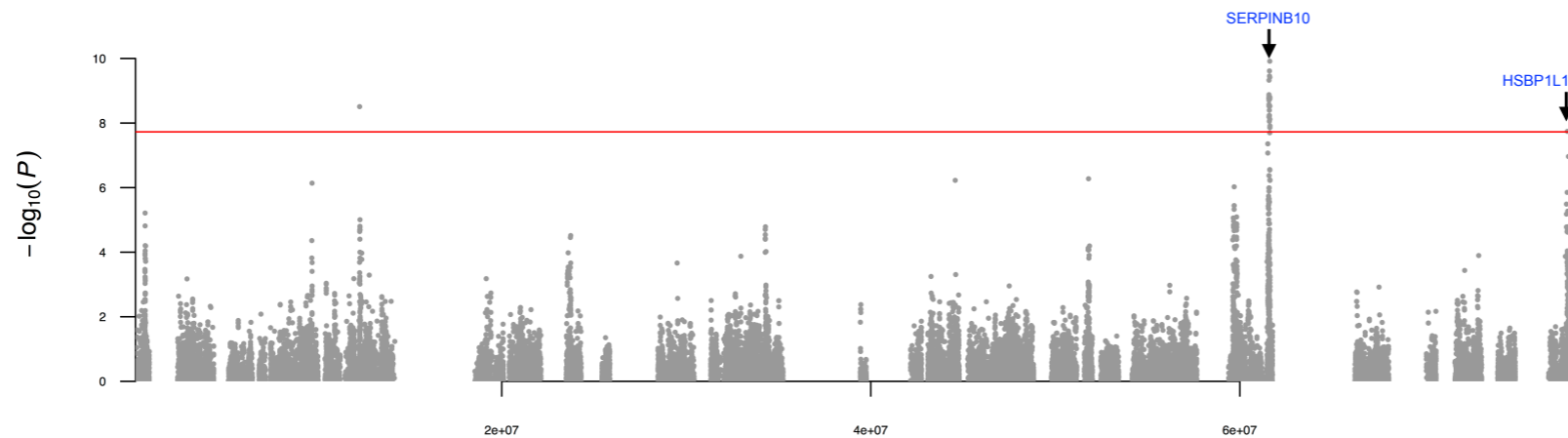

B

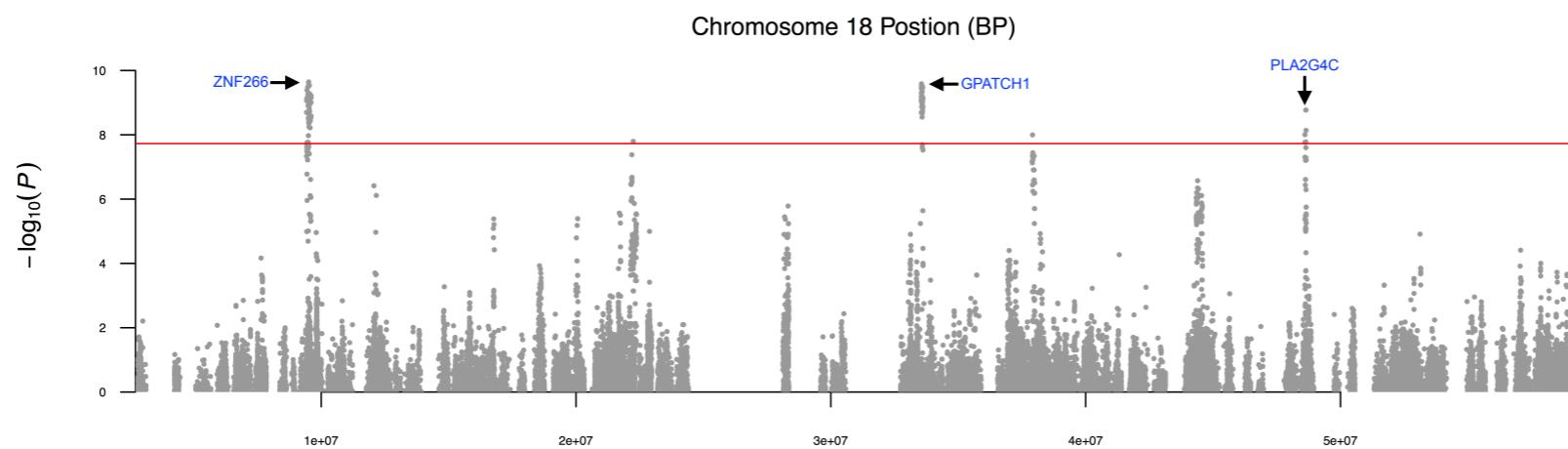

C

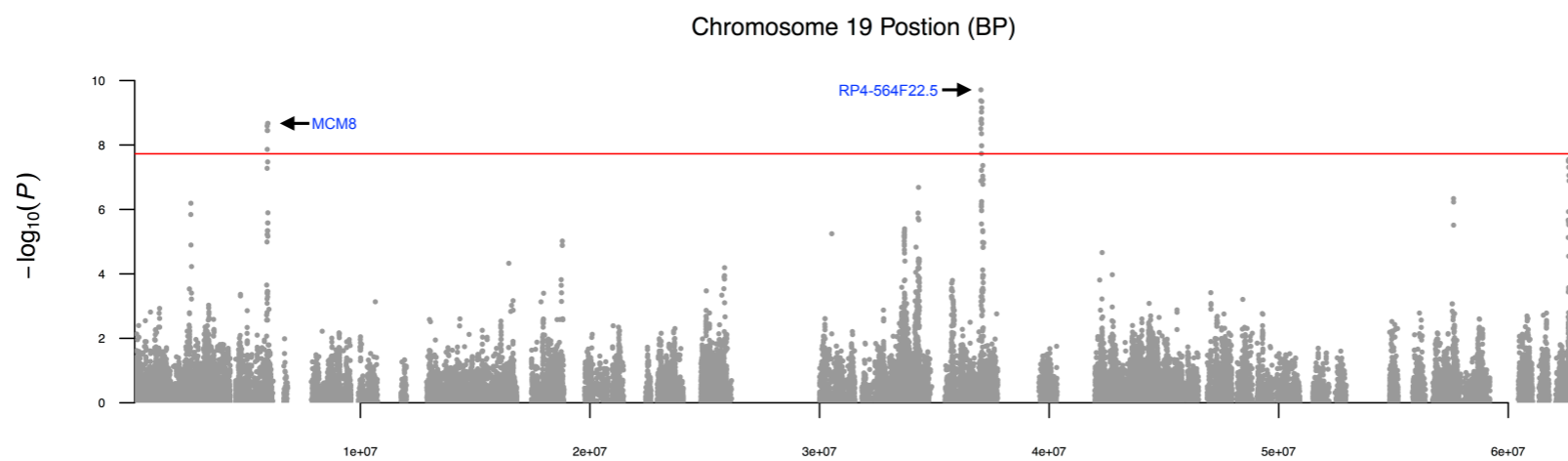

D

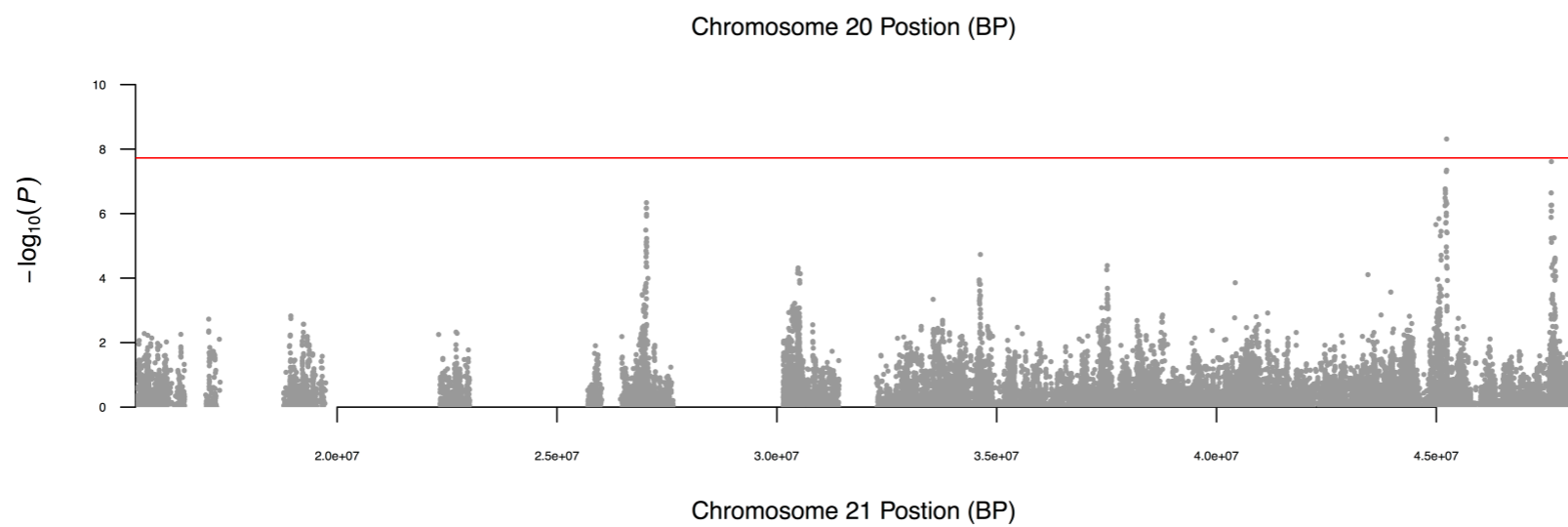

Supplement: S24 Fig — Depicted are the −log10(P) transformed MAPIT p-values of quality-control-positive cis-SNPs plotted against their genomic position in chromosomes (A) 18, (B) 19, (C) 20, and (D) 21 respectively. Note that MAPIT was implemented with Kcis. Here, the epistatic associated genes are labeled (blue). The (red) horizontal line indicates a genome-wide significance threshold (P = 1.828 × 10^−8^). Note that all panels are truncated at −log10(P) = 10 for consistency and presentation, although for some genes there are strongly marginally epistatic associated markers with p-values P ≈ 0. (PDF) [file pgen.1006869.s024.pdf]

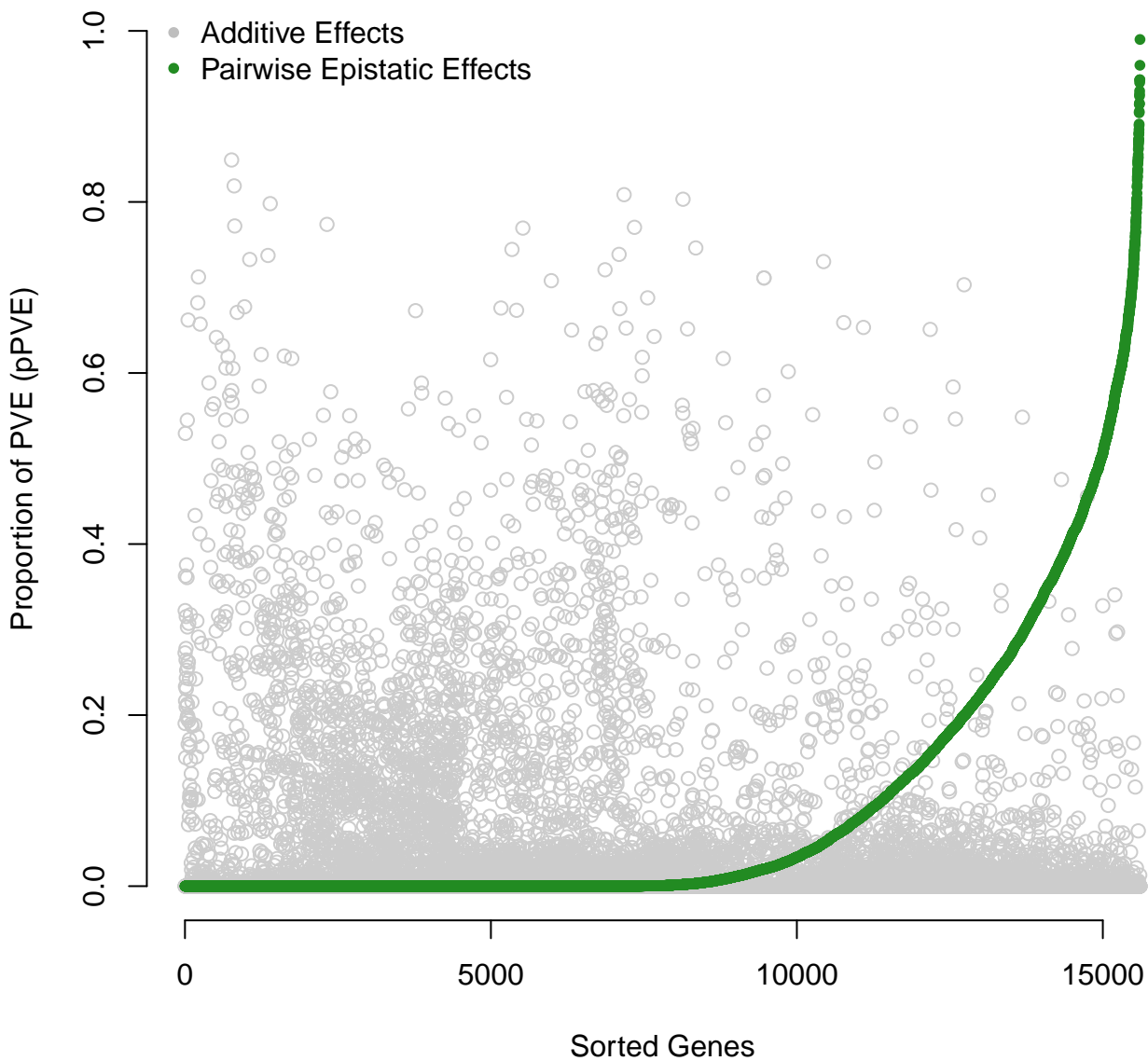

Supplement: S25 Fig — Estimates of the pPVE on the y-axis were calculated by using variance component models, where each of the components represent for additive effects (grey) and pairwise epistasis (green). More specifically, the variance components correspond to additive and pairwise epistatic covariance matrices K and K2, respectively. Note that K2 = K ∘ K is obtained by using the Hadamard product (i.e. the squaring of each element) of the matrix K. See Methods and Material for details. (PDF) [file pgen.1006869.s025.pdf]
