# Supplementary material for: Detecting epistasis with the marginal epistasis test in genetic mapping studies of quantitative traits
Source: PLoS Genet. 2017 Jul 26;13(7):e1006869. doi: 10.1371/journal.pgen.1006869 (PMC5550000; doi:10.1371/journal.pgen.1006869)
Supplement: S1 Table — Each entry represents the mean computation time (in minutes) it takes to run MAPIT under different hypothesis testing strategies. These tests are the normal test, and the Davies method for approximating a mixture of chi-squares. Computations were performed using 32 cores on Duke University’s Center for Genomic and Computational Biology HARDAC Cluster. To create genetic data for these simulations, we generated 5 × 10^3^, 1 × 10^4^, 5 × 10^4^, and 1 × 10^5^ genetic markers, respectively. Sample sizes were set to 1,000, 2,500, and 5,000. Values in the parentheses are the standard deviations of the estimates. (PDF) [file pgen.1006869.s026.pdf]

S1 Table

Table S1. Computational complexity for running MAPIT as a function of sample size and the number of SNPs. Each entry represents the mean computation time (in minutes) it takes to run MAPIT under different hypothesis testing strategies. These tests are the normal test, and the Davies method for approximating a mixture of chi-squares. Computations were performed using 32 cores on Duke University's Center for Genomic and Computational Biology HARDAC Cluster. To create genetic data for these simulations, we generated 5×10^3, 1×10^4, 5×10^4, and 1×10^5 genetic markers, respectively. Sample sizes were set to 1,000, 2,500, and 5,000. Values in the parentheses are the standard deviations of the estimates.

	Average Time (min)				
Test	Total Sample Size	5×10^3 Markers	1×10^4 Markers	5×10^4 Markers	1×10^5 Markers
Normal Test	$n = 1{,}000$	1.0 (0.01)	2.1 (0.01)	10.5 (0.15)	20.8 (0.05)
	$n = 2{,}500$	9.5 (0.03)	18.9 (0.08)	94.3 (0.63)	186.3 (3.53)
	$n = 5{,}000$	33.8 (0.13)	67.6 (0.17)	352.1 (9.74)	688.5 (19.05)
Davies Method	$n = 1{,}000$	3.0 (0.01)	6.1 (0.01)	30.3 (0.03)	60.6 (0.08)
	$n = 2{,}500$	45.6 (3.96)	108.6 (25.36)	422.6 (26.42)	855.3 (43.83)
	$n = 5{,}000$	218.8 (34.94)	654.9 (10.37)	2578.9 (715.80)	4784.9 (2884.72)
