# Supplementary material for: Detecting epistasis with the marginal epistasis test in genetic mapping studies of quantitative traits
Source: PLoS Genet. 2017 Jul 26;13(7):e1006869. doi: 10.1371/journal.pgen.1006869 (PMC5550000; doi:10.1371/journal.pgen.1006869)
Supplement: S2 Table — Each entry represents type I error rate estimates as the proportion of p-values a under the null hypothesis based on 100 simulated continuous phenotypes for the normal test (or z-test) and the Davies method. These results are based on 100 simulated data sets using simulation model (ii) with the top 5 genotype PCs. Recall that model (ii) is used to evaluate the type I error control of MAPIT when there is population stratification. Empirical size for the analyses used significance thresholds of α = 0.05, 0.01, and 0.001. Sample sizes were set to 1,000, 1,750, and 2,500. Values in the parentheses are the standard deviations of the estimates. (PDF) [file pgen.1006869.s027.pdf]

S2 Table

Table S2. Empirical type I error estimates of MAPIT in the presence of population stratification effects (Top 5 PCs). Each entry represents type I error rate estimates as the proportion of p-values a under the null hypothesis based on 100 simulated continuous phenotypes for the normal test (or z-test) and the Davies method. These results are based on 100 simulated data sets using simulation model (ii) with the top 5 genotype PCs. Recall that model (ii) is used to evaluate the type I error control of MAPIT when there is population stratification. Empirical size for the analyses used significance thresholds of $\alpha = 0.05$, 0.01, and 0.001. Sample sizes were set to 1,000, 1,750, and 2,500. Values in the parentheses are the standard deviations of the estimates.

Test	Total Sample Size	$\alpha = 0.05$	$\alpha = 0.01$	$\alpha = 0.001$
Normal Test	$n = 1{,}000$	0.0605 (0.0099)	0.0205 (0.0037)	0.0060 (0.0013)
	$n = 1{,}750$	0.0588 (0.0073)	0.0176 (0.0034)	0.0042 (0.0008)
	$n = 2{,}500$	0.0531 (0.0058)	0.0146 (0.0019)	0.0028 (0.0005)
Davies Method	$n = 1{,}000$	0.0572 (0.0133)	0.0119 (0.0044)	0.0011 (0.0008)
	$n = 1{,}750$	0.0508 (0.0086)	0.0103 (0.0026)	0.0010 (0.0004)
	$n = 2{,}500$	0.0510 (0.0046)	0.0107 (0.0016)	0.0009 (0.0003)
