# Supplementary material for: Detecting epistasis with the marginal epistasis test in genetic mapping studies of quantitative traits
Source: PLoS Genet. 2017 Jul 26;13(7):e1006869. doi: 10.1371/journal.pgen.1006869 (PMC5550000; doi:10.1371/journal.pgen.1006869)
Supplement: S3 Table — Each entry represents type I error rate estimates as the proportion of p-values a under the null hypothesis based on 100 simulated continuous phenotypes for the normal test (or z-test) and the Davies method. These results are based on 100 simulated data sets using simulation model (ii) with the top 10 genotype PCs. Recall that model (ii) is used to evaluate the type I error control of MAPIT when there is population stratification. Empirical size for the analyses used significance thresholds of α = 0.05, 0.01, and 0.001. Sample sizes were set to 1,000, 1,750, and 2,500. Values in the parentheses are the standard deviations of the estimates. (PDF) [file pgen.1006869.s028.pdf]

S3 Table

Table S3. Empirical type I error estimates of MAPIT in the presence of population stratification effects (Top 10 PCs). Each entry represents type I error rate estimates as the proportion of p-values a under the null hypothesis based on 100 simulated continuous phenotypes for the normal test (or z-test) and the Davies method. These results are based on 100 simulated data sets using simulation model (ii) with the top 10 genotype PCs. Recall that model (ii) is used to evaluate the type I error control of MAPIT when there is population stratification. Empirical size for the analyses used significance thresholds of $\alpha = 0.05$, 0.01, and 0.001. Sample sizes were set to 1,000, 1,750, and 2,500. Values in the parentheses are the standard deviations of the estimates.

Test	Total Sample Size	$\alpha = 0.05$	$\alpha = 0.01$	$\alpha = 0.001$
Normal Test	$n = 1{,}000$	0.0564 (0.0124)	0.0183 (0.0045)	0.0050 (0.0013)
	$n = 1{,}750$	0.0556 (0.0058)	0.0169 (0.0024)	0.0040 (0.0007)
	$n = 2{,}500$	0.0566 (0.0057)	0.0158 (0.0023)	0.0034 (0.0007)
Davies Method	$n = 1{,}000$	0.0538 (0.0126)	0.0111 (0.0043)	0.0012 (0.0005)
	$n = 1{,}750$	0.0508 (0.0061)	0.0102 (0.0023)	0.0012 (0.0004)
	$n = 2{,}500$	0.0470 (0.0050)	0.0090 (0.0019)	0.0007 (0.0005)
